# Equation of state and thermodynamic properties for mixtures of $H_{2}O$, $O_{2}$, $N_{2}$, and ${\mathrm{CO}}_{2}$ from ambient up to 1000 K and 280 MPa

**DOI:** 10.1016/j.supflu.2019.02.016

**Published:** 2019-11

**Authors:** F. Mangold, St. Pilz, S. Bjelić, F. Vogel

**Affiliations:** a University of Applied Sciences and Arts Northwestern Switzerland FHNW, School of Engineering, Klosterzelgstrasse 2, 5210 Windisch, Switzerland; b by then DaimlerChrysler, Research and Technology (FT4/TP), 89013 Ulm, Germany; c Paul Scherrer Institute PSI, 5232 Villigen PSI, Switzerland

**Keywords:** Equation of state, Thermodynamic properties, Supercritical, Mixture, Volume translation

## Abstract

Supercritical water oxidation (SCWO) is an effective technique to treat wet organic wastes. Its modeling requires an accurate calculation of thermodynamic properties. In this work an equation of state (EOS) is proposed which accurately predicts the thermodynamic state of mixtures of water, oxygen, nitrogen, and carbon dioxide for a wide range of compositions, temperatures, and pressures including supercritical conditions. The EOS includes a volume translation, an evolved α-function and non-quadratic mixing rules. The introduced parameters are regressed to experimental data. From the pressure-explicit EOS, enthalpy, specific heats at constant volume and constant pressure, and fugacity coefficients are derived and calculated. The binary mixtures $H_{2}O/O_{2}$, $H_{2}O/N_{2}$, $H_{2}O/{\mathrm{CO}}_{2}$, $N_{2}/{\mathrm{CO}}_{2}$ as well as the ternary mixture $H_{2}O/O_{2}/N_{2}$ are well predicted by the proposed EOS with relative errors below 10% and 15%, respectively. The region of low temperature and high pressure is most difficult to predict with relative errors up to 20%.

## Highlights

• States of oxygen and nitrogen are predicted with high accuracy.
• Water and ${\mathrm{CO}}_{2}$ are more difficult to predict including their critical points.
• Binary mixtures are suitably predicted, as well as the selected ternary mixture.
• Errors are relatively low except close to the vapor-liquid coexistence-curve.

## Graphical abstract

**Figure:**
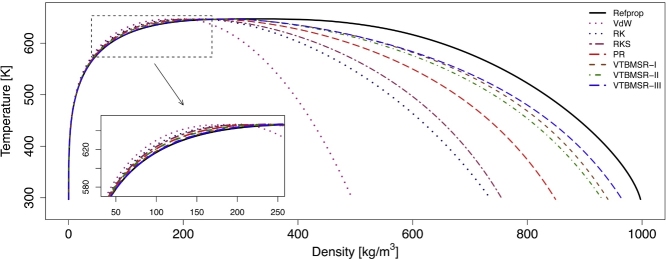

## List of symbols

**Abbreviations**
BM: Boston Mathias
EOS: Equation of state
HTO: Hydrothermal oxidation
NIST: National Institute of Standards and Technology
PR: Peng-Robinson
RK: Redlich-Kwong
RKS: Redlich-Kwong-Soave
SCWO: Supercritical water oxidation
VdW: Van der Waals
VTBMSR: Volume-Translation-Boston-Mathias- Schwartzentruber-Renon

**Roman letters**
a: Attraction term
b: Co-volume term
c: Volume translation
$c_{i}$: Volume translation parameters
$c_{d}$: Parameter of VTBMSR-EOS
$c_{p}$: Heat capacity at constant pressure
$c_{v}$: Heat capacity at constant volume
d: Parameter of VTBMSR-EOS
e: Error
f: Fugacity
h: Enthalpy
$k_{a}$: Binary interaction parameter related to attraction term
$k_{a}^{\left(z\right)}$: Binary interaction parameter coefficient related to attraction term
$k_{b}$: Binary interaction parameter related to co-volume term
$k_{b}^{\left(z\right)}$: Binary interaction parameter coefficient related to co-volume term
l: Binary interaction parameter related to attraction term
$l^{\left(z\right)}$: Binary interaction parameter coefficient related to attraction term
m: Parameter of EOS, f(ω)
N: Number of compounds
p: Pressure
$p_{i}$: Polar parameters
R: Universal gas constant
T: Temperature
u: Parameter of generalized EOS
u: Internal energy
v: Molar volume
w: Parameter of generalized EOS
x: Molar fraction
y: Molar fraction (gas phase)

**Greek letters**
α: Temperature-dependent part of a
α,β,γ,δ,ε: Parameters for polynomial $c_{p}^{0}(T)$
ρ: Density
ϕ: Fugacity coefficient
ω: Acentric factor
Ω: Unitless constant of EOS

**Super/subscripts**
0: Reference state
a: Attraction term
b: Co-volume term
c: Critical
calc: Calculated
i: Mixture compound; index
ig: Ideal gas
j: Mixture compound; index
l: Liquid
mix: Mixture property
r: Reduced
ref: Reference
rel: Relative
res: Residual
sc: Supercritical
tot: Total
v: Vapor
z: Index

## 1 Introduction

Supercritical water oxidation (SCWO), also referred to as hydrothermal oxidation (HTO), operates at conditions above the critical point of water ($T_{c}=647.14$ K, $p_{c}=22.064$ MPa) [1]. Typically, temperature and pressure are within the range of 673–923 K and 22–35 MPa, respectively. SCWO is of particular interest for feedstocks with high water content (*e.g.* sewage sludge, paper sludge, *etc.*) as there is no requirement for an energy intensive drying prior to the process [2]. Within residence times of up to a few minutes, the feed material is completely decomposed mainly to carbon dioxide, water, nitrogen/ammonia and minerals [3]. Compared to combustion, the rates of reaction between nitrogen and oxygen are low, thus no nitrogen oxide is formed [3]. Supercritical water is an interesting reaction medium due to its miscibility with gases, such as oxygen, nitrogen, and carbon dioxide enabling a homogeneous reaction and high reaction rates [4].

The modeling of an SCWO process requires an accurate calculation of the thermodynamic properties such as temperature, pressure, molar volume, enthalpy, heat capacity, and fugacity [4]. Over the broad range of temperatures and pressures from ambient conditions up to 923 K and 35 MPa of an SCWO process, the mixture including the four main substances water, oxygen, nitrogen, and carbon dioxide behaves highly non-ideally. Modeling of an SCWO includes the task of predicting the state around the critical point as well as the description of water in its liquid form. Thereby, implying the need to accurately predict densities over a wide range. Additionally, water changes its strong polar character to a moderate one in the supercritical state [4], [5]. These characteristics of an SCWO process induce challenging requirements which have to be satisfied to model the thermodynamic properties appropriately [4].

Equations of state (EOS) are an approach to describe the states of non-ideal gases and their thermodynamic properties [4]. Van der Waals developed the first EOS describing the liquid and vapor density as well as the phenomena of the critical point by a cubic equation [6]. The EOS considers the attraction between the molecules and the co-volume due to the spatial expansion of the molecules. It enables the prediction of the liquid-vapor equilibrium for pure compounds and reduces to the equation for an ideal gas for high temperatures and low densities. The prediction of the pvT state becomes less accurate the higher the density of the fluid is. Redlich and Kwong improved this EOS by extending the attraction term with a temperature dependency (α(T)) [7]. Soave introduced a different temperature dependency including the acentric factor (ω) as a third parameter with respect to the critical temperature and critical pressure resulting in the Redlich–Kwong–Soave (RKS) EOS [8]. The accuracy for non-polar substances with high acentric factors is improved whereas the densities of liquids are still difficult to predict. A further modification of the attraction term by Peng and Robinson (PR) provides a more accurate prediction of the liquid densities [9]. The mentioned EOS have the same generalized cubic structure:

(1) $$p=\frac{R\;T}{v-b}-\frac{a(T)}{v^{2}+u\;b\;v-w\;b^{2}}$$

with the parameters $a(T)=a_{c}\;\alpha (T)$, $a_{c}=\Omega_{a}\;\frac{R^{2}\;T_{c}^{2}}{p_{c}}$ and $b=b_{c}=\Omega_{b}\;\frac{R\;T_{c}}{p_{c}}$. The equation-dependent parameters α(T), u and w, as well as the unitless constants $\Omega_{a}$ and $\Omega_{b}$, are given in Table 1. The RKS and PR EOS are widely used, especially for hydrocarbon systems [10], [11]. Martin introduced a volume translation c which increases the accuracy of the density prediction over a wide range of temperatures and pressures including the liquid and gas phases [12]. By distinguishing the sub- and supercritical region, Boston and Mathias extended the range of application of α(T) [13]. Mathias improved these relations by modifications to highly polar substances such as water by introducing a polar parameter in α(T) [14]. A further improvement was achieved by Schwartzentruber and Renon by introducing three polar parameters [15]. Temperature-dependent volume translation can lead to isothermal crossing, *i.a*. observable in negative heat capacities [16], [17], [18], [19], [20]. Le Guennec et al. proposed translated-consistent equations of state with consistent α-functions and temperature-independent volume translation for an accurate prediction of thermodynamic properties by eliminating isothermal crossing and discontinuities in α and its derivatives [21].

**Table 1:** Parameters and α-functions of different cubic EOS. Abbreviations of EOS: Van der Waals (VdW), Redlich–Kwong (RK), Redlich–Kwong–Soave (RKS), Peng–Robinson (PR), Boston–Mathias (BM), Volume-Translation-Boston-Mathias-Schwartzentruber-Renon (VTBMSR).

EOS	Reference	u	w	α(T)	$\Omega_{a}$	$\Omega_{b}$
VdW	[1], [11]	0	0	1	0.421875	0.125
RK	[1], [11]	1	0	$1/\sqrt{T_{r}}$	0.4275	0.08664
RKS	[1], [11]	1	0	${\left[1+m\;(1-\sqrt{T_{r}})\right]}^{2}$	0.42748	0.08664
				$m=0.48+1.574\;\omega -0.176\;\omega^{2}$		
PR	[1], [9]	2	1	${\left[1+m\;(1-\sqrt{T_{r}})\right]}^{2}$	0.45724	0.07780
				$m=0.37464+1.54226\;\omega -0.26992\;\omega^{2}$		
BM (PR)	[13]	2	1	i) $T_{r}\leq 1$	0.45724	0.07780
				${\left[1+m\;(1-\sqrt{T_{r}})\right]}^{2}$		
				ii) $T_{r}>1$		
				$\exp\{c_{d}(1-T_{r}^{d})\}$		
				$m=0.37464+1.54226\;\omega -0.26992\;\omega^{2}$		
				d=1+m/2		
				$c_{d}=m/d$		
Mathias	[14]	1	0	i) $T_{r}\leq 1$	0.42727	0.08864
				${\left[1+m\;(1-\sqrt{T_{r}})-p_{i}(1-T_{r})(0.7-T_{r})\right]}^{2}$		
				ii) $T_{r}>1$		
				${\left[\exp\{c_{d}(1-T_{r}^{d})\}\right]}^{2}$		
				$m=0.48508+1.55191\;\omega -0.15613\;\omega^{2}$		
				$c_{d}=1+m/2+0.3\;p_{i}$		
				$d=(c_{d}-1)/c_{d}$		
VTBMSR-I	[26]	1	0	i) $T_{r}\leq 1$	0.42748	0.08664
				${\left[\exp\{c_{d}(1-T_{r}^{d})\}\right]}^{2}$		
				ii) $T_{r}>1$		
				${\left[1+m\;(1-\sqrt{T_{r}})-p_{0}(1-T_{r})(1+p_{1}\;T_{r}+p_{2}\;T_{r}^{2})\right]}^{2}$		
				$m=0.48508+1.55191\;\omega -0.15613\;\omega^{2}$		
				$c_{d}=1-1/d$		
				$d=1+0.5\;m-p_{0}(1+p_{1}+p_{2})$		
VTBMSR-II	[11]	1	0	i) $T_{r}\leq 1$	0.42748	0.08664
				${\left[\exp\{c_{d}(1-T_{r})\}\right]}^{2}$		
				ii) $T_{r}>1$		
				${\left[1+m\;(1-\sqrt{T_{r}})-p_{0}(1-T_{r})(1+p_{1}\;T_{r}+p_{2}\;T_{r}^{2})\right]}^{2}$		
				$m=0.48508+1.55191\;\omega -0.15613\;\omega^{2}$		
				$c_{d}=1-1/d$		
				$d=1+0.5\;m-p_{0}(1+p_{1}+p_{2})$		
VTBMSR-III	this work	1	0	i) $T_{r}\leq 1$	0.42748	0.08664
				${\left[1+m\;(1-\sqrt{T_{r}})-p_{0}(1-T_{r})(1+p_{1}\;T_{r}+p_{2}\;T_{r}^{2})\right]}^{2}$		
				ii) $T_{r}>1$		
				${\left[\exp\{c_{d}(1-T_{r}^{d})\}\right]}^{2}$		
				$m=0.48508+1.55191\;\omega -0.15613\;\omega^{2}$		
				$c_{d}=1-1/d$		
				$d=1+0.5\;m-p_{0}(1+p_{1}+p_{2})$		

The equation of state for a pure compound is extended by the concept of an one-fluid mixture. It is assumed for a fixed composition, that the mixture properties and their variations with temperature and pressure can be described like a pure compound with adjusted parameters based on the composition of the mixture [1]. A basic approach for the determination of the mixture parameters $a_{\mathrm{mix}}$ and $b_{\mathrm{mix}}$ are the conventional mixing rules where the mixing parameters have a quadratic dependence on composition (quadratic mixing rule) [20], [22], [23].

(2) $$a_{\mathrm{mix}}=\sum_{i=1}^{N}\sum_{j=1}^{N}x_{i}\;x_{j}\;a_{\mathrm{ij}}$$

(3) $$b_{\mathrm{mix}}=\sum_{i=1}^{N}\sum_{j=1}^{N}x_{i}\;x_{j}\;b_{\mathrm{ij}}$$

(4) $$c_{\mathrm{mix}}=\sum_{i=1}^{N}\sum_{j=1}^{N}x_{i}\;x_{j}\;c_{\mathrm{ij}}$$

The composition is described by the molar fractions of the compounds $x_{i}$ and $x_{j}$, respectively. The cross parameters $a_{\mathrm{ij}}$, $b_{\mathrm{ij}}$, and $c_{\mathrm{ij}}$ are closed by the combining rule which can vary in complexity. The simplest form is an arithmetic or geometric mean [1]. In these cases, the mixing rules reduce to linear dependence. For the cross parameters $a_{\mathrm{ij}}$ and $b_{\mathrm{ij}}$, the unweighted Lorentz–Berthelot combining rules are commonly applied [18]. Privat et al. stated that only the combining rule with the arithmetic mean fulfills the constraints for the mixed volume translation $\left(c_{\mathrm{ij}}\right)$ [20].

(5) $$a_{\mathrm{ij}}=\sqrt{a_{i}\;a_{j}}$$

(6) $$b_{\mathrm{ij}}=\frac{1}{2}\;(b_{i}+b_{j})$$

(7) $$c_{\mathrm{ij}}=\frac{1}{2}\;(c_{i}+c_{j})$$

These simple rules cannot adequately describe most mixtures, especially mixtures including liquids [1]. To improve $a_{\mathrm{ij}}$ and $b_{\mathrm{ij}}$, binary interaction parameters $k_{a,\mathrm{ij}}$ and $k_{b,\mathrm{ij}}$ are introduced to describe the deviation from the geometric mean and characterize the i-j interaction [24], [25].

(8) $$a_{\mathrm{ij}}=\sqrt{a_{i}\;a_{j}}\;(1-k_{a,\mathrm{ij}})$$

(9) $$b_{\mathrm{ij}}=\frac{1}{2}(b_{i}+b_{j})(1-k_{b,\mathrm{ij}})$$

The interaction parameters can be determined by fitting to experimental data. In addition to constant interaction parameters they can be formulated temperature-dependent ($k_{\mathrm{ij}}=f(T)$) [14]. Schwartzentruber and Renon further extended the interaction by a third interaction parameter $l_{\mathrm{ij}}$ and a dependence on the mole fraction of the compounds resulting in a non-quadratic mixing rule [15].

(10) $$a_{\mathrm{ij}}=\sqrt{a_{i}\;a_{j}}\;\left(1-k_{a,\mathrm{ij}}-(x_{i}-x_{j})\;l_{\mathrm{ij}}\right)$$

(11) $$b_{\mathrm{ij}}=\frac{1}{2}(b_{i}+b_{j})(1-k_{b,\mathrm{ij}})$$

For the interaction parameters $k_{a,\mathrm{ij}}$, $k_{b,\mathrm{ij}}$, and $l_{\mathrm{ij}}$ the following temperature dependence is assumed.

(12) $$k_{\mathrm{ij}}=k_{\mathrm{ij}}^{\left(0\right)}+k_{\mathrm{ij}}^{\left(1\right)}T+k_{\mathrm{ij}}^{\left(2\right)}/T$$

(13) $$l_{\mathrm{ij}}=l_{\mathrm{ij}}^{\left(0\right)}+l_{\mathrm{ij}}^{\left(1\right)}T+l_{\mathrm{ij}}^{\left(2\right)}/T$$

The interaction parameters $k_{\mathrm{ij}}^{\left(z\right)}$ and $l_{\mathrm{ij}}^{\left(z\right)}$ are determined by fitting to experimental data.

Based on these developments an appropriate EOS for modeling an SCWO process is developed and validated with available experimental data.

## 2 Development of the EOS

The characteristics of SCWO processes imply the following requirements on the EOS describing the thermodynamic behavior of the process [11]:

• Accurate prediction of density, enthalpy, and heat capacity of pure substances over a wide range of temperatures and pressures, especially for water
• Extension to mixtures of water, hydrocarbons, and gases without decreased accuracy
• Simple mathematical formulation to ensure numerical stability
• Explicit in pressure or volume, enabling derivation of further thermodynamic properties
• Minimal number of adjustable parameters, enabling stable and fast computation

So far none of the EOS in literature satisfies all these requirements [4], [21].

### 2.1 Developed equation of state

The EOS presented within this work is designed such as to best satisfy the aforementioned requirements. This is achieved by adding the following modifications to the cubic RKS EOS [7], [8]:

• Introduction of a temperature-dependent volume translation for accurate (liquid) density prediction [12]
• Representation of the polar character of water by introducing polar parameters in α [13], [14], [15]
• Application of non-quadratic mixing rules [15]

The resulting pressure-explicit EOS is referred to as VTBMSR EOS as presented by Pilz [11], [26]. The parameters of the VTBMSR EOS have, however, not yet been published.

(14) $$p=\frac{R\;T}{v+c-b}-\frac{a_{c}\;\alpha }{\left(v+c)(v+c+b\right)}$$

The constants $a_{c}$ and b are

(15) $$a_{c}=\frac{1}{9(2^{1/3}-1)}\;\frac{R^{2}\;T_{c}^{2}}{p_{c}}$$

(16) $$b=\frac{1}{3}(2^{1/3}-1)\;\frac{R\;T_{c}}{p_{c}}.$$

The volume translation c depends on the (reduced) temperature $T_{r}$ and the volume translation parameters $c_{i}$ [11], [26].

• $T_{r}\leq 1$
  (17) $$c=c_{0}+\frac{c_{1}}{1+c_{2}-T_{r}}$$
• $T_{r}\leq 1,c_{2}=T_{r}-1$
  (18) $$c=c_{0}$$
• $T_{r}>1,c_{1}=0$
  (19) $$c=c_{0}$$
• $T_{r}>1,c_{1}\neq 0$
  (20) $$c=b+\frac{{\left(\frac{(c_{0}-b)\;c_{2}}{c_{1}}+1\right)}^{2}\;c_{1}}{1+c_{2}\left(\frac{(c_{0}-b)\;c_{2}}{c_{1}}+1\right)-T_{r}}$$
• $T_{r}>1,c_{1}\neq 0,c_{2}\left(\frac{(c_{0}-b)\;c_{2}}{c_{1}}+1\right)=T_{r}-1$
  (21) $$c=c_{0}$$

Eqs. (18) and (21) are added to the definition of Pilz [11], [26]. The improved density prediction in the liquid phase in comparison with other EOS is shown in Supporting Information S3.1.

The α-function is a generalized temperature-dependent approach of Mathias, given in Schwartzentruber and Renon, with the polar parameters $p_{i}$, the reduced temperature $T_{r}=T/T_{c}$, and the acentric factor ω (see Table 2) [14], [15].

(22a) $$\begin{array}{cccc} \sqrt{\alpha } & =1+m\left(1-T_{r}^{0.5}\right)\\ & \;-p_{0}\left(1-T_{r}\right)\left(1+p_{1}\;T_{r}+p_{2}\;T_{r}^{2}\right) & & T_{r}\leq 1 \end{array}$$

(22b) $$\sqrt{\alpha }=\exp\left\{c_{d}\left(1-T_{r}^{d}\right)\right\}\;\;T_{r}>1$$

(22c) $$c_{d}=1-\frac{1}{d}$$

(22d) $$d=1+0.5\;m-p_{0}\left(1+p_{1}+p_{2}\right)$$

(22e) $$m=0.48508+1.55191\;\omega -0.15613\;\omega^{2}$$

The proposed VTBMSR-III varies in two aspects from the VTBMSR published by Pilz [11] (referred to as VTBMSR-II). The exponential term of α (Eq. (22b)) is extended by the exponent d based on the VTBMSR published by Pilz [26] (referred to as VTBMSR-I). Further, the criterion based on the reduced temperature for the selection of the α-function is interchanged compared to VTBMSR-II, taking Mathias [14] as reference who introduced the extension for $T_{r}>1$.

**Table 2:** Critical temperatures $T_{c}$, critical pressures $p_{c}$ and acentric factors ω for water, oxygen, nitrogen, and carbon dioxide, [1], [27].

		Water	Oxygen	Nitrogen	Carbon
					dioxide
$T_{c}$	[K]	647.14	154.58	126.20	304.12
$p_{c}$	[MPa]	22.064	5.043	3.398	7.374
ω	[-]	0.344	0.0222	0.037	0.225

The improved enthalpy and heat capacities predictions, especially in the liquid phase, in comparison with other EOS are shown in Supporting Information S3.2-S3.4.

The mixture parameters for the EOS of a mixture with N compounds are obtained by non-quadratic mixing rules given by Schwartzentruber and Renon [15].

(23) $$a_{\mathrm{mix}}=\sum_{i=1}^{N}\sum_{j=1}^{N}x_{i}x_{j}\sqrt{a_{i}a_{j}}\left[1-k_{a,\mathrm{ij}}-(x_{i}-x_{j})\;l_{\mathrm{ij}}\right]$$

(24) $$b_{\mathrm{mix}}=\sum_{i=1}^{N}\sum_{j=1}^{N}\frac{1}{2}x_{i}x_{j}(b_{i}+b_{j})\left(1-k_{b,\mathrm{ij}}\right)$$

The temperature-dependent binary interaction parameters $k_{a,\mathrm{ij}}$, $k_{b,\mathrm{ij}}$, and $l_{\mathrm{ij}}$ are given by

(25) $$k_{a,\mathrm{ij}}=k_{a,\mathrm{ij}}^{\left(0\right)}+k_{a,\mathrm{ij}}^{\left(1\right)}\;T+k_{a,\mathrm{ij}}^{\left(2\right)}/T$$

(26) $$k_{b,\mathrm{ij}}=k_{b,\mathrm{ij}}^{\left(0\right)}+k_{b,\mathrm{ij}}^{\left(1\right)}\;T+k_{b,\mathrm{ij}}^{\left(2\right)}/T$$

(27) $$l_{\mathrm{ij}}=l_{\mathrm{ij}}^{\left(0\right)}+l_{\mathrm{ij}}^{\left(1\right)}\;T+l_{\mathrm{ij}}^{\left(2\right)}/T$$

with $k_{a,\mathrm{ij}}=k_{a,\mathrm{ji}}$, $k_{b,\mathrm{ij}}=k_{b,\mathrm{ji}}$, $l_{\mathrm{ij}}=l_{\mathrm{ji}}$, $k_{a,\mathrm{ii}}=0$, $k_{b,\mathrm{ii}}=0$, and $l_{\mathrm{ii}}=0$.

For the volume translation an arithmetic-mean combining rule is required in order to preserve phase equilibria and properties of mixing [20]. Quadratic mixing rules combined with an arithmetic-mean combining rule result in a linear mixing rule.

(28) $$c_{\mathrm{mix}}=\sum_{i=1}^{N}x_{i}c_{i}$$

Since these mixing rules are composition-dependent they suffer from the Michelsen–Kistenmacher syndrome [28]. The Michelsen–Kistenmacher syndrome describes the effect of deviations resulting from the application of composition-dependent mixing rules. These rules are not invariant when a compound is divided into a number of identical subcompounds. Since such problems arise when mixtures contain similar compounds it is not only a theoretical issue. Another effect referred to as “dilution effect” occurs due to the double summation for the calculation of the $l_{\mathrm{ij}}$ term resulting in a product of three mole fractions. The impact of the syndrome is diminishing with increasing numbers of compounds. The EOS presented in this work avoids this effect by excluding the interaction parameter $l_{\mathrm{ij}}$, details are shown in the Supporting Information S1.4.

A further issue is isothermal crossing caused by applying a temperature-dependent volume translation. Lieball [17] and Privat et al. [20] mentioned observations of negative heat capacities at high temperatures and isotherm crossings in the pressure–volume plane when the volume translation is temperature-dependent. In our calculations, no negative heat capacities were observed. The behavior in the pressure–volume plane has not been studied.

The volume translation, polar and binary interaction parameters $c_{i}$, $p_{i}$, $k_{a,\mathrm{ij}}^{\left(z\right)}$, and $k_{b,\mathrm{ij}}^{\left(z\right)}$, are obtained by regression to experimental data as described in Sections 2.3 and 2.4.

### 2.2 Reference data/validation

Only few experimental data for mixtures of water, oxygen, nitrogen, and carbon dioxide of binary or higher order at supercritical conditions have been published. Japas and Franck published pvT data for $H_{2}O/N_{2}$ and $H_{2}O/O_{2}$ systems with varying temperature, pressure and concentration [29], [30]. They measured the molar volume at the three-dimensional (pTx) phase equilibrium. The covered range in temperature and pressure is 500–673 K (subcritical) and 20–270 MPa. The molar fraction of water varies from 0.134 to 0.9 in the $H_{2}O/N_{2}$ system and from 0.058 to 0.9 in the $H_{2}O/O_{2}$ system. Additionally, they published some data for a water–air system [30].

Gallagher et al. published experimental data for a $H_{2}O/N_{2}$ system as well as for a $H_{2}O/{\mathrm{CO}}_{2}$ system within a range of 400–1000 K, up to 100 MPa and molar fractions up to $x_{N_{2}}=0.8$ and $x_{{\mathrm{CO}}_{2}}=0.3$, respectively [31], [32]. Johns et al. measured the thermal conductivity of a binary mixture of nitrogen and carbon dioxide [33]. The obtained experimental data range covers temperatures from 302 to 470 K, molar fractions of nitrogen from 0.160 to 1 and pressures up to 26 MPa. For the pure substances water, oxygen, nitrogen, and carbon dioxide reference data of NIST (National Institute of Standards and Technology) using their software Refprop 9.1 is used for the validation of the improved EOS [27]. An overview of the reference data is shown in Table 3.

**Table 3:** Overview of reference data for pure substances and mixtures.

Reference	Substance(s)	Molar fraction	Temperature	Pressure	Properties
			[K]	[MPa]	
Refprop [27]	$H_{2}O$		292.15 – 882.15	0.1 – 35.1	T, p, ρ, v, h, $c_{v}$, $c_{p}$
	$O_{2}$		298.15 – 878.15	0.1 – 35.1	T, p, ρ, v, h, $c_{v}$, $c_{p}$
	$N_{2}$		298.15 – 878.15	0.1 – 35.1	T, p, ρ, v, h, $c_{v}$, $c_{p}$
	${\mathrm{CO}}_{2}$		298.15 – 878.15	0.1 – 35.1	T, p, ρ, v, h, $c_{v}$, $c_{p}$
Japas and Franck [29]	$H_{2}O/N_{2}$	0.14–0.9	480 – 660	11.5 – 270.5	T, p, v
Japas and Franck [30]	$H_{2}O/O_{2}$	0.08–0.9	470 – 660	19.5 – 280.0	T, p, v
Johns et al. [33]	$N_{2}/{\mathrm{CO}}_{2}$	0.16 – 0.708	320–470	9.0 – 30.8	T, p, ρ
Gallagher et al. [32]	$H_{2}O/{\mathrm{CO}}_{2}$	0.7 – 0.95	400 – 1000	0.05 – 100.0	T, p, v, h
Gallagher et al. [31]	$H_{2}O/N_{2}$	0.2 – 0.95	440 – 700	0.05 – 100.0	T, p, v, h
Japas and Franck [30]	$H_{2}O/\mathrm{air}$	0.791 – 0.798	620.5 – 673	27.0 – 279.5	T, p, v

### 2.3 Regression

The regression of the different parameters $c_{i}$, $p_{i}$, and $k_{b,\mathrm{ij}}$ on available experimental data was done using the software Aspen Plus^TM^, version 9.x and 10.1 from Aspen Technology Inc. and applying the maximum-likelihood method [11], [34]. Due to the research project of Pilz [11] on process modeling, Aspen has been considered as a suitable tool to determine parameters. The regression has been carried out with Aspen despite its limited applicability for the relevant parameters as mentioned below. Therefore, the current work was performed with Matlab 2017b (calculations) and R 3.4 (graphics). The used reference data is listed in Pilz [11] and in the Supporting Information S4.1.

As both the temperature and pressure were varied at the same time, the regression was unstable and prone to aborting before completion. Hence, the strategy was to regress first all three $c_{i}$ parameters using pvT data. Afterwards, the three polar parameters $p_{i}$ were determined using saturated pressure and caloric data. However, Aspen Plus^TM^ does not distinguish supercritical regions or phases but treats them as part of the vapor phase. For the regression of the density, all of its states, the liquid and the vapor/supercritical phase, and the saturated liquid and vapor state have to be taken into account in a single regression as the fugacity depends on this regression result. It follows that good representation of the density, mainly by the $c_{i}$ parameters, is decisive for the computation of the phase equilibria and the phase composition. In terms of the $p_{i}$ parameters, the data has to be split since the two temperature-dependent α-functions are valid either below or above the critical temperature. So, two data sets are retrieved for the $p_{i}$ parameters. The regression to oxygen of all three volume-translation $c_{i}$ parameters failed continuously. Instead, only one parameter, $c_{0}$, was used in the regression procedure. Nevertheless, the computation provides reasonable results. The implementation of the polar parameters $p_{i}$ worked properly. The same applies also to carbon dioxide. The regression of the binary parameters is based on the results of the pure substance regression. However, it was not possible to regress all binary interaction parameters due to convergence problems. Therefore, the focus lies on the determination of the $k_{b,\mathrm{ij}}$ parameters because they are included in both the repulsive and the attractive terms.

The resulting regressed parameter sets are listed in the Supporting Information S1.1.1–S1.1.3.

### 2.4 Parameters

For each pure compound and binary mixture different parameter sets are proposed. Thus, the best fitting set for each particular system has to be determined separately. Therefore, the reference data is recalculated with each parameter set. Based on the averaged absolute relative errors of the calculated data (see Table 4) the best fitting parameter set is selected, as listed in Table 5. Due to minimal differences in errors it is further considered that parameter sets are selected which are regressed to data of the same property (*e.g.* enthalpy or density). For water, this means that for the liquid and the vapor phase, the parameter sets resulting from the reference data of the same property (*e.g.* enthalpy) are selected. Concerning the mixtures, parameters sets determined by regression to density data are chosen for all binary mixtures.

**Table 4:** Averaged absolute relative errors and standard deviations [%] between calculated and reference data of pure compounds for the different parameter sets resulting from different reference data used for regression (in more detail in Supporting Information S1.1).

Substance	Set	$v_{m}$	h	$c_{v}$	$c_{p}$
${H_{2}O}_{\left(l\right)}$	1	6.6 ± 0.1	98 ± 3	11.7 ± 0.3	1.7 ± 0.1
	2	6.3 ± 0.1	94 ± 3	11.9 ± 0.3	1.8 ± 0.1
${H_{2}O}_{\left(v/\mathrm{sc}\right)}$	1	2.0 ± 0.1	0.82 ± 0.02	8.7 ± 0.3	6.3 ± 0.3
	2	2.3 ± 0.1	0.87 ± 0.03	10.7 ± 0.3	6.7 ± 0.3
	3	2.2 ± 0.1	0.85 ± 0.03	10.4 ± 0.3	6.7 ± 0.3
$O_{2}$	11	1.15 ± 0.02	0.37 ± 0.01	1.69 ± 0.02	0.83 ± 0.02
	12	0.463 ± 0.008	0.584 ± 0.008	2.78 ± 0.05	1.10 ± 0.03
	13	0.78 ± 0.01	0.353 ± 0.009	2.05 ± 0.03	0.89 ± 0.02
	14	0.543 ± 0.009	0.668 ± 0.009	2.92 ± 0.05	1.16 ± 0.03
	15	0.487 ± 0.009	0.611 ± 0.008	2.83 ± 0.05	1.12 ± 0.03
	21	0.418 ± 0.008	0.376 ± 0.006	1.69 ± 0.02	0.83 ± 0.02
	22	1.50 ± 0.03	0.99 ± 0.01	2.78 ± 0.05	1.10 ± 0.03
	23	0.78 ± 0.01	0.517 ± 0.007	2.05 ± 0.03	0.89 ± 0.02
	24	1.64 ± 0.03	1.10 ± 0.02	2.92 ± 0.05	1.16 ± 0.03
	25	1.55 ± 0.03	1.03 ± 0.01	2.83 ± 0.05	1.12 ± 0.03
$N_{2}$	1	0.326 ± 0.005	0.446 ± 0.006	1.97 ± 0.04	0.84 ± 0.02
	2	1.95 ± 0.03	0.97 ± 0.02	0.458 ± 0.008	0.552 ± 0.007
${\mathrm{CO}}_{2}$	1	3.07 ± 0.05	0.69 ± 0.01	4.9 ± 0.2	2.60 ± 0.08
	2	1.68 ± 0.03	0.483 ± 0.008	3.48 ± 0.07	2.11 ± 0.04

**Table 5:** Best-fit volume translation and polar parameters of the pure compounds. The subscripts (l) and (v/sc) indicate the liquid or vapor/supercritical phase of water. To avoid rounding errors the values are listed with full digits despite limited significance.

Parameter	Units	${H_{2}O}_{\left(l\right)}$	${H_{2}O}_{\left(v/\mathrm{sc}\right)}$	$O_{2}$	$N_{2}$	${\mathrm{CO}}_{2}$
Set		2	3	21	1	2
Reg. on		h	h	$c_{p}$	h	$c_{p}$
$c_{0}$	m^3^/mol	2.8126 × 10^−7^	2.8126 × 10^−7^	4.366 × 10^−6^	0	5.47 × 10^−6^
$c_{1}$	m^3^/mol	5.25308 × 10^−6^	5.25308 × 10^−6^	0	0	0
$c_{2}$	−	0.4054292	0.4054292	0	0	0
$p_{0}$	−	0.20914198	−1.92140347	0.07834762	0.067873	−1.55305545
$p_{1}$	−	−0.01398072	−1.1392853	−0.10036104	−0.015334	−1.52675479
$p_{2}$	−	0.07999497	0.22028766	−0.10036213	−0.015334	−0.51240405

**Fig. 1:**
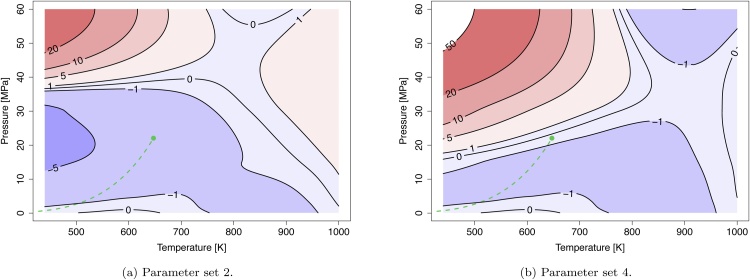
Isocontour plots of constant relative error [%] for the molar volume $\left[m^{3}/\mathrm{mol}\right]$ of the binary mixture $H_{2}O/N_{2}$ based on the reference data from Gallagher et al. [31]. The dashed green line and the green dot denote the vapor-liquid coexistence curve and the critical point of water, respectively.

For the determination of the best fitting binary interaction parameter set, the determined best fitting volume translation ($c_{i}$) and polar parameters ($p_{i}$) of the particular pure compounds are applied (Table 5). The resulting averaged absolute relative errors of the binary mixtures are listed in Table 6 and the selected parameters sets and theirs parameters are given in Table 7. Since the data range of Japas and Franck is mostly above the operating range of an SCWO process, for the binary mixture $H_{2}O/N_{2}$ the reference data of Gallagher et al. is weighted more due to the better representation of the process range [29], [30], [31]. In addition to the relative error, the error distribution is considered graphically, *e.g.* for $H_{2}O/N_{2}$ where the averaged relative errors differ only slightly for parameter sets 2 and 4. Fig. 1a and b show that parameter set 2 has the lower maximal error and a more even distribution within the observed range. Further it is considered that the regression is done on the same property data (*e.g.* density).

**Table 6:** Averaged absolute relative errors and standard deviations [%] between calculated and reference data of binary mixtures for the different interaction parameter sets resulting from different reference data used for regression (in more detail in Supporting Information S1.1).

Substance	Set	$v_{m}$	h
$H_{2}O/O_{2}$[30]	1	15 ± 1	
	2	3.0 ± 0.3	
	3	9.4 ± 0.6	
	4	8.8 ± 0.5	
	5	16.9 ± 0.7	
$H_{2}O/N_{2}$[29]	1	7.1 ± 0.6	
	2	6.6 ± 0.6	
	3	15 ± 1	
	4	2.6 ± 0.3	
$H_{2}O/N_{2}$[31]	1	2.4 ± 0.5	0.57 ± 0.05
	2	1.7 ± 0.4	0.25 ± 0.01
	3	3.4 ± 0.5	0.89 ± 0.01
	4	1.6 ± 0.4	0.34 ± 0.03
$H_{2}O/{\mathrm{CO}}_{2}$[32]	1	15 ± 4	3.7 ± 0.07
	2	0.7 ± 0.3	3.3 ± 0.06
$N_{2}/{\mathrm{CO}}_{2}$[33]	1	1.5 ± 0.2	
	2	1.1 ± 0.2

**Table 7:** Best-fit interaction parameters of the binary mixtures. To avoid rounding errors the values are listed with full digits despite limited significance.

Parameter	Unit	$H_{2}O/O_{2}$	$H_{2}O/N_{2}$	$H_{2}O/{\mathrm{CO}}_{2}$	${\mathrm{CO}}_{2}/N_{2}$	${\mathrm{CO}}_{2}/O_{2}$	$O_{2}/N_{2}$
Set		2	2	2	2		
Reg. to		ρ	ρ	ρ	ρ		
$k_{b,\mathrm{ij}}^{\left(0\right)}$	−	1.6786319	26.7175346	24.5882553	11.3800299	0	0
$k_{b,\mathrm{ij}}^{\left(1\right)}$	1/K	−0.00190476	−0.02120245	−0.0189643037	−0.0162626	0	0
$k_{b,\mathrm{ij}}^{\left(2\right)}$	K	−437.386995	−8387.43857	−7926.93286	−2008.99224	0	0

$k_{a,\mathrm{ij}}^{\left(z\right)}$ and $l_{\mathrm{ij}}^{\left(z\right)}$ are not used.

The values of volume translation, polar and binary interaction parameters for all sets are listed in Supporting Information S1.1.1 – S1.1.3 (Table S1.1 – S1.6).

### 2.5 Enthalpy, heat capacities and fugacity

Caloric properties such as enthalpy and heat capacities can be derived from a pressure-explicit equation of state. The general defining equations and the resulting equations for the VTBMSR-III EOS are given in Supporting Information S1.2.1 and S1.2.3, respectively.

## 3 Results and discussion

The prediction of any quantity ($x_{\mathrm{calc}}$) by the VTBMSR-III is discussed on the basis of the relative error which is defined as:

(29) $$e_{\mathrm{rel}}=\frac{x_{\mathrm{calc}}-x_{\mathrm{ref}}}{x_{\mathrm{ref}}}\times 100\;[\%].$$

where $x_{\mathrm{ref}}$ represents the value of the reference data. Negative relative errors represents an underestimation of the reference value. A positive relative error shows that the EOS overestimates the property.

For the comparison between different parameter sets or EOS, the averaged absolute relative error is defined as:

(30) $$\left|e_{\mathrm{rel}}\right|=\frac{1}{N}\sum_{i=1}^{N}\left|e_{\mathrm{rel},i}\right|.$$

First, the results for the pure compounds are discussed, followed by the four binary mixtures. Finally, calculations of the ternary mixture water-air are presented.

### 3.1 Pure substances

*Water*

**Fig. 2:**
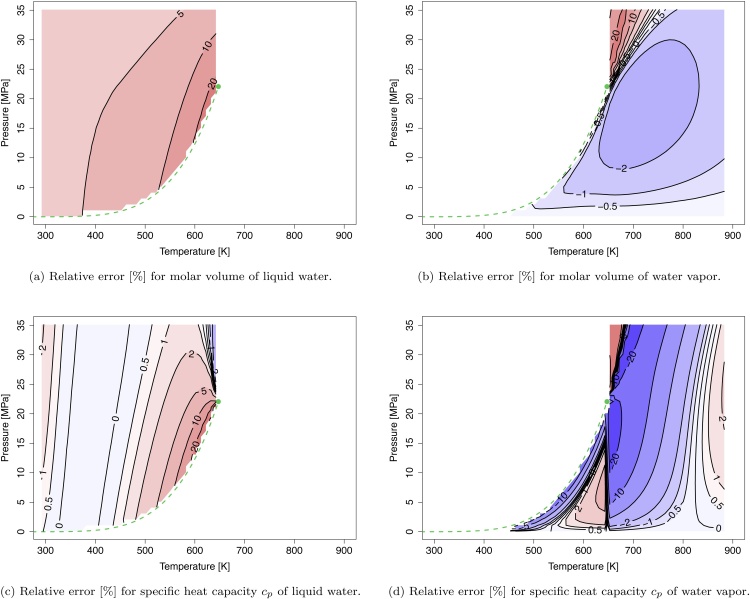
Isocontour plots of constant relative error [%] for the molar volume $\left[m^{3}/\mathrm{mol}\right]$ and the specific heat capacity at constant pressure [J/(mol K)] of substance water based on Refprop reference data [27]. The dashed green line and the green dot denote the vapor-liquid coexistence curve and the critical point of water, respectively.

Fig. 2 shows the relative error of the molar volume and the heat capacity at constant pressure of water calculated by the VTBMSR-III. In the liquid region the relative errors of the calculated molar volumes are around 5–10% increasing up to 20% near the vapor-liquid coexistence curve (Fig. 2a) and is consistently overestimated. A more accurate prediction is obtained for the vapor and supercritical phase (Fig. 2b) with relative errors of the molar volume around 2% except along the extended vapor-liquid coexistence curve in the supercritical region with relative errors up to 20%. Mostly, the molar volume is slightly underestimated in the vapor and supercritical phase. The vapor-liquid coexistence curve and its extension as ”pseudocritical line” above the critical point is the most difficult region to predict with relative errors between 10 and 20%. As for the molar volume, the vapor-liquid coexistence curve is the most problematic region to predict the heat capacity. Far off this curve the relative errors decrease below 5%. In the prediction of the heat capacity, the discontinuity at the critical temperature is remarkable. This phenomenon arises from the second derivative of parameter α which is not continuous across the critical temperature (see Supporting Information S1.3.3 and S3.5.5). The discontinuity of the second derivative of α is a known issue which affects cubic EOS [35], [36] (as discussed in the Supporting Information S3.5).

*Oxygen*

**Fig. 3:**
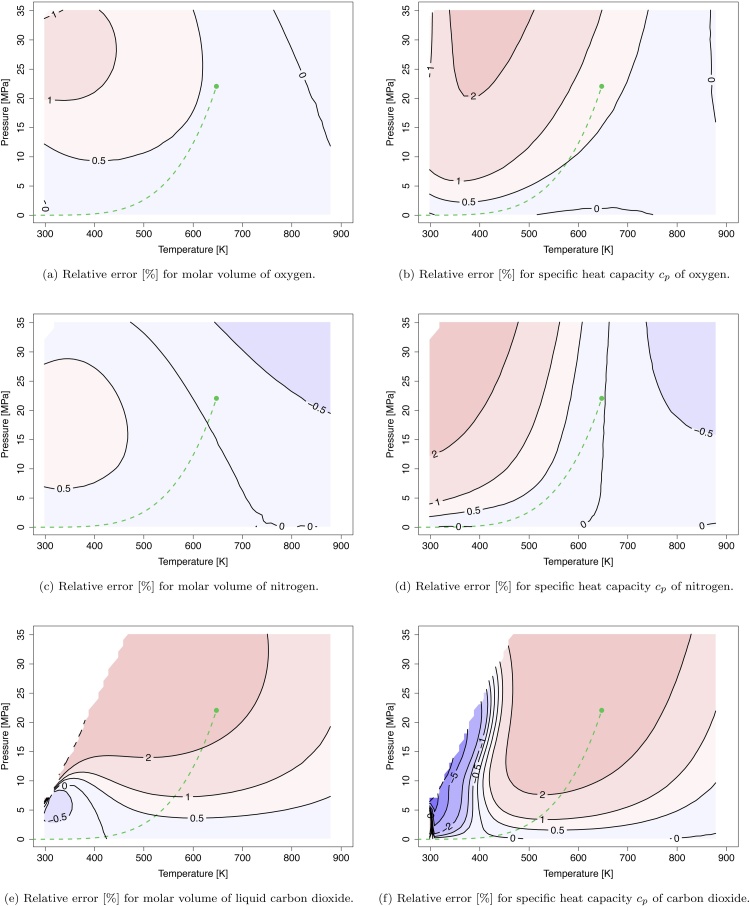
Isocontour plots of constant relative error [%] for the molar volume $\left[m^{3}/\mathrm{mol}\right]$ and the specific heat capacity at constant pressure [J/(mol K)] of oxygen, nitrogen, and carbon dioxide based on Refprop reference data [27]. The dashed green line and the green dot denote the vapor-liquid coexistence curve and the critical point of water, respectively.

The T−p space spanned in Fig. 3a and b is well above the critical temperature of oxygen ($T_{c}=154.58\;\;K$). At these conditions oxygen behaves almost like an ideal compressed gas. Therefore, the predictions of the VTBMSR-III in this region are in good agreement with the reference data. For the molar volume the relative errors are below 1%. The accuracy for the specific heat at constant pressure is within 2%. In contrast to the molar volume, which is slightly underestimated, the heat capacity is slightly overestimated. The excellent agreement with the reference data can be explained by the non-polar character of the oxygen molecule that is well represented by the EOS.

*Nitrogen*

Analogous to oxygen, the investigated T−p space is far away from the critical temperature of nitrogen ($T_{c}=126.20\;K$). Similar to oxygen, nitrogen consists of a simple, non-polar structure. This is recognizable in the low relative errors for the molar volume (<0.5%) and the heat capacity at constant pressure (<2%), shown in Fig. 3c and d.

*Carbon Dioxide*

Carbon dioxide is more challenging to predict due to its higher critical temperature ($T_{c}=304.12\;K$). The relative error of the molar volume is around 2% (Fig. 3e). The heat capacity at constant pressure has the highest deviation near the critical point of $CO_{2}\_(p_{c}=7.37MPa)$ and the extended vapor-liquid coexistence curve. Away from this curve, the relative error is around 2% similar to the molar volume. At the critical temperature $T_{c}=304.12\;\;\text{⁣}K$, the discontinuity in the heat capacity can be observed analogous to the phenomenon for water, see Fig. 3f.

*Comparison with other Cubic EOS*

The results of the VTBMSR-III are compared to other cubic EOS. An overview of the relative errors resulting from the different EOS is given in the Supporting Information S3.6.1. The results show that the VTBMSR-III predicts the thermodynamic properties with considerably higher accuracy, especially liquid water with an averaged absolute relative error of 6.3% compared to 20% and higher for other EOS.

### 3.2 Binary mixtures

The reference data for the binary mixtures do not contain any data for the heat capacities. For two binary mixtures, $H_{2}O/N_{2}$ and $H_{2}O/{\mathrm{CO}}_{2}$, Gallagher et al. published enthalpy values [31], [32]. The averaged absolute relative errors resulting from other EOS than VTBMSR-III are listed in Supporting Information S3.6.4.

*Water – Oxygen*

**Fig. 4:**
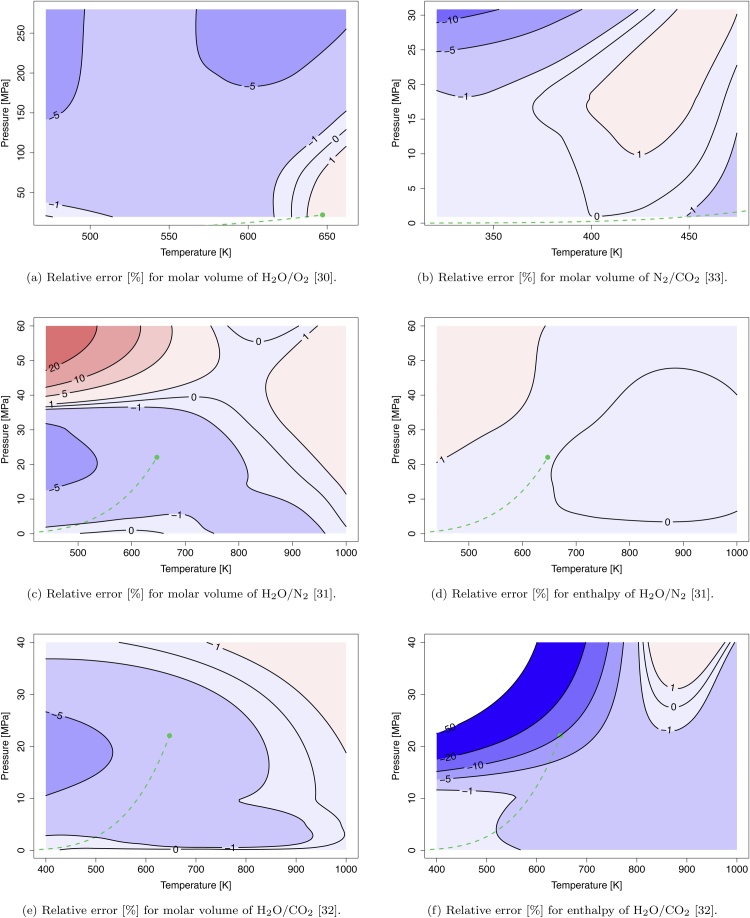
Isocontour plots of constant relative error [%] for the molar volume $\left[m^{3}/\mathrm{mol}\right]$ and the enthalpy [J/mol] of the binary mixtures $H_{2}O/O_{2}$, $H_{2}O/N_{2}$, $H_{2}O/{\mathrm{CO}}_{2}$, and $N_{2}/{\mathrm{CO}}_{2}$ based on their particular reference data [30], [31], [32], [33]. The dashed green line and the green dot denote the vapor-liquid coexistence curve and the critical point of water, respectively.

For the binary mixture $H_{2}O/O_{2}$ most of the reference data cover only a subcritical region (with respect to water) up to very high pressures which does not correspond to the operating points of an SCWO process [30]. Fig. 4a shows that in the main part of the range the molar volume is underestimated with relative errors up to 5%. Around the critical point the molar volume is slightly overestimated.

*Water – Nitrogen*

For the validation of the binary mixture $H_{2}O/N_{2}$, two data sets are available. Japas and Franck [29] cover a subcritical range with molar fractions of water between 0.14−0.9. At high pressures (p>100MPa) the relative error rises up to 20%. In the operating range of an SCWO process (p≤35  ⁣⁣ MPa), the relative error is lower (≤5%). Gallagher et al. [31] published data over a broad range of temperatures and pressures. To cover the operating range of an SCWO process, data in the range of T=440−1000  ⁣K, p=0.1−60  ⁣MPa and a composition of $x_{N_{2}}=0.05$ is considered. Within this range, the molar volume can be predicted with an accuracy of 5%, with exception of the low temperature/high pressure part with errors up to 20%, see Fig. 4c. Further, Gallagher et al. [31] published reference values for the enthalpy. These values can be recalculated accurately with relative errors around 1%, as shown in Fig. 4d.

*Water – Carbon Dioxide*

For the binary mixture $H_{2}O/{\mathrm{CO}}_{2}$, reference data of Gallagher et al. [32] is available covering the SCWO operating points (T=400−1000  ⁣K, p=0.2−40  ⁣MPa and a composition of $x_{{\mathrm{CO}}_{2}}=0.05$) and including data for the enthalpy. The molar volume of the $H_{2}O/{\mathrm{CO}}_{2}$ mixture can be predicted in the considered range with an accuracy of 5%, in the vapor/supercritical region even better (Fig. 4e). Fig. 4f shows the relative error of enthalpy which rises from 1% in the vapor/supercritical region up to 50% for low temperature and high pressure.

*Nitrogen – Carbon Dioxide*

The reference values of Johns et al. [33] cover only a narrow region of the SCWO process envelope due to the maximum temperature of 470 K, see Fig. 4b. Within the covered region the molar volume is accurately predicted with relative errors around 1%, except for low temperature and high pressure with up to 10% relative error.

### 3.3 Ternary mixture

*Water – Air*

Japas and Franck [30] published a few experimental data points for the ternary mixture water-air ($H_{2}O$, $O_{2}$, and $N_{2}$). The composition of air is not specified in Japas and Franck [30] and therefore it is assumed to be $x_{O_{2}}/x_{N_{2}}=1/4$. The resulting relative errors of the molar volume calculated by the VdW and VTBMSR-III are listed in Table 8. The relative error increases with pressure, for the VdW up to 60% at p=280 MPa and for the VTBMSR-III up to 15%. Apart from the more suitable prediction by the VTBMSR-III, the VdW overestimates the molar volume whereas the VTBMSR-III underestimates the molar volume. Considering data points with pressure below 35 MPa the relative error of the VTBMSR-III is less than 1%.

**Table 8:** Relative errors of the molar volume for a ternary mixture of water, oxygen, and nitrogen [30].

Molar fraction	Temperature	Pressure	Molar volume		
of $H_{2}O$			Reference	VdW	VTBMSR-III
	[K]	[MPa]	$\left[10^{-6}\;m^{3}/\mathrm{mol}\right]$	rel. error [%]	rel. error [%]
0.791	620.5	27.0	120.8	4.1	0.4
0.795	638.5	44.0	68.6	9.5	−0.6
0.797	640	68.3	46.7	26.6	−4.0
0.797	642	102.0	37.3	39.1	−6.8
0.798	645.5	152.9	31.8	48.2	−10.1
0.798	650	195.3	29.5	52.7	−12.3
0.798	652	214.8	28.7	54.4	−13.1
0.798	653.5	231.5	28.1	55.8	−13.7
0.798	657	263.9	27.2	57.8	−15.0
0.791	673	33.3	120.8	−0.9	−0.3
0.795	673	52.2	68.9	9.5	0.3
0.797	673	80.9	46.7	27.6	−2.6
0.797	673	118.6	37.4	40.1	−6.4
0.798	673	172.6	31.9	49.3	−10.7
0.798	673	214.2	29.5	54.2	−12.7
0.798	673	233.0	28.7	55.0	−13.5
0.798	673	249.3	28.1	57.3	−14.1
0.798	673	279.5	27.2	59.2	−15.4

## 4 Conclusion

The strength of the improved EOS is its simple structure with a low number of adjustable parameters enabling fast and stable computation, as well as the pressure-explicit formulation allowing to directly determine the desired thermodynamic properties of the compounds under SCWO conditions.

The molar volumes and heat capacities of oxygen and nitrogen are predicted very accurately with relative errors below 2%. Water and carbon dioxide are more difficult to predict in the considered temperature and pressure range including their critical points and vapor–liquid coexistence curves. For pure water, the prediction of the molar volume and of the specific heat capacity is more accurate far away from the vapor-liquid coexistence curve. The relative error is highest in a narrow region along the vapor–liquid coexistence curve towards near-critical conditions. With relative errors mostly below 10%, the predictions of liquid water show higher accuracy than the ones of conventional EOS. The same improvement is observed for the prediction of the binary mixtures $H_{2}O/O_{2}$, $H_{2}O/N_{2}$, $H_{2}O/{\mathrm{CO}}_{2}$, and $N_{2}/{\mathrm{CO}}_{2}$, as well as for the ternary mixture $H_{2}O/O_{2}/N_{2}$ with relative errors below 10% within the SCWO process range. Therefore, the improved EOS is a powerful tool to model and design an SCWO process.

While the data for binary mixtures including water cover a wide range of conditions, only a few data points are available for ternary mixtures. This lack of data for higher mixtures seriously hinders the further validation and refinement of EOS for SCWO and other applications.
