# Supplementary material for: Equation of state and thermodynamic properties for mixtures of H2O, O2, N2, and CO2 from ambient up to 1000 K and 280 MPa
Source: J Supercrit Fluids. 2019 Nov;153:104476. doi: 10.1016/j.supflu.2019.02.016 (PMC6919335; doi:10.1016/j.supflu.2019.02.016)
Supplement: Supplementary file 1 [file mmc1.pdf]

Equation of State and Thermodynamic Properties for Mixtures of H_2O, O_2, N_2, and CO_2 from Ambient up to 1000 K and 280 MPa

F. Mangold[a], St. Pilz[b,*], S. Bjelić[c], F. Vogel[a,c,**]

[a] *University of Applied Sciences and Arts Northwestern Switzerland FHNW, School of Engineering, Klosterzelgstrasse 2, 5210 Windisch, Switzerland*
[b] *by then DaimlerChrysler, Research and Technology (FT4/TP), 89013 Ulm, Germany*
[c] *Paul Scherrer Institute PSI, 5232 Villigen PSI, Switzerland*

S. Supporting Information

S1 VTBMSR-III **2**
S1.1 Parameters c_i, p_i and $k_{b,ij}^{(z)}$ . . . 2
S1.1.1 Volume Translation Parameters c_i . . . 2
S1.1.2 Polar Parameters p_i . . . 2
S1.1.3 Binary Interaction Parameters $k_{b,ij}^{(z)}$ . . . 3
S1.2 Thermodynamic Properties . . . 5
S1.2.1 Definitions . . . 5
S1.2.2 Volume Translation Corrected Equation for Fugacity by Kutney . . . 6
S1.2.3 Derived Properties from VTBMSR-III . 7
S1.3 Derivatives of $\alpha(T)$ and $c(T)$ . . . 7
S1.3.1 Parameter α . . . 7
S1.3.2 Parameter c . . . 9
S1.3.3 Discussion of Derivatives of α and c . . 10
S1.4 Michelsen-Kistenmacher Syndrome . . . 12
S1.4.1 Theoretical Approach . . . 12
S1.4.2 Dilution Effect . . . 14
S1.4.3 Pseudo Quaternary System of Water . . 15
S1.4.4 Pseudo Quaternary System of H_2O/N_2 15

S2 Other Equations of State **16**
S2.1 Redlich-Kwong . . . 16
S2.1.1 Parameter α and its Derivatives . . . 16
S2.2 Redlich-Kwong-Soave . . . 17
S2.2.1 Parameter α and its Derivatives . . . 18
S2.3 Peng-Robinson . . . 19
S2.3.1 Parameter α and its Derivatives . . . 20
S2.4 VTBMSR-I . . . 21
S2.4.1 Parameter α and its Derivatives . . . 22
S2.4.2 Parameter c and its Derivatives . . . 23
S2.5 VTBMSR-II . . . 24
S2.5.1 Parameter α and its Derivatives . . . 25
S2.5.2 Parameter c and its Derivatives . . . 26

S3 Comparison of Different EOS **27**
S3.1 Density Prediction along Vapor-Liquid Coexistence Curve of Water . . . 27
S3.2 Enthalpy Prediction along Vapor-Liquid Coexistence Curve of Water . . . 27
S3.3 Heat Capacity at Constant Volume Prediction along Vapor-Liquid Coexistence Curve of Water 28
S3.4 Heat Capacity at Constant Pressure Prediction along Vapor-Liquid Coexistence Curve of Water 28
S3.5 Derivatives of Different α-functions . . . 29
S3.5.1 Boston and Mathias . . . 29
S3.5.2 Mathias . . . 30
S3.5.3 VTBMSR-I . . . 31
S3.5.4 VTBMSR-II . . . 32
S3.5.5 VTBMSR-III . . . 33
S3.5.6 Summary . . . 34
S3.5.7 Graphics . . . 34
S3.6 Error Distributions . . . 38
S3.6.1 Averaged Absolute Relative Errors for Pure Substances for Different Equations of State . . . 38
S3.6.2 Relative Error in the Prediction of the Molar Volume of Liquid Water . . . 42
S3.6.3 Relative Error in the Prediction of the Molar Volume of Water Vapor . . . 44
S3.6.4 Averaged Absolute Relative Errors for Binary Mixtures for Different Equations of State . . . 46
S3.6.5 Relative Error in the Prediction of the Molar Volume of H_2O/O_2 mixture . . . 47

S4 Regression **49**
S4.1 Database for regression . . . 49
S4.1.1 Pure compounds . . . 49
S4.1.2 Binary mixtures . . . 49

*Current address: stephan.pilz@t-online.de
**Corresponding author. Tel.: +41 56 202 73 34.
Email address: frederic.vogel@fhnw.ch (F. Vogel)

S1. VTBMSR-III

S1.1. Parameters c_i, p_i and $k_{b,ij}^{(z)}$

In the subsequent sections, all regressed parameter sets found by regression (see Section 2.3) for the volume translation parameters c_i, the polar parameters p_i and the binary interaction parameters $k_{b,ij}^{(z)}$ are listed. For oxygen, the parameter set numbering is given by a two-digit number due to two different parameter sets for the volume translation parameters. The first digit indicates the parameter set for the volume translation, the second digit the one for the polar parameters (indicated with placeholder x). As an example parameter set 21 for oxygen: The volume translation parameter c_i are taken from parameter set 2 (regarding volume translation) obtained by regression on density data of the liquid phase of Sychev and IUPAC [B, C]. The polar parameters p_i are taken from parameter set 1 (regarding polarity) obtained by regression on heat capacity data of the vapor and supercritical phase of Sychev [B]. The parameter sets of water, nitrogen and carbon dioxide are given by one digit since only one reference data set was applied for determination of the volume translation parameters c_i. Therefore, the digit has only to indicate the parameter set for the polar parameters p_i.

S1.1.1. Volume Translation Parameters c_i

Table S1.1: Volume translation parameters for water, oxygen, nitrogen, and carbon dioxide. To avoid rounding errors the values are listed with full digits despite limited significance. In the column "Regressed on" the considered phase is specified along with the property: l (liquid), v (vapor), sc (supercritical). References see Section S4.1.

Substance	Set	c_0 m^3/mol	c_1 m^3/mol	c_2 [−]	Regressed on	Reference
H_2O		2.8126×10^{-7}	$5.253\,08 \times 10^{-6}$	0.405 429 2	Density data (l+v+sc)	[A]
O_2	1x	4.366×10^{-6}	0	0	unknown	unknown
	2x	2.23×10^{-7}	0	0	Density data (l)	[B, C]
N_2		0	0	0	-	[D]
CO_2		5.47×10^{-6}	0	0	Density data (l+v+sc)	[E]

S1.1.2. Polar Parameters p_i

Table S1.2: Polar parameters for water, oxygen, nitrogen, and carbon dioxide. The subscripts (l) and (v/sc) indicate the liquid or vapor/supercritical phase of water. To avoid rounding errors the values are listed with full digits despite limited significance. In the column "Regressed on" the considered phase is specified along with the property: l (liquid), v (vapor), sc (supercritical). References see Section S4.1.

Substance	Set	p_0 [−]	p_1 [−]	p_2 [−]	Regressed on	Reference
$H_2O_{(l)}$	1	0.215 497 72	−0.011 736 3	0.089 201 52	Heat capacity data (l)	[A]
	2	0.209 141 98	−0.013 980 72	0.079 994 97	Enthalpy data (l)	[A]
$H_2O_{(v)}$	1	−4.131 460 8	−1.709 664 99	0.764 512 84	Heat capacity data (v)	[A]
	2	−4.896 311 76	−1.722 511 78	0.752 615 93	Heat capacity data (sc)	[A]
	3	−1.921 403 47	−1.139 285 3	0.220 287 66	Enthalpy data (v+sc)	[A]
O_2	x1	0.078 347 62	−0.100 361 04	−0.100 362 13	Heat capacity data (v+sc)	[B]
	x2	−0.514 489 63	−1.381 640 36	0.391 668 94	Heat capacity data (v+sc)	[C]
	x3	0.058 806 46	−0.144 563 07	−0.139 299 52	Enthalpy data (v+sc)	[B]
	x4	0.001 818 16	30.381 771	−40.195 459 6	Enthalpy (l)	[C]
	x5	−0.154 704 86	−0.010 508 47	−0.932 976 16	Enthalpy (v+sc)	[C]
N_2	1	0.067 873	−0.015 334	−0.015 334	Enthalpy data	[D]
	2	−0.182 101	−0.055 424	−0.055 423	Heat capacity data	[D]
CO_2	1	−20.916 858 5	−1.835 000 97	0.837 382 87	Enthalpy data (sc)	[E]
	2	−1.553 055 45	−1.526 754 79	0.512 404 05	Heat capacity data (sc)	[E]

S1.1.3. Binary Interaction Parameters $k_{b,ij}^{(z)}$

Table S1.3: Interaction parameters for H_2O/O_2 mixture. To avoid rounding errors the values are listed with full digits despite limited significance.

Set	Interaction parameter		Value	Composition x_{O_2}	Regressed on	Reference
1	$k_{b,0}$	[−]	−11.262 726	0.05	Enthalpy data	[30]
	$k_{b,1}$	[1/K]	0.013 301 72			
	$k_{b,2}$	[K]	1980.228 46			
2	$k_{b,0}$	[−]	1.678 631 9	0.05	Density data	[30]
	$k_{b,1}$	[1/K]	−0.001 904 76			
	$k_{b,2}$	[K]	−437.386 995			
3	$k_{b,0}$	[−]	−30.604 478 1	0.2	Enthalpy data	[30]
	$k_{b,1}$	[1/K]	0.025 743 48			
	$k_{b,2}$	[K]	9074.717			
4	$k_{b,0}$	[−]	26.717 534 6	0.2	Density data	[30]
	$k_{b,1}$	[1/K]	−0.021 202 45			
	$k_{b,2}$	[K]	−8387.438 57			
5	$k_{b,0}$	[−]	−3.734 421 9	0.1 − 0.2	Specific volume data	[30]
	$k_{b,1}$	[1/K]	0.010 657 38			
	$k_{b,2}$	[K]	−2509.316 19			

Table S1.4: Interaction parameters for H_2O/N_2 mixture. To avoid rounding errors the values are listed with full digits despite limited significance.

Set	Interaction parameter		Value	Composition x_{N_2}	Regressed on	Reference
1	$k_{b,0}$	[−]	−30.604 478 1	0.05	Enthalpy data	[29, 31]
	$k_{b,1}$	[1/K]	0.025 743 48			
	$k_{b,2}$	[K]	9074.717			
2	$k_{b,0}$	[−]	26.717 534 6	0.05	Density data	[29, 31]
	$k_{b,1}$	[1/K]	−0.021 202 45			
	$k_{b,2}$	[K]	−8387.438 57			
3	$k_{b,0}$	[−]	−11.262 767	0.2	Enthalpy data	[29, 31]
	$k_{b,1}$	[1/K]	0.013 301 72			
	$k_{b,2}$	[K]	1980.228 46			
4	$k_{b,0}$	[−]	1.678 631 9	0.2	Density data	[29, 31]
	$k_{b,1}$	[1/K]	−0.001 904 76			
	$k_{b,2}$	[K]	−437.386 995			

Table S1.5: Interaction parameters for H_2O/CO_2 mixture. To avoid rounding errors the values are listed with full digits despite limited significance.

Set	Interaction parameter		Value	Composition x_{CO_2}	Regressed on	Reference
	$k_{b,0}$	[−]	−33.561 599 3			
1	$k_{b,1}$	[1/K]	0.028 380 74	0.05	Enthalpy data	[32]
	$k_{b,2}$	[K]	1000			
	$k_{b,0}$	[−]	24.588 255 3			
2	$k_{b,1}$	[1/K]	−0.018 964 303 7	0.05	Density data	[32]
	$k_{b,2}$	[K]	−7926.932 86			

Table S1.6: Interaction parameters for N_2/CO_2 mixture. To avoid rounding errors the values are listed with full digits despite limited significance.

Set	Interaction parameter		Value	Composition x_{N_2}	Regressed on	Reference
	$k_{b,0}$	[−]	0.176 891 66			
1	$k_{b,1}$	[1/K]	−0.001 266 22	$0.16 - 0.71$	Heat capacity data	[33]
	$k_{b,2}$	[K]	42.946 91			
	$k_{b,0}$	[−]	11.380 029 9			
2	$k_{b,1}$	[1/K]	−0.016 262 6	$0.16 - 0.71$	Density data	[33]
	$k_{b,2}$	[K]	−2008.992 24			

S1.2. Thermodynamic Properties

S1.2.1. Definitions

Enthalpy.

For a non-ideal gas the enthalpy (h) can be calculated as a difference of the ideal enthalpy (h_{ig}) and the residual enthalpy (h_{res}).

$$h = h_{ig} - h_{res} \tag{S1.1}$$

The relation for the ideal enthalpy is independently of the EOS and given in Pilz [11]:

$$h_{ig}(T,p) = h_0 + \int_{T_0}^{T} c_p^0(T)\, dT \tag{S1.2}$$

the one for the residual enthalpy in Lieball [17]:

$$h_{res} = RT - pv + \int_{\infty}^{v} \left[p - T \left(\frac{\partial p}{\partial T} \right)_v \right] dv. \tag{S1.3}$$

The ideal specific heat capacity at constant pressure (c_p^0) is determined based on a polynomial approach [35].

$$\frac{c_p^0}{R} = \alpha + \beta\, T + \gamma\, T^2 + \delta\, T^3 + \varepsilon\, T^4 \tag{S1.4}$$

The parameters α, β, γ, δ and ε for the particular substances are listed in Table S1.7 for completeness.

Table S1.7: Constants for the calculation of the ideal heat capacity at constant pressure [35].

	α [−]	$\beta \cdot 10^3$ [1/K]	$\gamma \cdot 10^6$ [1/K^2]	$\delta \cdot 10^9$ [1/K^3]	$\varepsilon \cdot 10^{12}$ [1/K^4]
H_2O	4.070	−1.108	4.152	−2.964	0.807
O_2	3.626	−1.878	7.055	−6.764	2.156
N_2	3.675	−1.208	2.324	−0.632	−0.226
CO_2	2.401	−1.619	3.692	−2.032	0.240

Heat Capacity at Constant Volume.

The specific heat capacity at constant volume (c_v) can be calculated using its definition and the definition of the enthalpy due to the pressure-explicit equation of state [35].

$$c_v = \left.\frac{\partial u}{\partial T}\right|_v = \left.\frac{\partial h}{\partial T}\right|_v - \left.\frac{\partial pv}{\partial T}\right|_v \tag{S1.5}$$

Heat Capacity at Constant Pressure.

The specific heat capacity at constant pressure (c_p) is defined as [35]:

$$c_p = \left.\frac{\partial h}{\partial T}\right|_p . \tag{S1.6}$$

Since the equation of state is pressure-explicit the definition of the heat capacity at constant pressure cannot be used directly. Instead, it can be related to the heat capacity at constant volume by the subsequent relation [36].

$$c_p - c_v = -T \frac{\left(\frac{\partial p}{\partial T}\right)_v^2}{\left(\frac{\partial p}{\partial v}\right)_T} \tag{S1.7}$$

Fugacity.

The fugacity (f) can be described as the product of the pressure and the fugacity coefficient:

$$f = \phi \cdot p \tag{S1.8}$$

where the fugacity coefficient is calculated by [36]:

$$\ln \phi = \frac{pv}{RT} - 1 - \ln\left(\frac{pv}{RT}\right) - \frac{1}{RT} \int_{\infty}^{v} \left(p - \frac{RT}{\tilde{v}} \right) d\tilde{v}. \tag{S1.9}$$

S1.2.2. Volume Translation Corrected Equation for Fugacity by Kutney

Kutney [18] proposed corrected equations for derived thermodynamic properties when a volume translation is used.

$$\ln \phi_i = -\int_{\infty}^{V^{UT}} \left[\frac{p}{RT} - \frac{1}{V}\right] \left(\frac{\partial V}{\partial V^{UT}}\right)_T dV^{UT} - \ln\frac{pV}{RT} + \frac{pV}{RT} - 1 \tag{S1.10}$$

$$h_{res} = pV - RT - \int_{\infty}^{V^{UT}} \left[p - T\left(\frac{\partial p}{\partial T}\right)_V\right] \left(\frac{\partial V}{\partial V^{UT}}\right)_T dV^{UT} \tag{S1.11}$$

In general, the volume corrected integral is

$$\int_{\infty}^{V^{UT}} f(p,T) \left(\frac{\partial V}{\partial V^{UT}}\right)_T dV^{UT} \tag{S1.12}$$

corresponding to

$$\int_{x_a}^{x_b} f(p,T) \left(\frac{\partial V}{\partial V^{UT}}\right)_T dx \tag{S1.13}$$

which can be substituted to

$$\int_{y_a}^{y_b} f(p,T) \left(\frac{\partial V}{\partial V^{UT}}\right)_T \cdot \left(\frac{dx}{dy}\right) dy \tag{S1.14}$$

with $x = g(y)$, $y = g^{-1}(x)$, and $\frac{dx}{dx} = g'(y)$. In our case, it is

$$x_a = \infty \tag{S1.15}$$

$$x_b = V^{UT} \tag{S1.16}$$

$$dx = dV^{UT}, \tag{S1.17}$$

and dy should become to dV. The relation between the untranslated V^{UT} and translated V volume is given by

$$V^{UT} = V - c \tag{S1.18}$$

$$x = y - c. \tag{S1.19}$$

If follows,

$$g(y) = y - c \quad (= x) \tag{S1.20}$$

$$g^{-1}(x) = x + c \quad (= y) \tag{S1.21}$$

and

$$y_a = g^{-1}(x_a) = \infty + c = \infty \tag{S1.22}$$

$$y_b = g^{-1}(x_b) = V^{UT} + c = V \tag{S1.23}$$

$$dy = dV \tag{S1.24}$$

$$\frac{dx}{dy} = \left(\frac{\partial V^{UT}}{\partial V}\right)_T. \tag{S1.25}$$

Insert these results into Eq. S1.14, it follows

$$\int_{y_a}^{y_b} f(p,T) \left(\frac{\partial V}{\partial V^{UT}}\right)_T \cdot \left(\frac{dx}{dy}\right) dy = \int_{\infty}^{V} f(p,T) \left(\frac{\partial V}{\partial V^{UT}}\right)_T \cdot \left(\frac{\partial V^{UT}}{\partial V}\right)_T dV. \tag{S1.26}$$

and with

$$\left(\frac{\partial V}{\partial V^{UT}}\right)_T = \left(\frac{\partial (V^{UT} + c(T))}{\partial V^{UT}}\right)_T = 1 + 0 = 1 \tag{S1.27}$$

$$\left(\frac{\partial V^{UT}}{\partial V}\right)_T = \left(\frac{\partial (V - c(T))}{\partial V}\right)_T = 1 + 0 = 1 \tag{S1.28}$$

ending in

$$\int_{\infty}^{V} f(p,T)\, dV. \tag{S1.29}$$

Therefore, the volume corrected equations of Kutney [18] (Eq. (S1.10) and (S1.11)) are equal to our equations for the fugacity and enthalpy, see Eqs. (S1.9) and (S1.3), respectively.

S1.2.3. Derived Properties from VTBMSR-III

Residual enthalpy

$$h_{res} = RT - v\left(\frac{RT}{v+c-b} - \frac{a_c\,\alpha}{(v+c)(v+c+b)}\right) + \frac{a_c(\alpha' T - \alpha)}{b}\ln\left|\frac{v+c}{v+c+b}\right| - \frac{RT^2c'}{v+c-b} + \frac{a_c\,\alpha c' T}{(v+c)(v+c+b)} \tag{S1.30}$$

Ideal enthalpy

$$h_{ig} = h_0 + \int_{T_0}^{T} c_p^0(T)\,dT \tag{S1.31}$$

Enthalpy

$$h = h_0 + \int_{T_0}^{T} c_p^0(T)\,dT - RT + \frac{RTv}{v+c-b} - \frac{a_c\,\alpha v}{(v+c)(v+c+b)} - \frac{a_c(\alpha' T - \alpha)}{b}\ln\left|\frac{v+c}{v+c+b}\right| + \frac{RT^2c'}{v+c-b} - \frac{a_c\,\alpha c' T}{(v+c)(v+c+b)} \tag{S1.32}$$

Heat capacity at constant volume

$$c_v = c_p^0(T) - R - \frac{a_c\,\alpha'' T}{b}\ln\left|\frac{v+c}{v+c+b}\right| + \frac{2RTc' + RT^2c''}{v+c-b} - \frac{R(c'T)^2}{(v+c-b)^2} - \frac{2a_c\,\alpha' c' T + a_c\,\alpha c'' T}{(v+c)(v+c+b)} + \frac{a_c\,\alpha (c')^2 T(2(V+c)+b)}{(v+c)^2(v+c+b)^2} \tag{S1.33}$$

Heat capacity at constant pressure

$$c_p = c_v - T\,\frac{\left(\frac{\partial p}{\partial T}\right)_v^2}{\left(\frac{\partial p}{\partial v}\right)_T} \tag{S1.34}$$

with

$$\left.\frac{\partial p}{\partial T}\right|_v = \frac{R}{v+c-b} - \frac{RTc'}{(v+c-b)^2} - \frac{a_c\,\alpha'}{(v+c)(v+c+b)} + \frac{a_c\,\alpha c'(2(v+c)+b)}{(v+c)^2(v+c+b)^2} \tag{S1.35}$$

and

$$\left.\frac{\partial p}{\partial v}\right|_T = -\frac{RT}{(v+c-b)^2} + a_c\,\alpha\left(\frac{2(v+c)+b}{(v+c)^2(v+c+b)^2}\right) \tag{S1.36}$$

Fugacity coefficient

$$\ln\phi = -\ln\left|\frac{v+c-b}{v}\right| + \frac{a_c\,\alpha}{bRT}\ln\left|\frac{v+c}{v+c+b}\right| \tag{S1.37}$$

S1.3. Derivatives of $\alpha(T)$ and $c(T)$

S1.3.1. Parameter α

- α

$$\alpha = \begin{cases} \left[1 + m(1-\sqrt{T_r}) - p_0(1-T_r)(1+p_1T_r+p_2T_r^2)\right]^2 & T_r \le 1 \\ \left[\exp\left\{c_d(1-T_r^d)\right\}\right]^2 & T_r > 1 \end{cases} \tag{S1.38}$$

- α'
  - $T_r \le 1$

$$\alpha' = 2\cdot\left[1 + m(1-\sqrt{T_r}) - p_0(1-T_r)(1+p_1T_r+p_2T_r^2)\right] \cdot\left[-\frac{m}{2\,T_c\sqrt{\frac{T}{T_c}}} + \frac{3\,p_0p_2T^2}{T_c^3} + 2\,p_0\,(p_1-p_2)\,\frac{T}{T_c^2} + p_0\,(1-p_1)\,\frac{1}{T_c}\right] \tag{S1.39}$$

- $T_r > 1$

$$\alpha' = -2\,c_d d \frac{1}{T_c}\left(\frac{T}{T_c}\right)^{d-1} \exp\left[2\,c_d\left(1-\left(\frac{T}{T_c}\right)^{d}\right)\right] \tag{S1.40}$$

- α''
  - $T_r \leq 1$

$$\begin{aligned}\alpha'' = 2\cdot &\left[1+m(1-\sqrt{T_r})-p_0(1-T_r)(1+p_1T_r+p_2T_r^2)\right]\\ &\cdot\left[\frac{m}{4\,T_c^2\left(\frac{T}{T_c}\right)^{3/2}}+2\,p_0\,(p_1-p_2)\,\frac{1}{T_c^2}+6\,p_0p_2\left(\frac{T}{T_c^3}\right)\right]\\ &+2\cdot\left[-\frac{m}{2\,T_c\sqrt{\frac{T}{T_c}}}+\frac{3\,p_0p_2T^2}{T_c^3}+2\,p_0\,(p_1-p_2)\,\frac{T}{T_c^2}+p_0\,(1-p_1)\,\frac{1}{T_c}\right]^2\end{aligned} \tag{S1.41}$$

  - $T_r > 1$

$$\alpha'' = -2\,c_d d\,(d-1)\,\frac{1}{T_c^2}\left(\frac{T}{T_c}\right)^{d-2} \exp\left[2\,c_d\left(1-\left(\frac{T}{T_c}\right)^{d}\right)\right] \tag{S1.42}$$

$$+\;4\,c_d^2 d^2\frac{1}{T_c^2}\left(\frac{T}{T_c}\right)^{2(d-1)} \exp\left[2\,c_d\left(1-\left(\frac{T}{T_c}\right)^{d}\right)\right] \tag{S1.43}$$

S1.3.2. Parameter c

- c

$$c = \begin{cases} c_0 + \frac{c_1}{1+c_2-T_r} & T_r \leq 1 \\ c_0 & T_r \leq 1, c_2 = T_r - 1 \\ c_0 & T_r > 1, c_1 = 0 \\ b + \frac{\left(\frac{(c_0-b)c_2}{c_1}+1\right)^2 c_1}{1+c_2\left(\frac{(c_0-b)c_2}{c_1}+1\right)-T_r} & T_r > 1, c_1 \neq 0 \\ c_0 & T_r > 1, c_1 \neq 0, c_2\left(\frac{(c_0-b)c_2}{c_1}+1\right) = T_r - 1 \end{cases} \tag{S1.44}$$

- c'
  - $T_r \leq 1$

$$c' = \frac{c_1}{\left(1 + c_2 - \frac{T}{T_c}\right)^2 T_c} \tag{S1.45}$$

  - $T_r > 1$

$$c' = \frac{\left(\frac{(c_0-b)c_2}{c_1}+1\right)^2 c_1}{\left(1 + c_2\left(\frac{(c_0-b)c_2}{c_1}+1\right) - \frac{T}{T_c}\right)^2 T_c} \tag{S1.46}$$

- c''
  - $T_r \leq 1$

$$c'' = \frac{2\,c_1}{\left(1 + c_2 - \frac{T}{T_c}\right)^3 T_c^2} \tag{S1.47}$$

  - $T_r > 1$

$$c'' = \frac{2\left(\frac{(c_0-b)c_2}{c_1}+1\right)^2 c_1}{\left(1 + c_2\left(\frac{(c_0-b)c_2}{c_1}+1\right) - \frac{T}{T_c}\right)^3 T_c^2} \tag{S1.48}$$

S1.3.3. Discussion of Derivatives of α and c

In the result section, it is mentioned that the second derivative of parameter α causes discontinuities in the calculation of the heat capacities c_v and c_p. In Figure S1.1, the temperature dependencies of α, c and their derivatives are illustrated within the range of an SCWO process (200 - 800 K). The limit values of the α-function and its derivatives at $T_r = 1$ are determined in Section S3.5.5.

Figures S1.1a and S1.1b show that the parameters α and c are continuous in the considered temperature range for all of the four substances water, oxygen, nitrogen, and carbon dioxide. The same applies to the first derivatives of both parameters, see Figure S1.1c and S1.1d. But the second derivatives are no longer continuous for all of the four substances, see Figure S1.1e and S1.1f. The second derivatives of water and carbon dioxide are disrupt at their critical temperatures, $T_{c,H_2O} = 647.14$ K and $T_{c,CO_2} = 304.12$ K, respectively. Since the critical temperatures of oxygen and nitrogen, $T_{c,O_2} = 154.58$ K and $T_{c,N_2} = 126.20$ K, are lower than the considered temperature range, the second derivatives of α for oxygen and nitrogen are continuous. Compared to the second derivative of α, only the second derivative of c of water is discontinuous. Since the parameters c_1 and c_2 are 0 for oxygen, nitrogen, and carbon dioxide, also c'' of carbon dioxide is continuous. Therefore, it is identifiable that the second derivative of parameter α causes the discontinuities in the heat capacities.

This discontinuity of the second derivative of the parameter α is a known issue. Le Guennec *et al.* [37] have developed requirements for the α function so that vapor-liquid equilibrium and derived thermodynamic properties can be properly predicted at all temperatures. No common α-function fulfills the proposed constraints without any restrictions on the adjustable parameters.

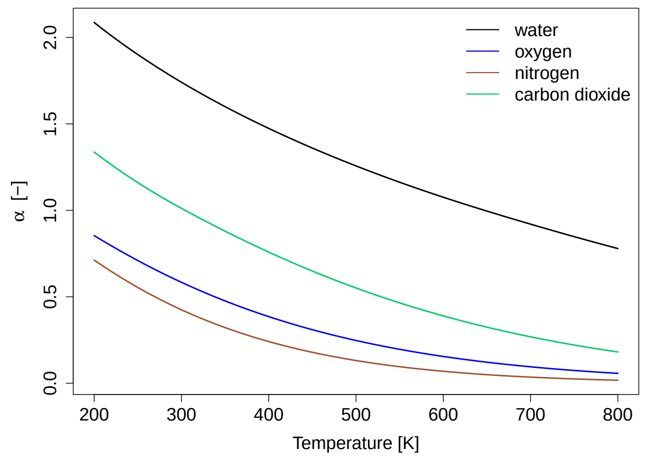

(a) Parameter $\alpha(T)$.

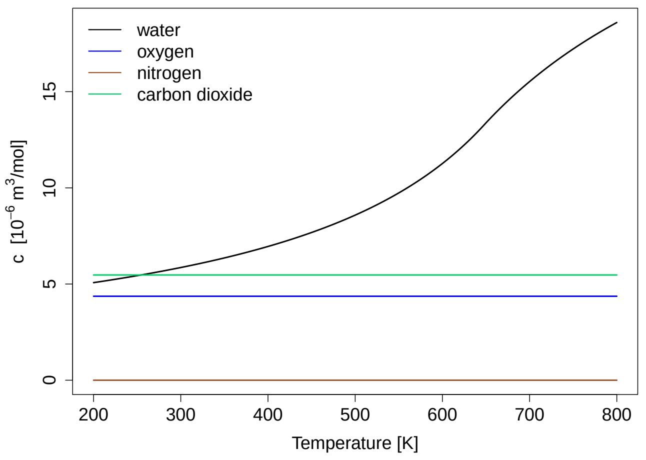

(b) Parameter $c(T)$.

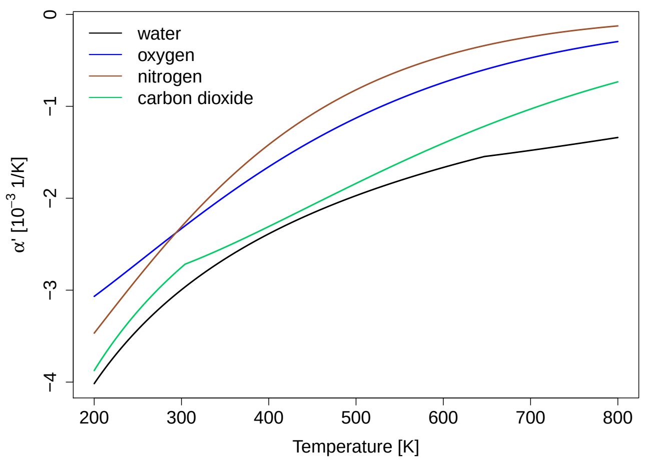

(c) First derivative of $\alpha(T)$.

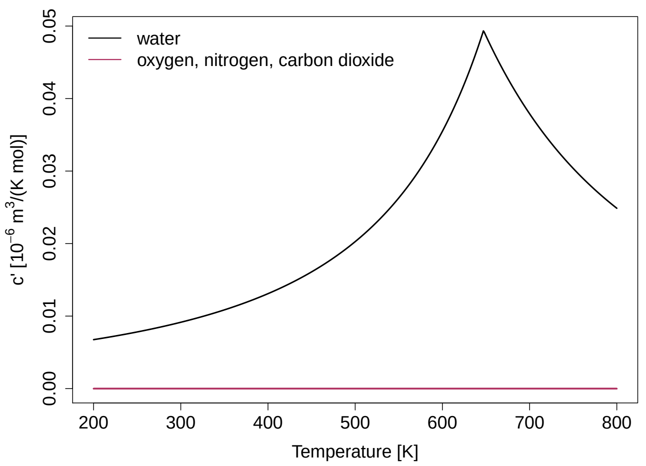

(d) First derivative of $c(T)$.

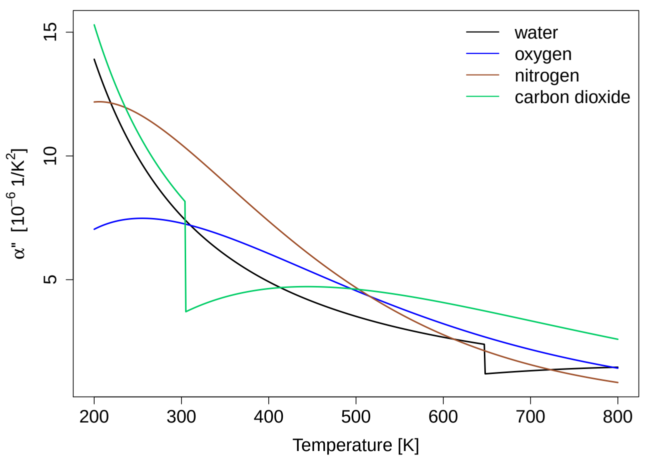

(e) Second derivative of $\alpha(T)$.

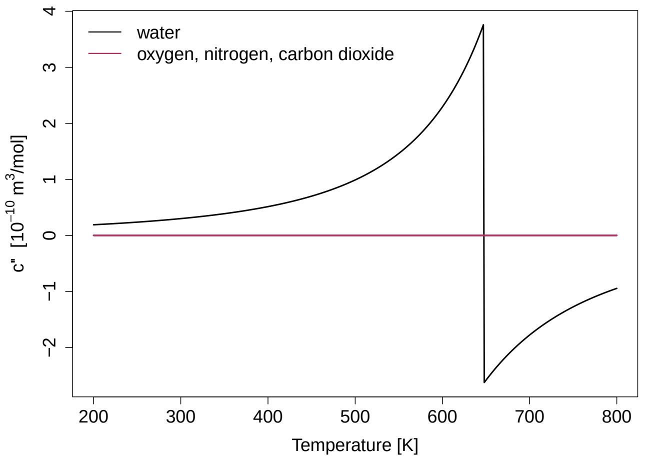

(f) Second derivative of $c(T)$.

Figure S1.1: Comparison of the temperature profiles of parameter α and parameter c and their derivatives depending on the compound (water, oxygen, nitrogen, carbon dioxide).

S1.4. Michelsen-Kistenmacher Syndrome

S1.4.1. Theoretical Approach

Mixing and combining rule for parameter a_{mix}

$$a_{mix} = \sum_i \sum_j x_i x_j \sqrt{a_i a_j} \left(1 - k_{ij} + (x_i - x_j) l_{ij}\right) \tag{S1.49}$$

As our proposed combining rule does not take the therm l_{ij} term into account, Equation (S1.49) becomes

$$a_{mix} = \sum_i \sum_j x_i x_j \sqrt{a_i a_j} \left(1 - k_{ij}\right). \tag{S1.50}$$

For a two compound system

$$a_{mix} = \sum_{i=1}^{2} \sum_{j=1}^{2} x_i x_j \sqrt{a_i a_j} \left(1 - k_{ij}\right) \tag{S1.51}$$

$$= \sum_{i=1}^{2} x_i x_1 \sqrt{a_i a_1} \left(1 - k_{i1}\right) + x_i x_2 \sqrt{a_i a_2} \left(1 - k_{i2}\right) \tag{S1.52}$$

$$\begin{aligned} &= x_1 x_1 \sqrt{a_1 a_1} \left(1 - k_{11}\right) + x_1 x_2 \sqrt{a_1 a_2} \left(1 - k_{12}\right) \\ &\quad + x_2 x_1 \sqrt{a_2 a_1} \left(1 - k_{21}\right) + x_2 x_2 \sqrt{a_2 a_2} \left(1 - k_{22}\right) \end{aligned} \tag{S1.53}$$

$$= x_1^2 a_1 \left(1 - k_{11}\right) + 2 x_1 x_2 \sqrt{a_1 a_2} \left(1 - k_{12}\right) + x_2^2 a_2 \left(1 - k_{22}\right) \tag{S1.54}$$

For a three compound system

$$a_{mix} = \sum_{i=1}^{3} \sum_{j=1}^{3} x_i x_j \sqrt{a_i a_j} \left(1 - k_{ij}\right) \tag{S1.55}$$

$$= \sum_{i=1}^{3} x_i x_1 \sqrt{a_i a_1} \left(1 - k_{i1}\right) + x_i x_2 \sqrt{a_i a_2} \left(1 - k_{i2}\right) + x_i x_3 \sqrt{a_i a_3} \left(1 - k_{i3}\right) \tag{S1.56}$$

$$\begin{aligned} &= x_1 x_1 \sqrt{a_1 a_1} \left(1 - k_{11}\right) + x_1 x_2 \sqrt{a_1 a_2} \left(1 - k_{12}\right) + x_1 x_3 \sqrt{a_1 a_3} \left(1 - k_{13}\right) \\ &\quad + x_2 x_1 \sqrt{a_2 a_1} \left(1 - k_{21}\right) + x_2 x_2 \sqrt{a_2 a_2} \left(1 - k_{22}\right) + x_2 x_3 \sqrt{a_2 a_3} \left(1 - k_{23}\right) \\ &\quad + x_3 x_1 \sqrt{a_3 a_1} \left(1 - k_{31}\right) + x_3 x_2 \sqrt{a_3 a_2} \left(1 - k_{32}\right) + x_3 x_3 \sqrt{a_3 a_3} \left(1 - k_{33}\right) \end{aligned} \tag{S1.57}$$

$$\begin{aligned} &= x_1^2 a_1 \left(1 - k_{11}\right) + 2 x_1 x_2 \sqrt{a_1 a_2} \left(1 - k_{12}\right) + 2 x_1 x_3 \sqrt{a_1 a_3} \left(1 - k_{13}\right) \\ &\quad + x_2^2 a_2 \left(1 - k_{22}\right) + x_2 x_3 \sqrt{a_2 a_3} \left(1 - k_{23}\right) + x_3^2 a_3 \left(1 - k_{33}\right) \end{aligned} \tag{S1.58}$$

$$\begin{aligned} &= x_1^2 a_1 \left(1 - k_{11}\right) + x_2^2 a_2 \left(1 - k_{22}\right) + x_3^2 a_3 \left(1 - k_{33}\right) \\ &\quad + 2 x_1 x_2 \sqrt{a_1 a_2} \left(1 - k_{12}\right) + 2 x_1 x_3 \sqrt{a_1 a_3} \left(1 - k_{13}\right) + 2 x_2 x_3 \sqrt{a_2 a_3} \left(1 - k_{23}\right) \end{aligned} \tag{S1.59}$$

Pseudo three compound system using Eq. (S1.59): $\tilde{x}_2 = 0.5\,x_2$, $\tilde{x}_3 = 0.5\,x_2 \Rightarrow \tilde{x}_2 + \tilde{x}_3 = x_2$

$$\begin{aligned} a_{mix} &= x_1^2 a_1 (1-k_{11}) + \tilde{x}_2^2 a_2 (1-k_{22}) + \tilde{x}_3^2 a_3 (1-k_{33}) \\ &\quad + 2x_1\tilde{x}_2\sqrt{a_1a_2}(1-k_{12}) + 2x_1\tilde{x}_3\sqrt{a_1a_3}(1-k_{13}) + 2\tilde{x}_2\tilde{x}_3\sqrt{a_2a_3}(1-k_{23}) \qquad \text{(S1.60)} \\ &= x_1^2 a_1(1-k_{11}) + \left(\frac{1}{2}x_2\right)^2 a_2(1-k_{22}) + \left(\frac{1}{2}x_2\right)^2 a_3(1-k_{33}) \\ &\quad + 2x_1\left(\frac{1}{2}x_2\right)\sqrt{a_1a_2}(1-k_{12}) + 2x_1\left(\frac{1}{2}x_2\right)\sqrt{a_1a_3}(1-k_{13}) + 2\left(\frac{1}{2}x_2\right)\left(\frac{1}{2}x_2\right)\sqrt{a_2a_3}(1-k_{23}) \qquad \text{(S1.61)} \\ &= x_1^2 a_1(1-k_{11}) + \frac{1}{4}x_2^2 a_2(1-k_{22}) + \frac{1}{4}x_2^2 a_3(1-k_{33}) \\ &\quad + x_1x_2\sqrt{a_1a_2}(1-k_{12}) + x_1x_2\sqrt{a_1a_3}(1-k_{13}) + \frac{1}{2}x_2x_2\sqrt{a_2a_3}(1-k_{23}) \qquad \text{(S1.62)} \\ &\text{with } a_3 = a_2, k_{a,33} = k_{a,22}, k_{a,13} = k_{a,12} \\ &= x_1^2 a_1(1-k_{11}) + \frac{1}{4}x_2^2 a_2(1-k_{22}) + \frac{1}{4}x_2^2 a_2(1-k_{22}) \\ &\quad + x_1x_2\sqrt{a_1a_2}(1-k_{12}) + x_1x_2\sqrt{a_1a_2}(1-k_{12}) + \frac{1}{2}x_2x_2\sqrt{a_2a_2}(1-k_{22}) \qquad \text{(S1.63)} \\ &= x_1^2 a_1(1-k_{11}) + \left(\frac{1}{4}+\frac{1}{4}+\frac{1}{2}\right)x_2^2 a_2(1-k_{22}) + 2x_1x_2\sqrt{a_1a_2}(1-k_{12}) \qquad \text{(S1.64)} \\ &= x_1^2 a_1(1-k_{11}) + x_2^2 a_2(1-k_{22}) + 2x_1x_2\sqrt{a_1a_2}(1-k_{12}) \qquad \text{(S1.65)} \\ & \qquad \text{(S1.66)} \end{aligned}$$

Therefore, when $a_3 = a_2$, $k_{a,33} = k_{a,22}$ and $k_{a,13} = k_{a,12}$, Equation (S1.65) corresponds to the equation for a two compound system (Eq. (S1.54)) and the Michelsen-Kistenmacher syndrome is not relevant for our calculations.
The mentioned criteria are fulfilled when splitting a substance since the same parameter a and interaction parameter coefficients $k_{a,ij}^{(z)}$ are applied at the same temperature.

Mixing and combining rule for parameter b_{mix}

$$b_{mix} = \sum_i \sum_j \frac{1}{2} x_i x_j (b_i + b_j)(1 - k_{b,ij}) \qquad \text{(S1.67)}$$

For a two compound system

$$\begin{aligned} b_{mix} &= \sum_{i=1}^{2}\sum_{j=1}^{2} \frac{1}{2}x_ix_j(b_i+b_j)(1-k_{b,ij}) \qquad \text{(S1.68)} \\ &= \sum_{i=1}^{2} \frac{1}{2}x_ix_1(b_i+b_1)(1-k_{b,i1}) + \frac{1}{2}x_ix_2(b_i+b_2)(1-k_{b,i2}) \qquad \text{(S1.69)} \\ &= \frac{1}{2}x_1x_1(b_1+b_1)(1-k_{b,11}) + \frac{1}{2}x_2x_1(b_2+b_1)(1-k_{b,21}) \\ &\quad + \frac{1}{2}x_1x_2(b_1+b_2)(1-k_{b,12}) + \frac{1}{2}x_2x_2(b_2+b_2)(1-k_{b,22}) \qquad \text{(S1.70)} \\ &= \frac{1}{2}x_1^2(2b_1)(1-k_{b,11}) + 2\cdot\frac{1}{2}x_1x_2(b_1+b_2)(1-k_{b,12}) + \frac{1}{2}x_2^2(2b_2)(1-k_{b,22}) \qquad \text{(S1.71)} \\ &= x_1^2b_1(1-k_{b,11}) + x_1x_2(b_1+b_2)(1-k_{b,12}) + x_2^2b_2(1-k_{b,22}) \qquad \text{(S1.72)} \\ &= x_1^2b_1(1-k_{b,11}) + x_2^2b_2(1-k_{b,22}) + x_1x_2(b_1+b_2)(1-k_{b,12}) \qquad \text{(S1.73)} \end{aligned}$$

For a three compound system

$$b_{mix} = \sum_{i=1}^{3}\sum_{j=1}^{3} \frac{1}{2}x_i x_j(b_i + b_j)(1 - k_{b,ij}) \tag{S1.74}$$

$$= \sum_{i=1}^{2} \frac{1}{2}x_i x_1(b_i + b_1)(1 - k_{b,i1}) + \frac{1}{2}x_i x_2(b_i + b_2)(1 - k_{b,i2}) + \frac{1}{2}x_i x_3(b_i + b_3)(1 - k_{b,i3}) \tag{S1.75}$$

$$\begin{aligned} &= \frac{1}{2}x_1 x_1(b_1 + b_1)(1 - k_{b,11}) + \frac{1}{2}x_2 x_1(b_2 + b_1)(1 - k_{b,21}) + \frac{1}{2}x_3 x_1(b_3 + b_1)(1 - k_{b,31}) \\ &\quad + \frac{1}{2}x_1 x_2(b_1 + b_2)(1 - k_{b,12}) + \frac{1}{2}x_2 x_2(b_2 + b_2)(1 - k_{b,22}) + \frac{1}{2}x_3 x_2(b_3 + b_2)(1 - k_{b,32}) \\ &\quad + \frac{1}{2}x_1 x_3(b_1 + b_3)(1 - k_{b,13}) + \frac{1}{2}x_2 x_3(b_2 + b_3)(1 - k_{b,23}) + \frac{1}{2}x_3 x_3(b_3 + b_3)(1 - k_{b,33}) \end{aligned} \tag{S1.76}$$

$$\begin{aligned} &= \frac{1}{2}x_1^2(2b_1)(1 - k_{b,11}) + 2 \cdot \frac{1}{2}x_1 x_2(b_1 + b_2)(1 - k_{b,12}) + 2 \cdot \frac{1}{2}x_1 x_3(b_1 + b_3)(1 - k_{b,13}) \\ &\quad + \frac{1}{2}x_2^2(2b_2)(1 - k_{b,22}) + 2 \cdot \frac{1}{2}x_2 x_3(b_2 + b_3)(1 - k_{b,23}) + \frac{1}{2}x_3^2(2b_3)(1 - k_{b,33}) \end{aligned} \tag{S1.77}$$

$$\begin{aligned} &= x_1^2 b_1(1 - k_{b,11}) + x_1 x_2(b_1 + b_2)(1 - k_{b,12}) + x_1 x_3(b_1 + b_3)(1 - k_{b,13}) \\ &\quad + x_2^2 b_2(1 - k_{b,22}) + x_2 x_3(b_2 + b_3)(1 - k_{b,23}) + x_3^2 b_3(1 - k_{b,33}) \end{aligned} \tag{S1.78}$$

$$\begin{aligned} &= x_1^2 b_1(1 - k_{b,11}) + x_2^2 b_2(1 - k_{b,22}) + x_3^2 b_3(1 - k_{b,33}) \\ &\quad + x_1 x_2(b_1 + b_2)(1 - k_{b,12}) + x_1 x_3(b_1 + b_3)(1 - k_{b,13}) + x_2 x_3(b_2 + b_3)(1 - k_{b,23}) \end{aligned} \tag{S1.79}$$

Pseudo three compound system using Eq. (S1.79): $\tilde{x}_2 = 0.5\,x_2$, $\tilde{x}_3 = 0.5\,x_2 \Rightarrow \tilde{x}_2 + \tilde{x}_3 = x_2$

$$\begin{aligned} b_{mix} &= x_1^2 b_1(1 - k_{b,11}) + \tilde{x}_2^2 b_2(1 - k_{b,22}) + \tilde{x}_3^2 b_3(1 - k_{b,33}) \\ &\quad + x_1 \tilde{x}_2(b_1 + b_2)(1 - k_{b,12}) + x_1 \tilde{x}_3(b_1 + b_3)(1 - k_{b,13}) + \tilde{x}_2 \tilde{x}_3(b_2 + b_3)(1 - k_{b,23}) \end{aligned} \tag{S1.80}$$

$$\begin{aligned} &= x_1^2 b_1(1 - k_{b,11}) + \left(\frac{1}{2}x_2\right)^2 b_2(1 - k_{b,22}) + \left(\frac{1}{2}x_2\right)^2 b_3(1 - k_{b,33}) \\ &\quad + x_1 \left(\frac{1}{2}x_2\right)(b_1 + b_2)(1 - k_{b,12}) + x_1 \left(\frac{1}{2}x_2\right)(b_1 + b_3)(1 - k_{b,13}) \\ &\quad + \left(\frac{1}{2}x_2\right)\left(\frac{1}{2}x_2\right)(b_2 + b_3)(1 - k_{b,23}) \end{aligned} \tag{S1.81}$$

$$\begin{aligned} &= x_1^2 b_1(1 - k_{b,11}) + \frac{1}{4}x_2^2 b_2(1 - k_{b,22}) + \frac{1}{4}x_2^2 b_3(1 - k_{b,33}) \\ &\quad + \frac{1}{2}x_1 x_2(b_1 + b_2)(1 - k_{b,12}) + \frac{1}{2}x_1 x_2(b_1 + b_3)(1 - k_{b,13}) + \frac{1}{4}x_2^2(b_2 + b_3)(1 - k_{b,23}) \end{aligned} \tag{S1.82}$$

with $b_3 = b_2, k_{b,33} = k_{b,22}, k_{b,13} = k_{b,12}$

$$\begin{aligned} &= x_1^2 b_1(1 - k_{b,11}) + \frac{1}{4}x_2^2 b_2(1 - k_{b,22}) + \frac{1}{4}x_2^2 b_2(1 - k_{b,22}) \\ &\quad + \frac{1}{2}x_1 x_2(b_1 + b_2)(1 - k_{b,12}) + \frac{1}{2}x_1 x_2(b_1 + b_2)(1 - k_{b,12}) + \frac{1}{4}x_2^2(b_2 + b_2)(1 - k_{b,22}) \end{aligned} \tag{S1.83}$$

$$= x_1^2 b_1(1 - k_{b,11}) + 2 \cdot \frac{1}{4}x_2^2 b_2(1 - k_{b,22}) + 2 \cdot \frac{1}{2}x_1 x_2(b_1 + b_2)(1 - k_{b,12}) + 2 \cdot \frac{1}{4}x_2^2 b_2(1 - k_{b,22}) \tag{S1.84}$$

$$= x_1^2 b_1(1 - k_{b,11}) + x_2^2 b_2(1 - k_{b,22}) + x_1 x_2(b_1 + b_2)(1 - k_{b,12}) \tag{S1.85}$$

Therefore, when $b_3 = b_2$, $k_{b,33} = k_{b,22}$ and $k_{b,13} = k_{b,12}$, Equation (S1.85) corresponds to the equation for a two compound system (Eq. (S1.73)) and the Michelsen-Kistenmacher syndrome is not relevant for our calculations.
The mentioned criteria are fulfilled when splitting a substance since the same parameter b and interaction parameter coefficients $k_{b,ij}^{(z)}$ are applied at the same temperature.

S1.4.2. Dilution Effect

Michelsen and Kistenmacher [28] point to the dilution effect caused by the l_{ij} term. The l_{ij} term is calculated in the combining rule in a double summation but is involved in a product of three molar fractions. Since the l_{ij} term is omitted in our combining rule, the calculations are not affected by the dilution effect.

S1.4.3. Pseudo Quaternary System of Water

To investigate a potential Michelsen-Kistenmacher syndrome, the one compound system of water vapor is recalculated with the EOS for a quaternary system varying the molar fractions and compared to the results of the EOS for a single compound system.

In all variations of calculations the same results for molar volume, enthalpy, and the heat capacities are calculated. The results are shown in a summarized form in Table S1.8 where the averaged relatives errors are listed. The equal values for the particular properties represent that the underlying results are equal otherwise the averaged absolute relative errors would vary. Therefore, no Michelsen-Kistenmacher syndrom is observable.

Table S1.8: Comparison of the calculated averaged absolute relative errors for one compound and pseudo multi-compound system of water [27].

Composition				Averaged absolute relative errors and standard deviations			
x_{H_2O} comp. 1	x_{H_2O} comp. 2	x_{H_2O} comp. 3	x_{H_2O} comp. 4	v_m [%]	h [%]s	c_v [%]	c_p [%]
1				2.2 ± 0.1	0.85 ± 0.03	10.4 ± 0.3	6.7 ± 0.3
1	0	0	0	2.2 ± 0.1	0.85 ± 0.03	10.4 ± 0.3	6.7 ± 0.3
0.99	0.01	0	0	2.2 ± 0.1	0.85 ± 0.03	10.4 ± 0.3	6.7 ± 0.3
0.5	0.5	0	0	2.2 ± 0.1	0.85 ± 0.03	10.4 ± 0.3	6.7 ± 0.3

S1.4.4. Pseudo Quaternary System of H_2O/N_2

Further, a potential Michelsen-Kistenmacher syndrome is tested with the binary mixture H_2O/N_2. The binary system is extended to a ternary and quaternary system the molar fractions of water and nitrogen.

In all variations of calculations the same results for molar volume, enthalpy, and the heat capacities are calculated. The results are shown in a summarized form in Table S1.9 where the averaged relatives errors are listed. The equal values for the particular properties represent that the underlying results are equal otherwise the averaged absolute relative errors would vary. Therefore, no Michelsen-Kistenmacher syndrom is observable.

Table S1.9: Comparison of the calculated averaged absolute relative errors for pseudo multi-compound systems of H_2O/N_2 [29].

Composition				Averaged absolute relative errors and standard deviations
$\bar{x}_{H_2O}$ comp. 1	$\bar{x}_{H_2O}$ comp. 2	$\bar{x}_{N_2}$ comp. 3	$\bar{x}_{N_2}$ comp. 4	p [%]
1	0	1	0	6.6 ± 0.6
0.5	0.5	1	0	6.6 ± 0.6
1	0	0.5	0.5	6.6 ± 0.6
0.5	0.5	0.5	0.5	6.6 ± 0.6

Remark: The pseudo molar fraction $\bar{x}_i$ indicates the fraction of the particular molar fraction x_i in the mixture.

$$x_{H_2O} = x_{H_2O} \cdot \bar{x}_{H_2O,\text{comp. 1}} + x_{H_2O} \cdot \bar{x}_{H_2O,\text{comp. 2}} \tag{S1.86}$$

$$x_{N_2} = x_{N_2} \cdot \bar{x}_{N_2,\text{comp. 3}} + x_{N_2} \cdot \bar{x}_{N_2,\text{comp. 4}} \tag{S1.87}$$

$$x_{H_2O} + x_{N_2} = 1 \tag{S1.88}$$

S2. Other Equations of State

S2.1. Redlich-Kwong

Equation of state

$$p = \frac{RT}{v-b} - \frac{a \cdot 1/\sqrt{T}}{v(v+b)} \tag{S2.89}$$

Residual enthalpy

$$h_{res} = RT - \frac{vRT}{v-b} + \frac{a_c\,\alpha}{v+b} + \frac{a_c\,(\alpha' T - \alpha)}{b}\,\ln\left|\frac{v}{v+b}\right| \tag{S2.90}$$

Ideal enthalpy

$$h_{ig} = h_0 + \int_{T_0}^{T} c_p^0(T)\,dT \tag{S2.91}$$

Enthalpy

$$h = h_0 + \int_{T_0}^{T} c_p^0(T)\,dT - RT + \frac{vRT}{v-b} - \frac{a_c\,\alpha}{v+b} - \frac{a_c\,(\alpha' T - \alpha)}{b}\,\ln\left|\frac{v}{v+b}\right| \tag{S2.92}$$

Heat capacity at constant volume

$$c_v = c_p^0(T) - R - \frac{a_c\,\alpha'' T}{b}\,\ln\left|\frac{v}{v+b}\right| \tag{S2.93}$$

Heat capacity at constant pressure

$$c_p = c_v - T\,\frac{\left(\frac{\partial p}{\partial T}\right)_v^2}{\left(\frac{\partial p}{\partial v}\right)_T} \tag{S2.94}$$

with

$$\left.\frac{\partial p}{\partial T}\right|_v = \frac{R}{v-b} - \frac{a_c\,\alpha'}{v(v+b)} \tag{S2.95}$$

and

$$\left.\frac{\partial p}{\partial v}\right|_T = -\frac{RT}{(v-b)^2} + a_c\,\alpha\left(\frac{2v+b}{v^2(v+b)^2}\right) \tag{S2.96}$$

Fugacity coefficient

$$\ln\phi = \frac{v}{v-b} - \frac{a_c\,\alpha}{RT}\frac{1}{(v+b)} - 1 - \ln\left(\frac{v}{v-b} - \frac{a_c\,\alpha}{RT}\frac{1}{(v+b)}\right) - \ln\left|\frac{v-b}{v}\right| + \frac{a_c\alpha}{bRT}\ln\left|\frac{v}{v+b}\right| \tag{S2.97}$$

S2.1.1. Parameter α and its Derivatives

- α

$$\alpha = \frac{1}{\sqrt{T}} \tag{S2.98}$$

- α'

$$\alpha' = -\frac{1}{2\,T^{3/2}} \tag{S2.99}$$

- α''

$$\alpha'' = \frac{3}{4\,T^{5/2}} \tag{S2.100}$$

S2.2. Redlich-Kwong-Soave

Equation of state

$$p = \frac{RT}{v-b} - \frac{a_c\,\alpha}{v(v+b)} \tag{S2.101}$$

$$\sqrt{\alpha} = 1 + m\left(1 - \sqrt{T_r}\right) \tag{S2.102}$$

$$m = 0.480 + 1.574\,\omega - 0.176\,\omega^2 \tag{S2.103}$$

$$a_c = 0.42747\,\frac{R^2\,T_c^2}{p_c} \tag{S2.104}$$

$$b = 0.08664\,\frac{R\,T_c}{p_c} \tag{S2.105}$$

Residual enthalpy

$$h_{res} = RT - \frac{vRT}{v-b} + \frac{a_c\,\alpha}{v+b} + \frac{a_c\,(\alpha' T - \alpha)}{b}\,\ln\left|\frac{v}{v+b}\right| \tag{S2.106}$$

Ideal enthalpy

$$h_{ig} = h_0 + \int_{T_0}^{T} c_p^0(T)\,dT \tag{S2.107}$$

Enthalpy

$$h = h_0 + \int_{T_0}^{T} c_p^0(T)\,dT - RT + \frac{vRT}{v-b} - \frac{a_c\,\alpha}{v+b} - \frac{a_c\,(\alpha' T - \alpha)}{b}\,\ln\left|\frac{v}{v+b}\right| \tag{S2.108}$$

Heat capacity at constant volume

$$c_v = c_p^0(T) - R - \frac{a_c\,\alpha'' T}{b}\ln\left|\frac{v}{v+b}\right| \tag{S2.109}$$

Heat capacity at constant pressure

$$c_p = c_v - T\,\frac{\left(\frac{\partial p}{\partial T}\right)_v^2}{\left(\frac{\partial p}{\partial v}\right)_T} \tag{S2.110}$$

with

$$\left.\frac{\partial p}{\partial T}\right|_v = \frac{R}{v-b} - \frac{a_c\,\alpha'}{v(v+b)} \tag{S2.111}$$

and

$$\left.\frac{\partial p}{\partial v}\right|_T = -\frac{RT}{(v-b)^2} + a_c\,\alpha\left(\frac{2v+b}{v^2(v+b)^2}\right) \tag{S2.112}$$

Fugacity coefficient

$$\ln\phi = \frac{v}{v-b} - \frac{a_c\,\alpha}{RT}\frac{1}{(v+b)} - 1 - \ln\left(\frac{v}{v-b} - \frac{a_c\,\alpha}{RT}\frac{1}{(v+b)}\right) - \ln\left|\frac{v-b}{v}\right| + \frac{a_c\alpha}{bRT}\ln\left|\frac{v}{v+b}\right| \tag{S2.113}$$

S2.2.1. Parameter α and its Derivatives

- α

$$\alpha = \left[1 + m\left(1 - \sqrt{\frac{T}{T_c}}\right)\right]^2 \tag{S2.114}$$

- α'

$$\alpha' = -\frac{m\left(1 + m\left(1 - \sqrt{\frac{T}{T_c}}\right)\right)}{\sqrt{T_c T}} \tag{S2.115}$$

- α''

$$\alpha'' = \frac{m\,(m+1)}{2\,T_c T \sqrt{\frac{T}{T_c}}} \tag{S2.116}$$

S2.3. Peng-Robinson

Equation of state

$$p = \frac{RT}{v-b} - \frac{a_c\,\alpha}{v(v+b)+b(v-b)} \tag{S2.117}$$

$$\sqrt{\alpha} = 1 + \kappa\left(1-\sqrt{T_r}\right) \tag{S2.118}$$

$$\kappa = 0.37464 + 1.54226\,\omega - 0.26992\,\omega^2 \tag{S2.119}$$

$$a_c = 0.45724\,\frac{R^2\,T_c^2}{p_c} \tag{S2.120}$$

$$b = 0.07780\,\frac{R\,T_c}{p_c} \tag{S2.121}$$

Residual enthalpy

$$h_{res} = RT - \frac{vRT}{v-b} + \frac{a_c\,\alpha\,v}{v^2+2bv-b^2} + \frac{a_c\,(\alpha'\,T-\alpha)}{2\sqrt{2}b}\,\ln\left|\frac{v+(1-\sqrt{2})b}{v+(1+\sqrt{2})b}\right| \tag{S2.122}$$

Ideal enthalpy

$$h_{ig} = h_0 + \int_{T_0}^{T} c_p^0(T)\,dT \tag{S2.123}$$

Enthalpy

$$h = h_0 + \int_{T_0}^{T} c_p^0(T)\,dT - RT + \frac{vRT}{v-b} - \frac{a_c\,\alpha\,v}{v^2+2bv-b^2} - \frac{a_c\,(\alpha'\,T-\alpha)}{2\sqrt{2}b}\,\ln\left|\frac{v+(1-\sqrt{2})b}{v+(1+\sqrt{2})b}\right| \tag{S2.124}$$

Heat capacity at constant volume

$$c_v = c_p^0(T) - R - \frac{a_c\,\alpha''\,T}{2\sqrt{2}b}\,\ln\left|\frac{v+(1-\sqrt{2})b}{v+(1+\sqrt{2})b}\right| \tag{S2.125}$$

Heat capacity at constant pressure

$$c_p = c_v - T\,\frac{\left(\frac{\partial p}{\partial T}\right)_v^2}{\left(\frac{\partial p}{\partial v}\right)_T} \tag{S2.126}$$

with

$$\left.\frac{\partial p}{\partial T}\right|_v = \frac{R}{v-b} - \frac{a_c\,\alpha'}{v^2+2bv-b^2} \tag{S2.127}$$

and

$$\left.\frac{\partial p}{\partial v}\right|_T = -\frac{RT}{(v-b)^2} + \frac{2\,a_c\,\alpha\,(v+b)}{(v^2+2bv-b^2)^2} \tag{S2.128}$$

Fugacity coefficient

$$\ln\phi = \frac{v}{v-b} - \frac{a_c\,\alpha}{RT}\,\frac{v}{v^2+2bv-b^2} - 1 - \ln\left(\frac{v}{v-b} - \frac{a_c\,\alpha}{RT}\,\frac{v}{v^2+2bv-b^2}\right) - \ln\left|\frac{v-b}{v}\right| + \frac{a_c\,\alpha}{2\sqrt{2}\,bRT}\,\ln\left|\frac{v+(1-\sqrt{2})b}{v+(1+\sqrt{2})b}\right| \tag{S2.129}$$

S2.3.1. Parameter α and its Derivatives

- α

$$\alpha = \left[1 + \kappa \left(1 - \sqrt{\frac{T}{T_c}}\right)\right]^2 \tag{S2.130}$$

- α'

$$\alpha' = -\frac{\kappa \left(1 + \kappa \left(1 - \sqrt{\frac{T}{T_c}}\right)\right)}{\sqrt{T_c T}} \tag{S2.131}$$

- α''

$$\alpha'' = \frac{\kappa\,(\kappa + 1)}{2\,T_c T \sqrt{\frac{T}{T_c}}} \tag{S2.132}$$

S2.4. VTBMSR-I

Equation of state

$$p = \frac{RT}{v+c-b} - \frac{a_c\,\alpha}{(v+c)(v+c+b)} \tag{S2.133}$$

$$\sqrt{\alpha} = \begin{cases} \exp\left\{c_d(1-T_r^d)\right\} & T_r \le 1 \\ 1+m(1-\sqrt{T_r}) - p_0(1-T_r)(1+p_1T_r+p_2T_r^2) & T_r > 1 \end{cases} \tag{S2.134}$$

$$m = 0.48508 + 1.55191\,\omega - 0.15613\,\omega^2 \tag{S2.135}$$

$$d = 1 + 0.5\,m - p_0\,(1+p_1+p_2) \tag{S2.136}$$

$$c_d = 1 - \frac{1}{d} \tag{S2.137}$$

$$c = \begin{cases} c_0 + \frac{c_1}{1+c_2-T_r} & T_r \le 1 \\ c_0 & T_r > 1, c_1 = 0 \\ b + \frac{\left(\frac{(c_0-b)c_2}{c_1}+1\right)^2 c_1}{1+c_2\left(\frac{(c_0-b)c_2}{c_1}+1\right)-T_r} & T_r > 1, c_1 \ne 0 \end{cases} \tag{S2.138}$$

$$a_c = \frac{1}{9(2^{1/3}-1)}\,\frac{R^2\,T_c^2}{p_c} \tag{S2.139}$$

$$b = \frac{1}{3}(2^{1/3}-1)\,\frac{R\,T_c}{p_c} \tag{S2.140}$$

Residual enthalpy

$$h_{res} = RT - v\left(\frac{RT}{v+c-b} - \frac{a_c\,\alpha}{(v+c)(v+c+b)}\right) + \frac{a_c(\alpha' T - \alpha)}{b}\ln\left|\frac{v+c}{v+c+b}\right| - \frac{RT^2c'}{v+c-b} + \frac{a_c\,\alpha c' T}{(v+c)(v+c+b)} \tag{S2.141}$$

Ideal enthalpy

$$h_{ig} = h_0 + \int_{T_0}^{T} c_p^0(T)\,dT \tag{S2.142}$$

Enthalpy

$$h = h_0 + \int_{T_0}^{T} c_p^0(T)\,dT - RT + \frac{RTv}{v+c-b} - \frac{a_c\,\alpha v}{(v+c)(v+c+b)} - \frac{a_c(\alpha' T - \alpha)}{b}\ln\left|\frac{v+c}{v+c+b}\right| + \frac{RT^2c'}{v+c-b} - \frac{a_c\,\alpha c' T}{(v+c)(v+c+b)} \tag{S2.143}$$

Heat capacity at constant volume

$$c_v = c_p^0(T) - R - \frac{a_c\,\alpha'' T}{b}\,\ln\left|\frac{v+c}{v+c+b}\right| + \frac{2RTc' + RT^2c''}{v+c-b} - \frac{R(c'T)^2}{(v+c-b)^2} - \frac{2a_c\,\alpha' c' T + a_c\,\alpha c'' T}{(v+c)(v+c+b)} + \frac{a_c\;\alpha(c')^2T(2(V+c)+b)}{(v+c)^2(v+c+b)^2} \tag{S2.144}$$

Heat capacity at constant pressure

$$c_p = c_v - T\,\frac{\left(\frac{\partial p}{\partial T}\right)_v^2}{\left(\frac{\partial p}{\partial v}\right)_T} \tag{S2.145}$$

with

$$\left.\frac{\partial p}{\partial T}\right|_v = \frac{R}{v+c-b} - \frac{RTc'}{(v+c-b)^2} - \frac{a_c\,\alpha'}{(v+c)(v+c+b)} + \frac{a_c\,\alpha c'(2(v+c)+b)}{(v+c)^2(v+c+b)^2} \tag{S2.146}$$

and

$$\left.\frac{\partial p}{\partial v}\right|_T = -\frac{RT}{(v+c-b)^2} + a_c\,\alpha\left(\frac{2(v+c)+b}{(v+c)^2(v+c+b)^2}\right) \tag{S2.147}$$

Fugacity coefficient

$$\begin{aligned}\ln\phi = {} & \frac{v}{v+c-b} - \frac{a_c\,\alpha}{RT}\frac{v}{(v+c)(v+c+b)} - 1 - \ln\left(\frac{v}{v+c-b} - \frac{a_c\,\alpha}{RT}\frac{v}{(v+c)(v+c+b)}\right) \\ & - \ln\left|\frac{v+c-b}{v}\right| + \frac{a_c\,\alpha}{bRT}\ln\left|\frac{v+c}{v+c+b}\right|\end{aligned} \tag{S2.148}$$

S2.4.1. Parameter α and its Derivatives

- α

$$\alpha = \begin{cases}\left[\exp\left\{c_d(1-T_r^d)\right\}\right]^2 & T_r \le 1 \\ \left[1+m(1-\sqrt{T_r}) - p_0(1-T_r)(1+p_1T_r+p_2T_r^2)\right]^2 & T_r > 1\end{cases} \tag{S2.149}$$

- α'
  - $T_r \le 1$

$$\alpha' = -2\,c_d d\frac{1}{T_c}\left(\frac{T}{T_c}\right)^{d-1}\exp\left[2\,c_d\left(1-\left(\frac{T}{T_c}\right)^d\right)\right] \tag{S2.150}$$

  - $T_r > 1$

$$\begin{aligned}\alpha' = {} & 2\cdot\left[1+m(1-\sqrt{T_r}) - p_0(1-T_r)(1+p_1T_r+p_2T_r^2)\right] \\ & \cdot\left[-\frac{m}{2\,T_c\sqrt{\frac{T}{T_c}}} + \frac{3\,p_0p_2T^2}{T_c^3} + 2\,p_0\,(p_1-p_2)\,\frac{T}{T_c^2} + p_0\,(1-p_1)\,\frac{1}{T_c}\right]\end{aligned} \tag{S2.151}$$

- α''
  - $T_r \le 1$

$$\alpha'' = -2\,c_d d\,(d-1)\,\frac{1}{T_c^2}\left(\frac{T}{T_c}\right)^{d-2}\exp\left[2\,c_d\left(1-\left(\frac{T}{T_c}\right)^d\right)\right] \tag{S2.152}$$

$$+\ 4\,c_d^2d^2\frac{1}{T_c^2}\left(\frac{T}{T_c}\right)^{2(d-1)}\exp\left[2\,c_d\left(1-\left(\frac{T}{T_c}\right)^d\right)\right] \tag{S2.153}$$

  - $T_r > 1$

$$\begin{aligned}\alpha'' = {} & 2\cdot\left[1+m(1-\sqrt{T_r}) - p_0(1-T_r)(1+p_1T_r+p_2T_r^2)\right] \\ & \cdot\left[\frac{m}{4\,T_c^2\left(\frac{T}{T_c}\right)^{3/2}} + 2\,p_0\,(p_1-p_2)\,\frac{1}{T_c^2} + 6\,p_0p_2\left(\frac{T}{T_c^3}\right)\right] \\ & + 2\cdot\left[-\frac{m}{2\,T_c\sqrt{\frac{T}{T_c}}} + \frac{3\,p_0p_2T^2}{T_c^3} + 2\,p_0\,(p_1-p_2)\,\frac{T}{T_c^2} + p_0\,(1-p_1)\,\frac{1}{T_c}\right]^2\end{aligned} \tag{S2.154}$$

S2.4.2. Parameter c and its Derivatives

- c

$$c = \begin{cases} c_0 + \frac{c_1}{1+c_2-T_r} & T_r \leq 1 \\ c_0 & T_r > 1, c_1 = 0 \\ b + \frac{\left(\frac{(c_0-b)c_2}{c_1}+1\right)^2 c_1}{1+c_2\left(\frac{(c_0-b)c_2}{c_1}+1\right)-T_r} & T_r > 1, c_1 \neq 0 \end{cases} \tag{S2.155}$$

- c'
    - $T_r \leq 1$

$$c' = \frac{c_1}{\left(1+c_2-\frac{T}{T_c}\right)^2 T_c} \tag{S2.156}$$

    - $T_r > 1$

$$c' = \frac{\left(\frac{(c_0-b)c_2}{c_1}+1\right)^2 c_1}{\left(1+c_2\left(\frac{(c_0-b)c_2}{c_1}+1\right)-\frac{T}{T_c}\right)^2 T_c} \tag{S2.157}$$

- c''
    - $T_r \leq 1$

$$c'' = \frac{2\,c_1}{\left(1+c_2-\frac{T}{T_c}\right)^3 T_c^2} \tag{S2.158}$$

    - $T_r > 1$

$$c'' = \frac{2\left(\frac{(c_0-b)c_2}{c_1}+1\right)^2 c_1}{\left(1+c_2\left(\frac{(c_0-b)c_2}{c_1}+1\right)-\frac{T}{T_c}\right)^3 T_c^2} \tag{S2.159}$$

S2.5. VTBMSR-II
Equation of state

$$p = \frac{RT}{v+c-b} - \frac{a_c\,\alpha}{(v+c)(v+c+b)} \tag{S2.160}$$

$$\sqrt{\alpha} = \begin{cases} \exp\{c_d(1-T_r\} & T_r \le 1 \\ 1+m(1-\sqrt{T_r}) - p_0(1-T_r)(1+p_1T_r+p_2T_r^2) & T_r > 1 \end{cases} \tag{S2.161}$$

$$m = 0.48508 + 1.55191\,\omega - 0.15613\,\omega^2 \tag{S2.162}$$

$$d = 1 + 0.5\,m - p_0\,(1+p_1+p_2) \tag{S2.163}$$

$$c_d = 1 - \frac{1}{d} \tag{S2.164}$$

$$c = \begin{cases} c_0 + \frac{c_1}{1+c_2-T_r} & T_r \le 1 \\ c_0 & T_r > 1, c_1 = 0 \\ b + \frac{\left(\frac{(c_0-b)c_2}{c_1}+1\right)^2 c_1}{1+c_2\left(\frac{(c_0-b)c_2}{c_1}+1\right)-T_r} & T_r > 1, c_1 \ne 0 \end{cases} \tag{S2.165}$$

$$a_c = \frac{1}{9(2^{1/3}-1)}\,\frac{R^2\,T_c^2}{p_c} \tag{S2.166}$$

$$b = \frac{1}{3}(2^{1/3}-1)\,\frac{R\,T_c}{p_c} \tag{S2.167}$$

Residual enthalpy

$$h_{res} = RT - v\left(\frac{RT}{v+c-b} - \frac{a_c\,\alpha}{(v+c)(v+c+b)}\right) + \frac{a_c(\alpha' T - \alpha)}{b}\ln\left|\frac{v+c}{v+c+b}\right| - \frac{RT^2c'}{v+c-b} + \frac{a_c\,\alpha c' T}{(v+c)(v+c+b)} \tag{S2.168}$$

Ideal enthalpy

$$h_{ig} = h_0 + \int_{T_0}^{T} c_p^0(T)\,dT \tag{S2.169}$$

Enthalpy

$$h = h_0 + \int_{T_0}^{T} c_p^0(T)\,dT - RT + \frac{RTv}{v+c-b} - \frac{a_c\,\alpha v}{(v+c)(v+c+b)} - \frac{a_c(\alpha' T - \alpha)}{b}\ln\left|\frac{v+c}{v+c+b}\right| + \frac{RT^2c'}{v+c-b} - \frac{a_c\,\alpha c' T}{(v+c)(v+c+b)} \tag{S2.170}$$

Heat capacity at constant volume

$$c_v = c_p^0(T) - R - \frac{a_c\,\alpha'' T}{b}\,\ln\left|\frac{v+c}{v+c+b}\right| + \frac{2RTc' + RT^2c''}{v+c-b} - \frac{R(c'T)^2}{(v+c-b)^2} - \frac{2a_c\,\alpha' c' T + a_c\,\alpha c'' T}{(v+c)(v+c+b)} + \frac{a_c\,\alpha(c')^2T(2(V+c)+b)}{(v+c)^2(v+c+b)^2} \tag{S2.171}$$

Heat capacity at constant pressure

$$c_p = c_v - T\,\frac{\left(\frac{\partial p}{\partial T}\right)_v^2}{\left(\frac{\partial p}{\partial v}\right)_T} \tag{S2.172}$$

with

$$\left.\frac{\partial p}{\partial T}\right|_v = \frac{R}{v+c-b} - \frac{RTc'}{(v+c-b)^2} - \frac{a_c\,\alpha'}{(v+c)(v+c+b)} + \frac{a_c\,\alpha c'(2(v+c)+b)}{(v+c)^2(v+c+b)^2} \tag{S2.173}$$

and

$$\left.\frac{\partial p}{\partial v}\right|_T = -\frac{RT}{(v+c-b)^2} + a_c\,\alpha\left(\frac{2(v+c)+b}{(v+c)^2(v+c+b)^2}\right) \tag{S2.174}$$

Fugacity coefficient

$$\begin{aligned}\ln\phi = &\frac{v}{v+c-b} - \frac{a_c\,\alpha}{RT}\frac{v}{(v+c)(v+c+b)} - 1 - \ln\left(\frac{v}{v+c-b} - \frac{a_c\,\alpha}{RT}\frac{v}{(v+c)(v+c+b)}\right)\\ &- \ln\left|\frac{v+c-b}{v}\right| + \frac{a_c\,\alpha}{bRT}\ln\left|\frac{v+c}{v+c+b}\right|\end{aligned} \tag{S2.175}$$

S2.5.1. Parameter α and its Derivatives

- α

$$\alpha = \begin{cases}\left[\exp\left\{c_d(1-T_r)\right\}\right]^2 & T_r \leq 1\\ \left[1+m(1-\sqrt{T_r})-p_0(1-T_r)(1+p_1T_r+p_2T_r^2)\right]^2 & T_r > 1\end{cases} \tag{S2.176}$$

- α'
  - $T_r \leq 1$

$$\alpha' = -\frac{2\,c_d}{T_c}\,\exp\left[2\,c_d\left(1-\left(\frac{T}{T_c}\right)\right)\right] \tag{S2.177}$$

  - $T_r > 1$

$$\begin{aligned}\alpha' = 2\cdot&\left[1+m(1-\sqrt{T_r})-p_0(1-T_r)(1+p_1T_r+p_2T_r^2)\right]\\ &\cdot\left[-\frac{m}{2\,T_c\sqrt{\frac{T}{T_c}}} + \frac{3\,p_0p_2T^2}{T_c^3} + 2\,p_0\,(p_1-p_2)\,\frac{T}{T_c^2} + p_0\,(1-p_1)\,\frac{1}{T_c}\right]\end{aligned} \tag{S2.178}$$

- α''
  - $T_r \leq 1$

$$\alpha'' = \frac{4\,c_d^2}{T_c^2}\,\exp\left[2\,c_d\left(1-\left(\frac{T}{T_c}\right)\right)\right] \tag{S2.179}$$

  - $T_r > 1$

$$\begin{aligned}\alpha'' = 2\cdot&\left[1+m(1-\sqrt{T_r})-p_0(1-T_r)(1+p_1T_r+p_2T_r^2)\right]\\ &\cdot\left[\frac{m}{4\,T_c^2\left(\frac{T}{T_c}\right)^{3/2}} + 2\,p_0\,(p_1-p_2)\,\frac{1}{T_c^2} + 6\,p_0p_2\left(\frac{T}{T_c^3}\right)\right]\\ &+\ 2\cdot\left[-\frac{m}{2\,T_c\sqrt{\frac{T}{T_c}}} + \frac{3\,p_0p_2T^2}{T_c^3} + 2\,p_0\,(p_1-p_2)\,\frac{T}{T_c^2} + p_0\,(1-p_1)\,\frac{1}{T_c}\right]^2\end{aligned} \tag{S2.180}$$

S2.5.2. Parameter c and its Derivatives

- c

$$c = \begin{cases} c_0 + \frac{c_1}{1+c_2-T_r} & T_r \leq 1 \\ c_0 & T_r > 1, c_1 = 0 \\ b + \frac{\left(\frac{(c_0-b)c_2}{c_1}+1\right)^2 c_1}{1+c_2\left(\frac{(c_0-b)c_2}{c_1}+1\right)-T_r} & T_r > 1, c_1 \neq 0 \end{cases} \tag{S2.181}$$

- c'
  - $T_r \leq 1$

$$c' = \frac{c_1}{\left(1 + c_2 - \frac{T}{T_c}\right)^2 T_c} \tag{S2.182}$$

  - $T_r > 1$

$$c' = \frac{\left(\frac{(c_0-b)c_2}{c_1} + 1\right)^2 c_1}{\left(1 + c_2\left(\frac{(c_0-b)c_2}{c_1} + 1\right) - \frac{T}{T_c}\right)^2 T_c} \tag{S2.183}$$

- c''
  - $T_r \leq 1$

$$c'' = \frac{2\, c_1}{\left(1 + c_2 - \frac{T}{T_c}\right)^3 T_c^2} \tag{S2.184}$$

  - $T_r > 1$

$$c'' = \frac{2\left(\frac{(c_0-b)c_2}{c_1} + 1\right)^2 c_1}{\left(1 + c_2\left(\frac{(c_0-b)c_2}{c_1} + 1\right) - \frac{T}{T_c}\right)^3 T_c^2} \tag{S2.185}$$

S3. Comparison of Different EOS

S3.1. Density Prediction along Vapor-Liquid Coexistence Curve of Water

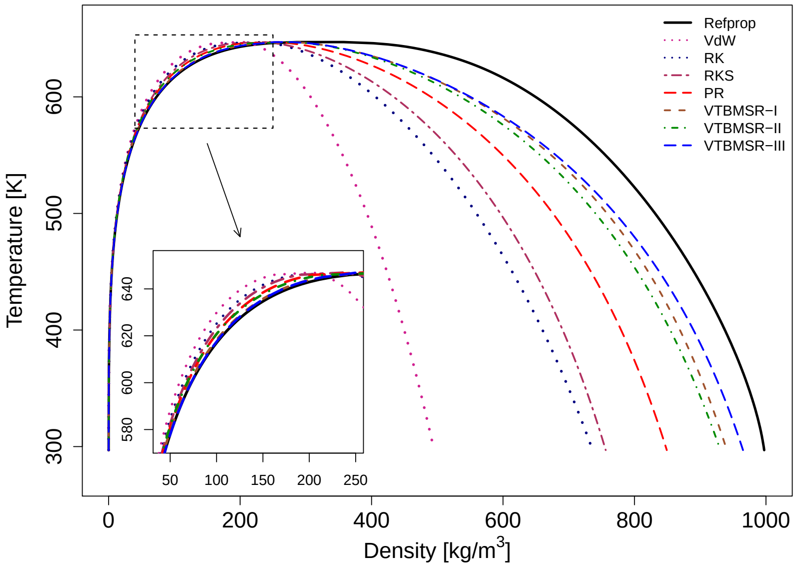

Figure S3.1: Density predictions of water along the vapor-liquid coexistence curve by different EOS. Reference: Refprop [27].

S3.2. Enthalpy Prediction along Vapor-Liquid Coexistence Curve of Water

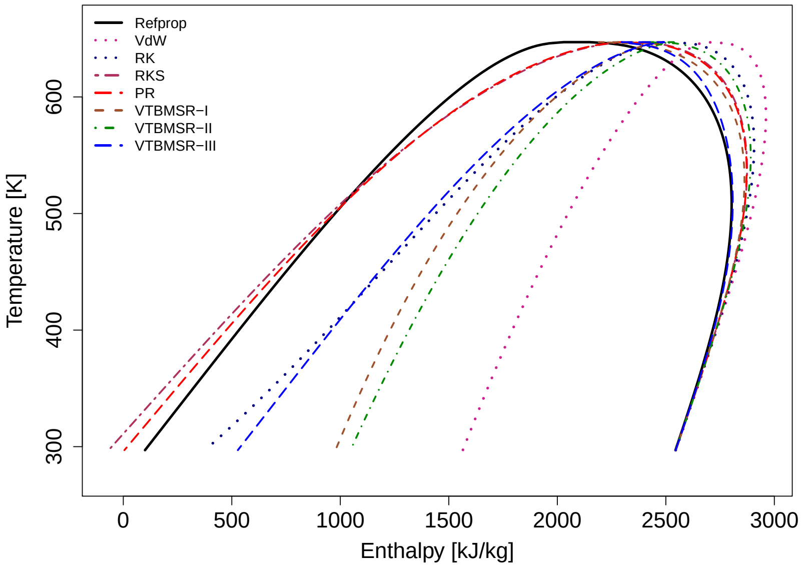

Figure S3.2: Enthalpy predictions of water along the vapor-liquid coexistence curve by different EOS. Reference: Refprop [27].

S3.3. Heat Capacity at Constant Volume Prediction along Vapor-Liquid Coexistence Curve of Water

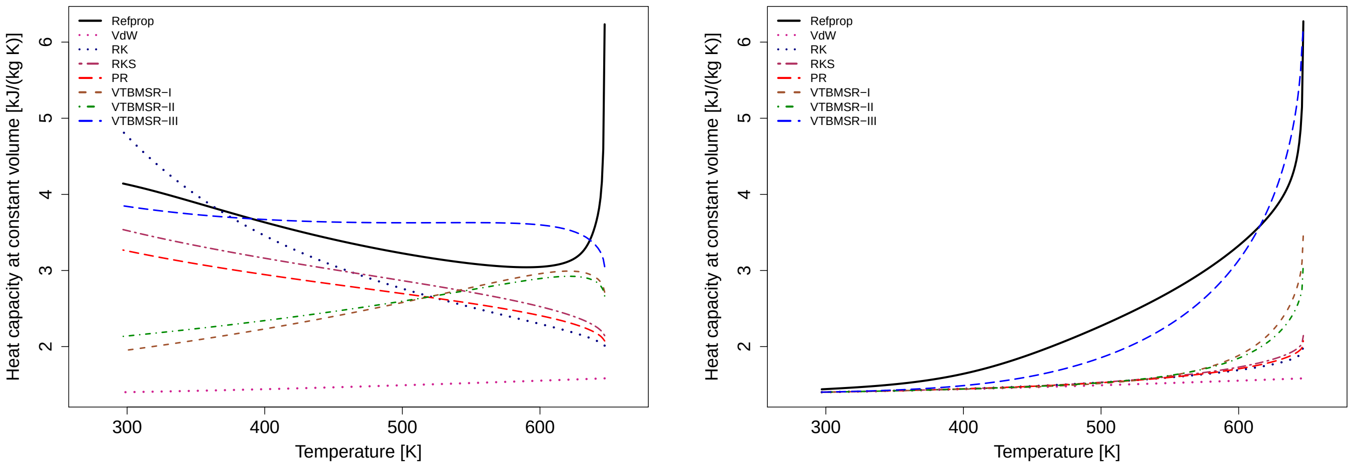

(a) Liquid phase. (b) Vapor phase.

Figure S3.3: Heat capacity at constant volume predictions of water along the vapor-liquid coexistence curve by different EOS. Reference: Refprop [27].

S3.4. Heat Capacity at Constant Pressure Prediction along Vapor-Liquid Coexistence Curve of Water

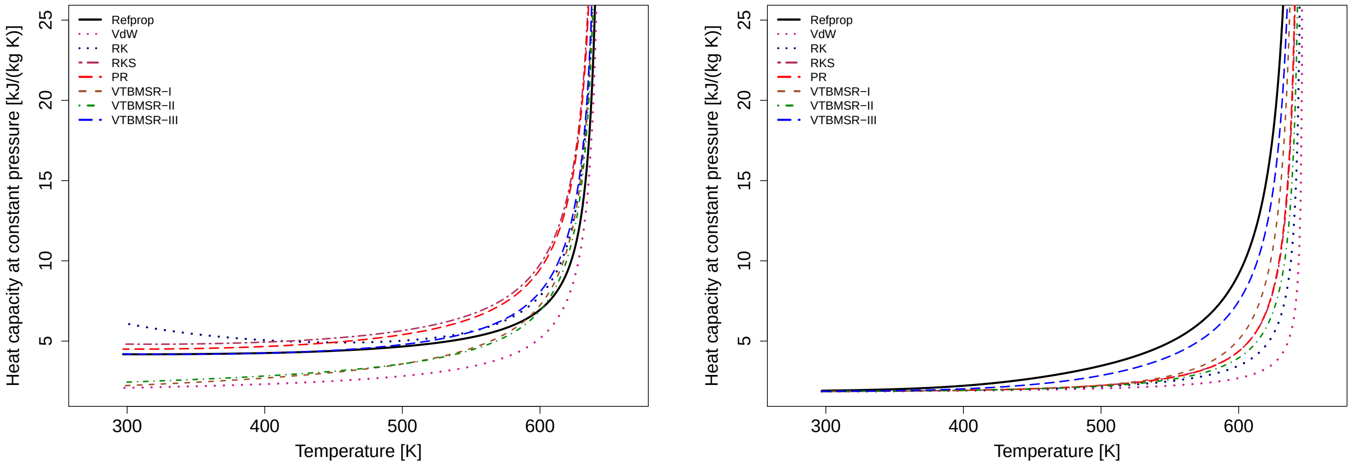

(a) Liquid phase. (b) Vapor phase.

Figure S3.4: Heat capacity at constant pressure predictions of water along the vapor-liquid coexistence curve by different EOS. Reference: Refprop [27].

S3.5. Derivatives of Different α-functions

The first and second derivatives of parameter α with respect to temperature are not generally continuous when α is given by temperature-dependent determination. This causes discontinuities in derived thermodynamic properties (*e.g.* c_v and c_p) that includes these derivatives, see also S1.3.3. In the following sections parameter α and its derivatives are evaluated at the limit temperature $T_r = 1$ for both cases ($T_r \leq 1$ and $T_r > 1$) for the different α functions given by Boston and Mathias [13], Mathias [14], VTBMSR-I [26], -II [11] and -III, see Table 1. Further, the profiles of the parameters are presented graphically (Supporting Information. S3.5.7).

S3.5.1. Boston and Mathias

- α

$$\alpha = \begin{cases} \left[1 + m(1 - \sqrt{T_r})\right]^2 & T_r \leq 1 \\ \exp\left\{c_d(1 - T_r^d)\right\} & T_r > 1 \end{cases} \tag{S3.186}$$

$$d = 1 + \frac{m}{2} \tag{S3.187}$$

$$c_d = \frac{m}{d} \tag{S3.188}$$

  - Left-hand limit for $T_r = 1$

$$\lim_{T_r \nearrow 1} \alpha = 1 \tag{S3.189}$$

  - Right-hand limit for $T_r = 1$

$$\lim_{T_r \searrow 1} \alpha = 1 \tag{S3.190}$$

Equation (S3.189) and Equation (S3.190) yield the same result. Therefore, $\alpha(T)$ is continuous in $T_r = 1$.

- α'

$$\alpha' = \begin{cases} \frac{m}{\sqrt{T_r}}\left(m\sqrt{T_r} - m - 1\right) & T_r \leq 1 \\ -c_d d T_r^{d-1} \exp\left\{c_d(1 - T_r^d)\right\} & T_r > 1 \end{cases} \tag{S3.191}$$

  - Left-hand limit for $T_r = 1$

$$\lim_{T_r \nearrow 1} \alpha' = -m \tag{S3.192}$$

  - Right-hand limit for $T_r = 1$

$$\lim_{T_r \searrow 1} \alpha' = -c_d d = -m \tag{S3.193}$$

Equation (S3.192) and Equation (S3.193)yield the same result. Therefore, $\alpha(T)$ is continuous in $T_r = 1$.

- α''

$$\alpha'' = \begin{cases} \frac{m(m+1)}{2T_r^{3/2}} & T_r \leq 1 \\ \left(c_d^2 d^2 T_r^{2(d-1)} - c_d d(d-1) T_r^{d-2}\right) \exp\left\{c_d(1 - T_r^d)\right\} & T_r > 1 \end{cases} \tag{S3.194}$$

  - Left-hand limit for $T_r = 1$

$$\lim_{T_r \nearrow 1} \alpha'' = \frac{m(m+1)}{2} \tag{S3.195}$$

  - Right-hand limit for $T_r = 1$

$$\lim_{T_r \searrow 1} \alpha'' = c_d^2 d^2 - c_d d(d-1) = \frac{m^2}{2} \tag{S3.196}$$

Equation (S3.195) and Equation (S3.196) yield not the same result for $m \neq 0$. Therefore, $\alpha(T)$ is not continuous at $T_r = 1$.

S3.5.2. Mathias

- α

$$\alpha = \begin{cases} \left[1 + m(1-\sqrt{T_r}) - p_i(1-T_r)(0.7-T_r)\right]^2 & T_r \leq 1 \\ \left[\exp\left\{c_d(1-T_r^d)\right\}\right]^2 & T_r > 1 \end{cases} \tag{S3.197}$$

$$c_d = 1 + \frac{m}{2} + 0.3p_i \tag{S3.198}$$

$$d = \frac{c_d - 1}{c_d} \tag{S3.199}$$

  - Left-hand limit for $T_r = 1$

$$\lim_{T_r \nearrow 1} \alpha = 1 \tag{S3.200}$$

  - Right-hand limit for $T_r = 1$

$$\lim_{T_r \searrow 1} \alpha = 1 \tag{S3.201}$$

Equation (S3.200) and Equation (S3.201) yield the same result. Therefore, $\alpha(T)$ is continuous in $T_r = 1$.

- α'

$$\alpha' = \begin{cases} 2 \cdot \left[1 + m(1-\sqrt{T_r}) - p_i(1-T_r)(0.7-T_r)\right] \cdot \left[-\frac{m}{2\sqrt{T_r}} - p_i\,(2T_r - 1.7)\right] & T_r \leq 1 \\ -2c_d d T_r^{d-1} \exp\left\{2c_d(1-T_r^d)\right\} & T_r > 1 \end{cases} \tag{S3.202}$$

  - Left-hand limit for $T_r = 1$

$$\lim_{T_r \nearrow 1} \alpha' = -m - 0.6\,p_i \tag{S3.203}$$

  - Right-hand limit for $T_r = 1$

$$\lim_{T_r \searrow 1} \alpha' = -2c_d d = -m - 0.6\,p_i \tag{S3.204}$$

Equation (S3.203) and Equation (S3.204) yield the same result. Therefore, $\alpha(T)$ is continuous in $T_r = 1$.

- α''

$$\alpha'' = \begin{cases} 2 \cdot \left[1 + m(1-\sqrt{T_r}) - p_i(1-T_r)(0.7-T_r)\right] \cdot \left[\frac{m}{4T_r^{3/2}} - 2\,p_i\right] + 2 \cdot \left[-\frac{m}{2\sqrt{T_r}} - p_i(2T_r - 1.7)\right]^2 & T_r \leq 1 \\ \left(4c_d^2 d^2 T_r^{2(d-1)} - 2c_d d(d-1) T_r^{d-2}\right) \exp\left\{2c_d(1-T_r^d)\right\} & T_r > 1 \end{cases} \tag{S3.205}$$

  - Left-hand limit for $T_r = 1$

$$\lim_{T_r \nearrow 1} \alpha'' = \frac{m}{2} - 4\,p_i + 2\left[-\frac{m}{2} - 0.3\,p_i\right]^2 \tag{S3.206}$$

  - Right-hand limit for $T_r = 1$

$$\lim_{T_r \searrow 1} \alpha'' = 4c_d^2 d^2 - 2c_d d(d-1) = 2\frac{\frac{m}{2} + 0.3p_i}{\left(1 + \frac{m}{2} + 0.3p_i\right)^2} + 4\frac{\left(\frac{m}{2} + 0.3p_i\right)^2}{\left(1 + \frac{m}{2} + 0.3p_i\right)} \tag{S3.207}$$

Equation (S3.206) and Equation (S3.207) yield *a priori* not the same result indicating that alpha(T) is not obliged to be continuous.

S3.5.3. VTBMSR-I

- α

$$\alpha = \begin{cases} \left[\exp\left\{c_d(1-T_r^d)\right\}\right]^2 & T_r \leq 1 \\ \left[1+m(1-\sqrt{T_r})-p_0(1-T_r)(1+p_1T_r+p_2T_r^2)\right]^2 & T_r > 1 \end{cases} \tag{S3.208}$$

$$d = 1 + \frac{m}{2} - p_0(1+p_1+p_2) \tag{S3.209}$$

$$c_d = 1 - \frac{1}{d} \tag{S3.210}$$

  - Left-hand limit for $T_r = 1$

$$\lim_{T_r \nearrow 1} \alpha = 1 \tag{S3.211}$$

  - Right-hand limit for $T_r = 1$

$$\lim_{T_r \searrow 1} \alpha = 1 \tag{S3.212}$$

Equation (S3.211) and Equation (S3.212) yield the same result. Therefore, $\alpha(T)$ is continuous in $T_r = 1$.

- α'

$$\alpha' = \begin{cases} -2c_d d T_r^{d-1} \exp\left\{2c_d(1-T_r^d)\right\} & T_r \leq 1 \\ 2 \cdot \left[1+m(1-\sqrt{T_r})-p_0(1-T_r)(1+p_1T_r+p_2T_r^2)\right] & T_r > 1 \\ \quad \cdot \left[-\frac{m}{2\sqrt{T_r}} + p_0\left\{(1-p_1)+2(p_1-p_2)T_r+3p_2T_r^2\right\}\right] & \end{cases} \tag{S3.213}$$

  - Left-hand limit for $T_r = 1$

$$\lim_{T_r \nearrow 1} \alpha' = -2c_d d = -m + 2p_0(1+p_1+p_2) \tag{S3.214}$$

  - Right-hand limit for $T_r = 1$

$$\lim_{T_r \searrow 1} \alpha' = -m + 2p_0(1+p_1+p_2) \tag{S3.215}$$

Equation (S3.214) and Equation (S3.215) yield the same result. Therefore, $\alpha(T)$ is continuous in $T_r = 1$.

- α''

$$\alpha'' = \begin{cases} \left(4c_d^2 d^2 T_r^{2(d-1)} - 2c_d d(d-1)T_r^{d-2}\right)\exp\left\{2c_d(1-T_r^d)\right\} & T_r \leq 1 \\ 2 \cdot \left[1+m(1-\sqrt{T_r})-p_0(1-T_r)(1+p_1T_r+p_2T_r^2)\right] & T_r > 1 \\ \quad \cdot \left[\frac{m}{4T_r^{3/2}} + 2p_0(p_1-p_2) + 6p_0p_2T_r\right] & \\ \quad +2 \cdot \left[-\frac{m}{2\sqrt{T_r}} + p_0\left\{(1-p_1)+2(p_1-p_2)T_r+3p_2T_r^2\right\}\right]^2 & \end{cases} \tag{S3.216}$$

  - Left-hand limit for $T_r = 1$

$$\lim_{T_r \nearrow 1} \alpha'' = 4c_d^2 d^2 - 2c_d d(d-1) = 2\left(\frac{m}{2} - p_0(1+p_1+p_2)\right)^2 \tag{S3.217}$$

  - Right-hand limit for $T_r = 1$

$$\lim_{T_r \searrow 1} \alpha'' = \frac{m}{2} + 4p_0p_1 + 8p_0p_2 + 2\left(\frac{m}{2} - p_0(1+p_1+p_2)\right)^2 \tag{S3.218}$$

Equation (S3.217) and Equation (S3.218) yield *a priori* not the same result indicating that alpha(T) is not obliged to be continuous.

S3.5.4. VTBMSR-II

- α

$$\alpha = \begin{cases} \left[\exp\left\{c_d(1-T_r)\right\}\right]^2 & T_r \leq 1 \\ \left[1+m(1-\sqrt{T_r})-p_0(1-T_r)(1+p_1T_r+p_2T_r^2)\right]^2 & T_r > 1 \end{cases} \tag{S3.219}$$

$$d = 1+\frac{m}{2}-p_0(1+p_1+p_2) \tag{S3.220}$$

$$c_d = 1-\frac{1}{d} \tag{S3.221}$$

  - Left-hand limit for $T_r = 1$

$$\lim_{T_r \nearrow 1} \alpha = 1 \tag{S3.222}$$

  - Right-hand limit for $T_r = 1$

$$\lim_{T_r \searrow 1} \alpha = 1 \tag{S3.223}$$

  Equation (S3.222) and Equation (S3.223) yield the same result. Therefore, $\alpha(T)$ is continuous in $T_r = 1$.

- α'

$$\alpha' = \begin{cases} -2c_d \exp\left\{2c_d(1-T_r)\right\} & T_r \leq 1 \\ 2\cdot\left[1+m(1-\sqrt{T_r})-p_0(1-T_r)(1+p_1T_r+p_2T_r^2)\right] & T_r > 1 \\ \quad \cdot\left[-\frac{m}{2\sqrt{T_r}}+p_0\left\{(1-p_1)+2(p_1-p_2)T_r+3p_2T_r^2\right\}\right] & \end{cases} \tag{S3.224}$$

  - Left-hand limit for $T_r = 1$

$$\lim_{T_r \nearrow 1} \alpha' = -2c_d = -2\cdot\frac{\frac{m}{2}-p_0(1+p_1+p_2)}{1+\frac{m}{2}-p_0(1+p_1+p_2)} \tag{S3.225}$$

  - Right-hand limit for $T_r = 1$

$$\lim_{T_r \searrow 1} \alpha' = -m+2p_0(1+p_1+p_2) \tag{S3.226}$$

  Equation (S3.225) and Equation (S3.226) yield *a priori* not the same result indicating that alpha(T) is not obliged to be continuous.

- α''

$$\alpha'' = \begin{cases} 4c_d^2 \exp\left\{2c_d(1-T_r)\right\} & T_r \leq 1 \\ 2\cdot\left[1+m(1-\sqrt{T_r})-p_0(1-T_r)(1+p_1T_r+p_2T_r^2)\right] & T_r > 1 \\ \quad \cdot\left[\frac{m}{4T_r^{3/2}}+2p_0(p_1-p_2)+6p_0p_2T_r\right] & \\ \quad +2\cdot\left[-\frac{m}{2\sqrt{T_r}}+p_0\left\{(1-p_1)+2(p_1-p_2)T_r+3p_2T_r^2\right\}\right]^2 & \end{cases} \tag{S3.227}$$

  - Left-hand limit for $T_r = 1$

$$\lim_{T_r \nearrow 1} \alpha'' = 4c_d^2 = 4\cdot\frac{\frac{m}{2}-p_0(1+p_1+p_2)}{1+\frac{m}{2}-p_0(1+p_1+p_2)} \tag{S3.228}$$

  - Right-hand limit for $T_r = 1$

$$\lim_{T_r \searrow 1} \alpha'' = \frac{m}{2}+4p_0p_1+8p_0p_2+2\left(\frac{m}{2}-p_0(1+p_1+p_2)\right)^2 \tag{S3.229}$$

  Equation (S3.228) and Equation (S3.229) yield *a priori* not the same result indicating that alpha(T) is not obliged to be continuous.

S3.5.5. VTBMSR-III

- α

$$\alpha = \begin{cases} \left[1 + m(1 - \sqrt{T_r}) - p_0(1 - T_r)(1 + p_1 T_r + p_2 T_r^2)\right]^2 & T_r \leq 1 \\ \left[\exp\left\{c_d(1 - T_r^d)\right\}\right]^2 & T_r > 1 \end{cases} \tag{S3.230}$$

$$d = 1 + \frac{m}{2} - p_0(1 + p_1 + p_2) \tag{S3.231}$$

$$c_d = 1 - \frac{1}{d} \tag{S3.232}$$

  - Left-hand limit for $T_r = 1$

$$\lim_{T_r \nearrow 1} \alpha = 1 \tag{S3.233}$$

  - Right-hand limit for $T_r = 1$

$$\lim_{T_r \searrow 1} \alpha = 1 \tag{S3.234}$$

Equation (S3.233) and Equation (S3.234) yield the same result. Therefore, $\alpha(T)$ is continuous in $T_r = 1$.

- α'

$$\alpha' = \begin{cases} 2 \cdot \left[1 + m(1 - \sqrt{T_r}) - p_0(1 - T_r)(1 + p_1 T_r + p_2 T_r^2)\right] & T_r \leq 1 \\ \quad \cdot \left[-\frac{m}{2\sqrt{T_r}} + p_0\left\{(1 - p_1) + 2(p_1 - p_2)T_r + 3p_2 T_r^2\right\}\right] & \\ -2c_d d T_r^{d-1} \exp\left\{2c_d(1 - T_r^d)\right\} & T_r > 1 \end{cases} \tag{S3.235}$$

  - Left-hand limit for $T_r = 1$

$$\lim_{T_r \nearrow 1} \alpha' = -m + 2p_0(1 + p_1 + p_2) \tag{S3.236}$$

  - Right-hand limit for $T_r = 1$

$$\lim_{T_r \searrow 1} \alpha' = -2c_d d = -m + 2p_0(1 + p_1 + p_2) \tag{S3.237}$$

Equation (S3.236) and Equation (S3.237) yield the same result. Therefore, $\alpha(T)$ is continuous in $T_r = 1$.

- α''

$$\alpha'' = \begin{cases} 2 \cdot \left[1 + m(1 - \sqrt{T_r}) - p_0(1 - T_r)(1 + p_1 T_r + p_2 T_r^2)\right] & T_r \leq 1 \\ \quad \cdot \left[\frac{m}{4T_r^{3/2}} + 2p_0(p_1 - p_2) + 6p_0 p_2 T_r\right] & \\ \quad +2 \cdot \left[-\frac{m}{2\sqrt{T_r}} + p_0\left\{(1 - p_1) + 2(p_1 - p_2)T_r + 3p_2 T_r^2\right\}\right]^2 & \\ \left(4c_d^2 d^2 T_r^{2(d-1)} - 2c_d d(d - 1)T_r^{d-2}\right) \exp\left\{2c_d(1 - T_r^d)\right\} & T_r > 1 \end{cases} \tag{S3.238}$$

  - Left-hand limit for $T_r = 1$

$$\lim_{T_r \nearrow 1} \alpha'' = \frac{m}{2} + 4p_0 p_1 + 8p_0 p_2 + 2\left(\frac{m}{2} - p_0(1 + p_1 + p_2)\right)^2 \tag{S3.239}$$

  - Right-hand limit for $T_r = 1$

$$\lim_{T_r \searrow 1} \alpha'' = 4c_d^2 d^2 - 2c_d d(d - 1) = 2\left(\frac{m}{2} - p_0(1 + p_1 + p_2)\right)^2 \tag{S3.240}$$

Equation (S3.239) and Equation (S3.240) yield *a priori* not the same result indicating that alpha(T) is not obliged to be continuous.

S3.5.6. Summary

Table S3.1: Summary of the analysis of the continuity of parameter α and its derivatives for different α-functions. ✓: continuous, ✗: discontinuous.

EOS	Continuity		
	α	α'	α''
Boston-Mathias	✓	✓	✗
Mathias	✓	✓	✗
VTBMSR-I	✓	✓	✗
VTBMSR-II	✓	✗	✗
VTBMSR-III	✓	✓	✗

Remark: None of the mentioned EOS fulfills the consistency proposed by Le Guennec *et al.* [37].

S3.5.7. Graphics

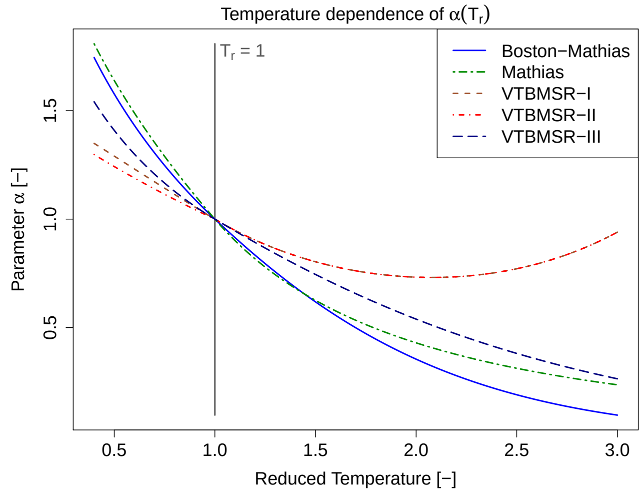

(a) $\alpha(T_r)$ - Water liquid.

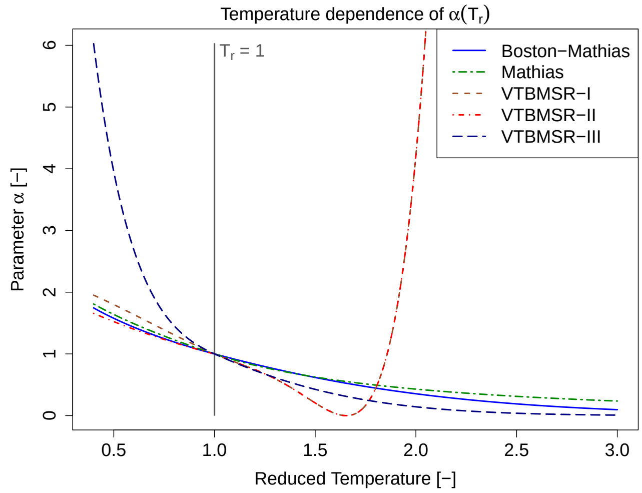

(b) $\alpha(T_r)$ - Water vapor.

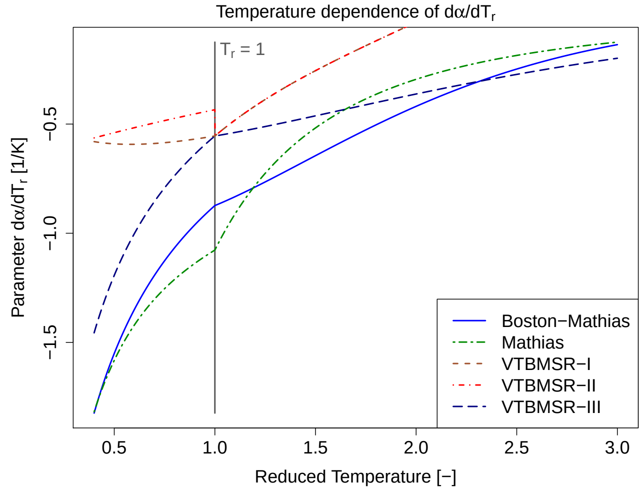

(c) $\alpha'(T_r)$ - Water liquid.

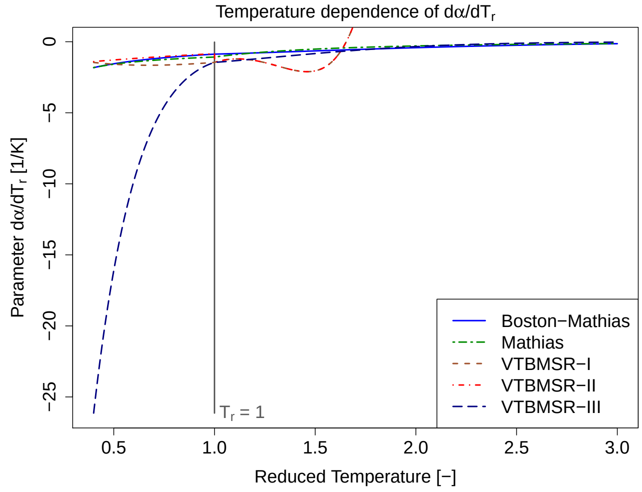

(d) $\alpha'(T_r)$ - Water vapor.

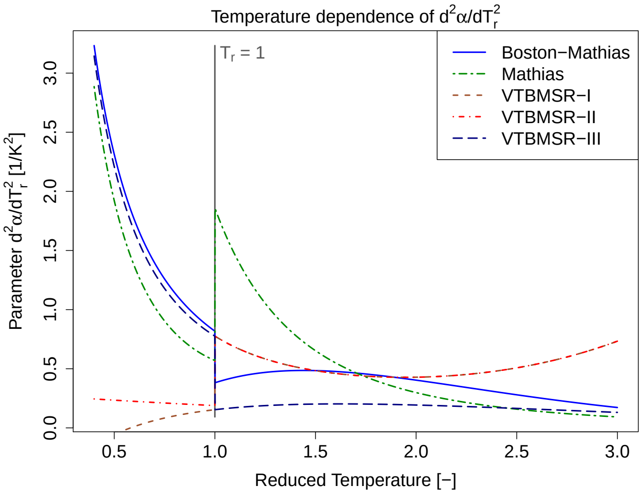

(e) $\alpha''(T_r)$ - Water liquid.

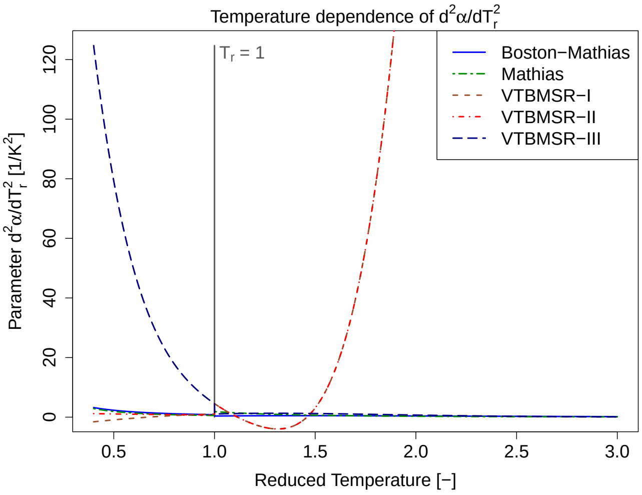

(f) $\alpha''(T_r)$ - Water vapor.

Figure S3.5: Parameter $\alpha(T_r)$ and its first and second derivatives with respect to the reduced temperature T_r for water in liquid and vapor phase.

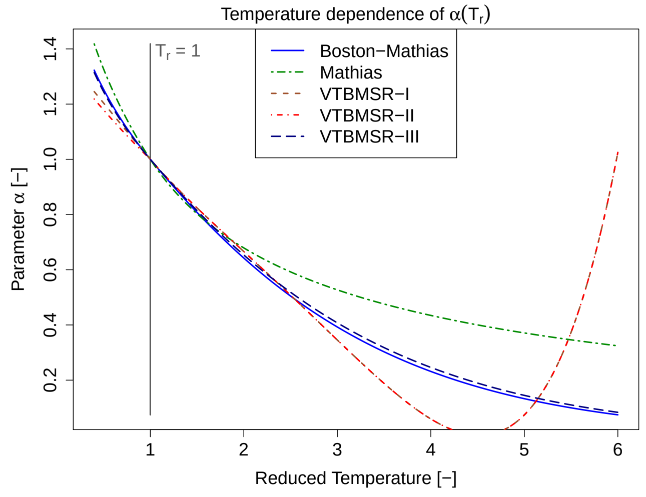

(a) $\alpha(T_r)$ - Oxygen.

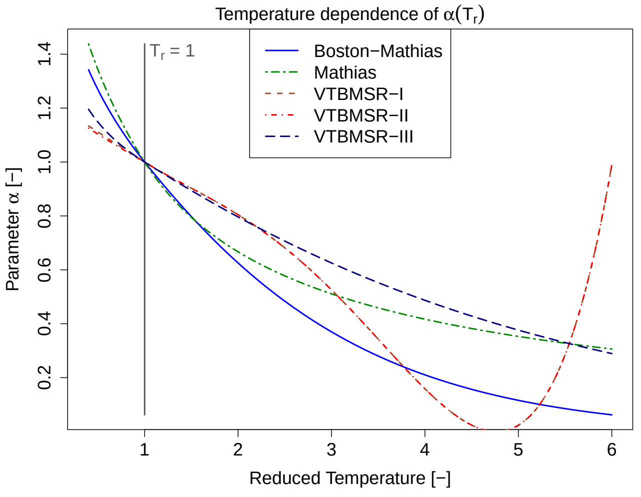

(b) $\alpha(T_r)$ - Nitrogen.

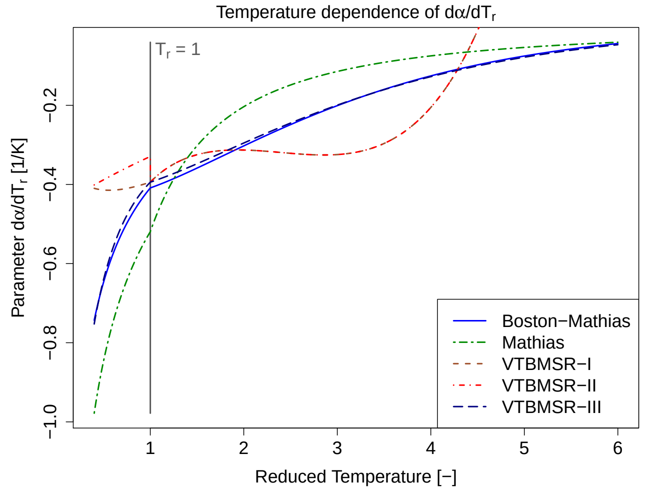

(c) $\alpha'(T_r)$ - Oxygen.

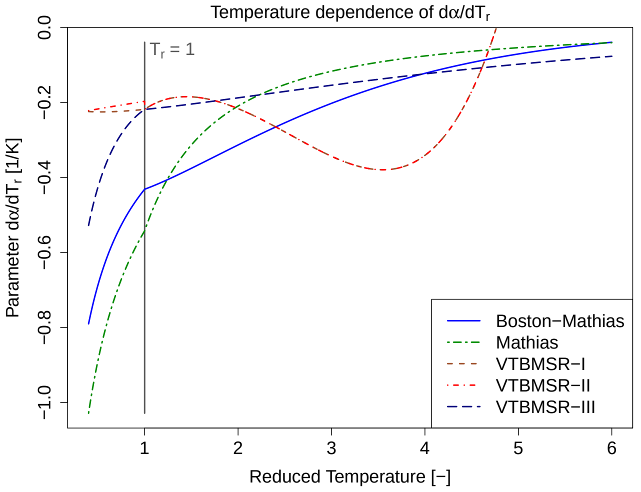

(d) $\alpha'(T_r)$ - Nitrogen.

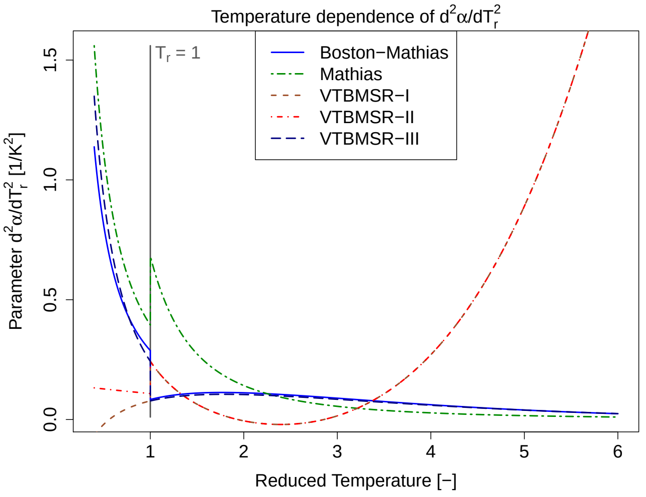

(e) $\alpha''(T_r)$ - Oxygen.

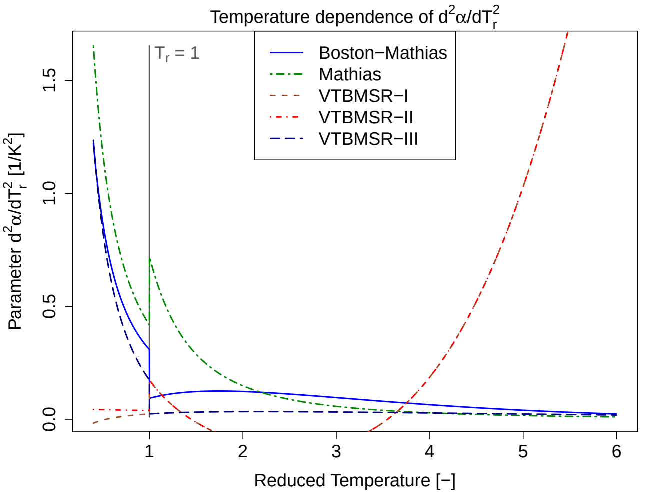

(f) $\alpha''(T_r)$ - Nitrogen.

Figure S3.6: Parameter $\alpha(T_r)$ and its first and second derivatives with respect to the reduced temperature T_r for oxygen and nitrogen.

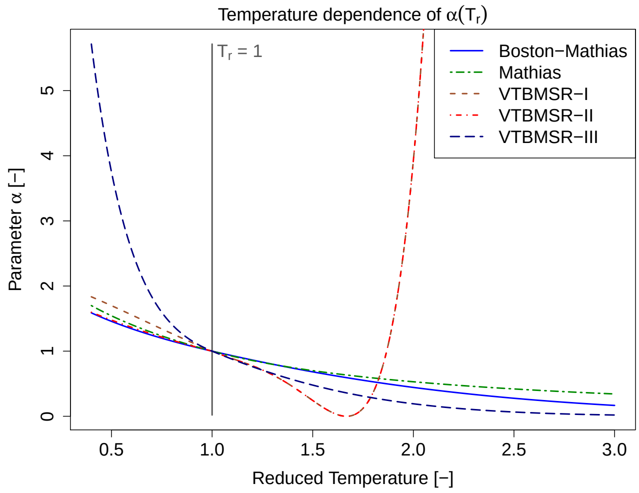

(a) $\alpha(T_r)$ - Carbon dioxide.

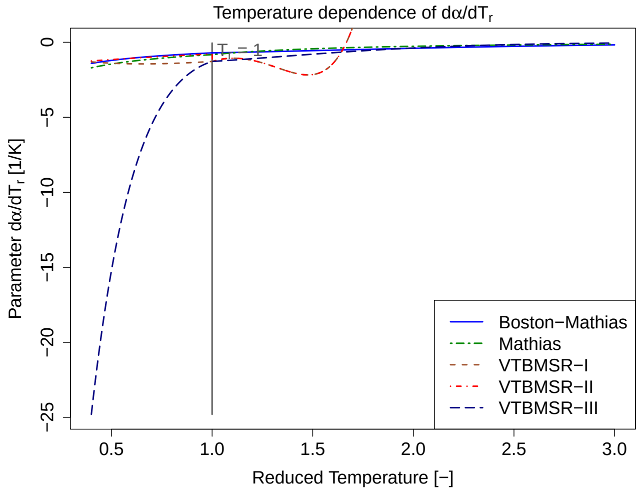

(b) $\alpha'(T_r)$ - Carbon dioxide.

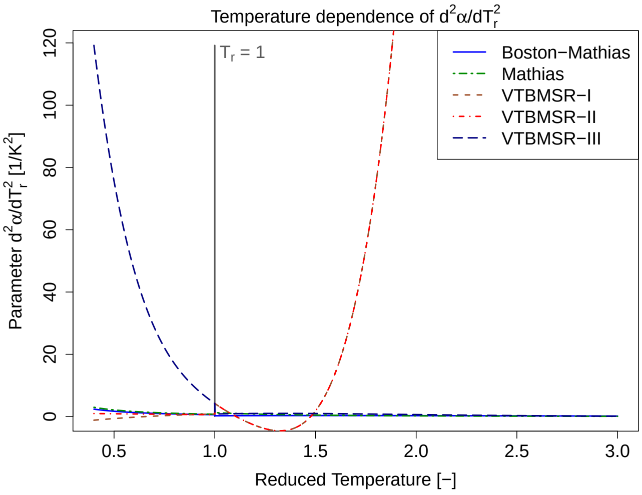

(c) $\alpha''(T_r)$ - Carbon dioxide.

Figure S3.7: Parameter $\alpha(T_r)$ and its first and second derivatives with respect to the reduced temperature T_r for carbon dioxide.

S3.6. *Error Distributions*

S3.6.1. *Averaged Absolute Relative Errors for Pure Substances for Different Equations of State*

Table S3.2: Averaged absolute relative errors and standard deviations [%] between calculated and reference data of pure compounds for the different EOS. The subscripts (l) and (v/sc) indicate the liquid or vapor/supercritical phase of water.

Substance	EOS	PS	v_m [%]	h [%]	c_v [%]	c_p [%]
$H_2O_{(l)}$	VdW		250 ± 150	290 ± 10	57.0 ± 0.2	45.5 ± 0.6
	RK		45.0 ± 0.2	79 ± 2	11.7 ± 0.2	17.0 ± 0.4
	RKS		38.6 ± 0.1	26 ± 1	12.34 ± 0.07	19.4 ± 0.2
	PR		22.9 ± 0.1	15.6 ± 0.7	17.95 ± 0.07	14.0 ± 0.2
	VTBMSR-I	1	8.37 ± 0.09	169 ± 6	28.3 ± 0.5	30.6 ± 0.4
		2	8.06 ± 0.09	165 ± 6	28.0 ± 0.5	30.2 ± 0.4
	VTBMSR-II	1	10.1 ± 0.1	185 ± 6	26.3 ± 0.4	29.5 ± 0.3
		2	9.9 ± 0.1	182 ± 6	25.9 ± 0.4	29.0 ± 0.3
	VTBMSR-III	1	6.6 ± 0.1	98 ± 3	11.7 ± 0.3	1.7 ± 0.1
		2	6.3 ± 0.1	94 ± 3	11.9 ± 0.3	1.8 ± 0.1
$H_2O_{(v/sc)}$	VdW		5.8 ± 0.4	3.1 ± 0.2	19.7 ± 0.5	20.5 ± 0.5
	RK		3.7 ± 0.2	1.8 ± 0.1	15.3 ± 0.4	14.2 ± 0.4
	RKS		3.7 ± 0.3	0.95 ± 0.06	13.3 ± 0.4	9.1 ± 0.3
	PR		2.5 ± 0.2	1.04 ± 0.05	14.2 ± 0.4	9.4 ± 0.3
	VTBMSR-I	1	2.0 ± 0.1	1.49 ± 0.05	30.0 ± 0.7	16.8 ± 0.4
		2	2.6 ± 0.1	1.63 ± 0.06	34.1 ± 0.9	20.2 ± 0.4
		3	5.0 ± 0.1	1.65 ± 0.05	22.2 ± 0.7	7.8 ± 0.4
	VTBMSR-II	1	2.1 ± 0.1	1.68 ± 0.05	30.1 ± 0.7	17.8 ± 0.4
		2	2.7 ± 0.1	1.78 ± 0.06	34.2 ± 0.9	21.0 ± 0.4
		3	5.0 ± 0.1	1.81 ± 0.06	22.2 ± 0.7	8.6 ± 0.4
	VTBMSR-III	1	2.0 ± 0.1	0.82 ± 0.02	8.7 ± 0.3	6.3 ± 0.3
		2	2.3 ± 0.1	0.87 ± 0.03	10.7 ± 0.3	6.7 ± 0.3
		3	2.2 ± 0.1	0.85 ± 0.03	10.4 ± 0.3	6.7 ± 0.3
O_2	VdW		2.09 ± 0.03	0.93 ± 0.01	1.42 ± 0.03	0.72 ± 0.03
	RK		1.22 ± 0.01	0.185 ± 0.005	0.58 ± 0.02	0.525 ± 0.009
	RKS		0.71 ± 0.01	0.497 ± 0.007	1.55 ± 0.02	0.414 ± 0.008
	PR		1.21 ± 0.02	0.46 ± 0.01	0.89 ± 0.01	0.407 ± 0.005
	VTBMSR-I	11	0.85 ± 0.02	2.14 ± 0.05	18.3 ± 0.5	11.0 ± 0.3
		12	43 ± 1	3.8 ± 0.1 × 10^3	3.9 ± 0.1 × 10^4	3.0 ± 0.1 × 10^4
		13	0.87 ± 0.02	2.49 ± 0.06	22.3 ± 0.7	13.3 ± 0.4
		14	45.0 ± 0.9	1.56 ± 0.05 × 10^3	1.30 ± 0.04 × 10^4	1.03 ± 0.03 × 10^4
		15	81.5 ± 0.6	9.3 ± 0.2 × 10^3	7.2 ± 0.2 × 10^4	5.4 ± 0.1 × 10^4
		21	1.11 ± 0.02	1.99 ± 0.05	18.3 ± 0.5	11.0 ± 0.3
		22	42.3 ± 0.9	3.8 ± 0.1 × 10^3	3.9 ± 0.1 × 10^4	3.0 ± 0.1 × 10^4
		23	1.57 ± 0.02	2.41 ± 0.07	22.3 ± 0.7	13.3 ± 0.4
		24	44.9 ± 0.9	1.56 ± 0.05 × 10^3	1.30 ± 0.04 × 10^4	1.03 ± 0.03 × 10^4
		25	80.0 ± 0.6	9.2 ± 0.2 × 10^3	7.2 ± 0.2 × 10^4	5.4 ± 0.1 × 10^4
	VTBMSR-II	11	0.85 ± 0.02	2.14 ± 0.05	18.3 ± 0.5	11.0 ± 0.3
		12	43 ± 1	3.8 ± 0.1 × 10^3	3.9 ± 0.1 × 10^4	3.0 ± 0.1 × 10^4
		13	0.87 ± 0.02	2.49 ± 0.06	22.3 ± 0.7	13.3 ± 0.4
		14	45.0 ± 0.9	1.56 ± 0.05 × 10^3	1.30 ± 0.04 × 10^4	1.03 ± 0.03 × 10^4
		15	81.5 ± 0.6	9.3 ± 0.2 × 10^3	7.2 ± 0.2 × 10^4	5.4 ± 0.1 × 10^4
		21	1.11 ± 0.02	1.99 ± 0.05	18.3 ± 0.5	11.0 ± 0.3
		22	42.3 ± 0.9	3.8 ± 0.1 × 10^3	3.9 ± 0.1 × 10^4	3.0 ± 0.1 × 10^4
		23	1.57 ± 0.02	2.41 ± 0.07	22.3 ± 0.7	13.3 ± 0.4
		24	44.9 ± 0.9	1.56 ± 0.05 × 10^3	1.30 ± 0.04 × 10^4	1.03 ± 0.03 × 10^4
		25	80.0 ± 0.6	9.2 ± 0.2 × 10^3	7.2 ± 0.2 × 10^4	5.4 ± 0.1 × 10^4

PS: Parameter set

Substance	EOS	PS	v_m [%]	h [%]	c_v [%]	c_p [%]
O_2	VTBMSR-III	11	1.15 ± 0.02	0.37 ± 0.01	1.69 ± 0.02	0.83 ± 0.02
		12	0.463 ± 0.008	0.584 ± 0.008	2.78 ± 0.05	1.10 ± 0.03
		13	0.78 ± 0.01	0.353 ± 0.009	2.05 ± 0.03	0.89 ± 0.02
		14	0.543 ± 0.009	0.668 ± 0.009	2.92 ± 0.05	1.16 ± 0.03
		15	0.487 ± 0.009	0.611 ± 0.008	2.83 ± 0.05	1.12 ± 0.03
		21	0.418 ± 0.008	0.376 ± 0.006	1.69 ± 0.02	0.83 ± 0.02
		22	1.50 ± 0.03	0.99 ± 0.01	2.78 ± 0.05	1.10 ± 0.03
		23	0.78 ± 0.01	0.517 ± 0.007	2.05 ± 0.03	0.89 ± 0.02
		24	1.64 ± 0.03	1.10 ± 0.02	2.92 ± 0.05	1.16 ± 0.03
		25	1.55 ± 0.03	1.03 ± 0.01	2.83 ± 0.05	1.12 ± 0.03
N_2	VdW		2.14 ± 0.02	1.24 ± 0.01	1.66 ± 0.03	0.63 ± 0.03
	RK		1.99 ± 0.02	0.325 ± 0.005	0.42 ± 0.01	0.74 ± 0.01
	RKS		0.421 ± 0.008	0.611 ± 0.008	1.76 ± 0.03	0.360 ± 0.008
	PR		1.62 ± 0.02	0.427 ± 0.008	1.03 ± 0.02	0.295 ± 0.005
	VTBMSR-I	1	0.80 ± 0.01	0.338 ± 0.005	0.69 ± 0.02	0.53 ± 0.01
		2	3.4 ± 0.1	10.2 ± 0.3	109 ± 4	75 ± 3
	VTBMSR-II	1	0.80 ± 0.01	0.338 ± 0.005	0.69 ± 0.02	0.53 ± 0.01
		2	3.4 ± 0.1	10.2 ± 0.3	109 ± 4	75 ± 3
	VTBMSR-III	1	0.326 ± 0.005	0.446 ± 0.006	1.97 ± 0.04	0.84 ± 0.02
		2	1.95 ± 0.03	0.97 ± 0.02	0.458 ± 0.008	0.552 ± 0.007
CO_2	VdW		4.86 ± 0.07	0.93 ± 0.02	3.9 ± 0.1	3.7 ± 0.2
	RK		2.49 ± 0.03	0.388 ± 0.007	0.92 ± 0.06	1.37 ± 0.07
	RKS		3.14 ± 0.05	0.371 ± 0.007	2.43 ± 0.05	1.40 ± 0.03
	PR		0.72 ± 0.02	0.414 ± 0.008	1.96 ± 0.06	1.39 ± 0.04
	VTBMSR-I	1	74.8 ± 0.8	$3.2 \pm 0.1 \times 10^5$	$3.8 \pm 0.1 \times 10^6$	$3 \pm 1 \times 10^6$
		2	8.7 ± 0.4	40 ± 3	670 ± 50	990 ± 80
	VTBMSR-II	1	74.8 ± 0.8	$3.2 \pm 0.1 \times 10^5$	$3.8 \pm 0.1 \times 10^6$	$3 \pm 1 \times 10^6$
		2	8.7 ± 0.4	40 ± 3	670 ± 50	990 ± 80
	VTBMSR-III	1	3.07 ± 0.05	0.69 ± 0.01	4.9 ± 0.2	2.60 ± 0.08
		2	1.68 ± 0.03	0.483 ± 0.008	3.48 ± 0.07	2.11 ± 0.04

PS: Parameter set

Table S3.3: Averaged absolute relative errors and standard deviations [%] between calculated and reference data of pure compounds for the different EOS considering the best-fitting parameter set. The subscripts (l) and (v/sc) indicate the liquid or vapor/supercritical phase of water.

Substance	EOS	v_m [%]	h [%]	c_v [%]	c_p [%]
$H_2O_{(l)}$ (PS 2)	VdW	250 ± 150	290 ± 10	57.0 ± 0.2	45.5 ± 0.6
	RK	45.0 ± 0.2	79 ± 2	11.7 ± 0.2	17.0 ± 0.4
	RKS	38.6 ± 0.1	26 ± 1	12.34 ± 0.07	19.4 ± 0.2
	PR	22.9 ± 0.1	15.6 ± 0.7	17.95 ± 0.07	14.0 ± 0.2
	VTBMSR-I	8.06 ± 0.09	165 ± 6	28.0 ± 0.5	30.2 ± 0.4
	VTBMSR-II	9.9 ± 0.1	182 ± 6	25.9 ± 0.4	29.0 ± 0.3
	VTBMSR-III	6.3 ± 0.1	94 ± 3	11.9 ± 0.3	1.8 ± 0.1
$H_2O_{(v/sc)}$ (PS 3)	VdW	5.8 ± 0.4	3.1 ± 0.2	19.7 ± 0.5	20.5 ± 0.5
	RK	3.7 ± 0.2	1.8 ± 0.1	15.3 ± 0.4	14.2 ± 0.4
	RKS	3.7 ± 0.3	0.95 ± 0.06	13.3 ± 0.4	9.1 ± 0.3
	PR	2.5 ± 0.2	1.04 ± 0.05	14.2 ± 0.4	9.4 ± 0.3
	VTBMSR-I	5.0 ± 0.1	1.65 ± 0.05	22.2 ± 0.7	7.8 ± 0.4
	VTBMSR-II	5.0 ± 0.1	1.81 ± 0.06	22.2 ± 0.7	8.6 ± 0.4
	VTBMSR-III	2.2 ± 0.1	0.85 ± 0.03	10.4 ± 0.3	6.7 ± 0.3
O_2 (PS 21)	VdW	2.09 ± 0.03	0.93 ± 0.01	1.42 ± 0.03	0.72 ± 0.03
	RK	1.22 ± 0.01	0.185 ± 0.005	0.58 ± 0.02	0.525 ± 0.009
	RKS	0.71 ± 0.01	0.497 ± 0.007	1.55 ± 0.02	0.414 ± 0.008
	PR	1.21 ± 0.02	0.46 ± 0.01	0.89 ± 0.01	0.407 ± 0.005
	VTBMSR-I	1.11 ± 0.02	1.99 ± 0.05	18.3 ± 0.5	11.0 ± 0.3
	VTBMSR-II	1.11 ± 0.02	1.99 ± 0.05	18.3 ± 0.5	11.0 ± 0.3
	VTBMSR-III	0.418 ± 0.008	0.376 ± 0.006	1.69 ± 0.02	0.83 ± 0.02
N_2 (PS 1)	VdW	2.14 ± 0.02	1.24 ± 0.01	1.66 ± 0.03	0.63 ± 0.03
	RK	1.99 ± 0.02	0.325 ± 0.005	0.42 ± 0.01	0.74 ± 0.01
	RKS	0.421 ± 0.008	0.611 ± 0.008	1.76 ± 0.03	0.360 ± 0.008
	PR	1.62 ± 0.02	0.427 ± 0.008	1.03 ± 0.02	0.295 ± 0.005
	VTBMSR-I	0.80 ± 0.01	0.338 ± 0.005	0.69 ± 0.02	0.53 ± 0.01
	VTBMSR-II	0.80 ± 0.01	0.338 ± 0.005	0.69 ± 0.02	0.53 ± 0.01
	VTBMSR-III	0.326 ± 0.005	0.446 ± 0.006	1.97 ± 0.04	0.84 ± 0.02
CO_2 (PS 2)	VdW	4.86 ± 0.07	0.93 ± 0.02	3.9 ± 0.1	3.7 ± 0.2
	RK	2.49 ± 0.03	0.388 ± 0.007	0.92 ± 0.06	1.37 ± 0.07
	RKS	3.14 ± 0.05	0.371 ± 0.007	2.43 ± 0.05	1.40 ± 0.03
	PR	0.72 ± 0.02	0.414 ± 0.008	1.96 ± 0.06	1.39 ± 0.04
	VTBMSR-I	8.7 ± 0.4	40 ± 3	670 ± 50	990 ± 80
	VTBMSR-II	8.7 ± 0.4	40 ± 3	670 ± 50	990 ± 80
	VTBMSR-III	1.68 ± 0.03	0.483 ± 0.008	3.48 ± 0.07	2.11 ± 0.04

Table S3.4: Averaged absolute relative errors and standard deviations [%] between calculated and reference data of pure compounds for the different VTBMSR-EOS. The subscripts (l) and (v/sc) indicate the liquid or vapor/supercritical phase of water.

Substance	EOS	PS	v_m [%]	h [%]	c_v [%]	c_p [%]
$H_2O_{(l)}$	VTBMSR-I	1	8.37 ± 0.09	169 ± 6	28.3 ± 0.5	30.6 ± 0.4
		2	8.06 ± 0.09	165 ± 6	28.0 ± 0.5	30.2 ± 0.4
	VTBMSR-II	1	10.1 ± 0.1	185 ± 6	26.3 ± 0.4	29.5 ± 0.3
		2	9.9 ± 0.1	182 ± 6	25.9 ± 0.4	29.0 ± 0.3
	VTBMSR-III	1	6.6 ± 0.1	98 ± 3	11.7 ± 0.3	1.7 ± 0.1
		2	6.3 ± 0.1	94 ± 3	11.9 ± 0.3	1.8 ± 0.1
$H_2O_{(v/sc)}$	VTBMSR-I	1	2.0 ± 0.1	1.49 ± 0.05	30.0 ± 0.7	16.8 ± 0.4
		2	2.6 ± 0.1	1.63 ± 0.06	34.1 ± 0.9	20.2 ± 0.4
		3	5.0 ± 0.1	1.65 ± 0.05	22.2 ± 0.7	7.8 ± 0.4
	VTBMSR-II	1	2.1 ± 0.1	1.68 ± 0.05	30.1 ± 0.7	17.8 ± 0.4
		2	2.7 ± 0.1	1.78 ± 0.06	34.2 ± 0.9	21.0 ± 0.4
		3	5.0 ± 0.1	1.81 ± 0.06	22.2 ± 0.7	8.6 ± 0.4
	VTBMSR-III	1	2.0 ± 0.1	0.82 ± 0.02	8.7 ± 0.3	6.3 ± 0.3
		2	2.3 ± 0.1	0.87 ± 0.03	10.7 ± 0.3	6.7 ± 0.3
		3	2.2 ± 0.1	0.85 ± 0.03	10.4 ± 0.3	6.7 ± 0.3
O_2	VTBMSR-I	11	0.85 ± 0.02	2.14 ± 0.05	18.3 ± 0.5	11.0 ± 0.3
		12	43 ± 1	3.8 ± 0.1 $\times 10^3$	3.9 ± 0.1 $\times 10^4$	3.0 ± 0.1 $\times 10^4$
		13	0.87 ± 0.02	2.49 ± 0.06	22.3 ± 0.7	13.3 ± 0.4
		14	45.0 ± 0.9	1.56 ± 0.05 $\times 10^3$	1.30 ± 0.04 $\times 10^4$	1.03 ± 0.03 $\times 10^4$
		15	81.5 ± 0.6	9.3 ± 0.2 $\times 10^3$	7.2 ± 0.2 $\times 10^4$	5.4 ± 0.1 $\times 10^4$
		21	1.11 ± 0.02	1.99 ± 0.05	18.3 ± 0.5	11.0 ± 0.3
		22	42.3 ± 0.9	3.8 ± 0.1 $\times 10^3$	3.9 ± 0.1 $\times 10^4$	3.0 ± 0.1 $\times 10^4$
		23	1.57 ± 0.02	2.41 ± 0.07	22.3 ± 0.7	13.3 ± 0.4
		24	44.9 ± 0.9	1.56 ± 0.05 $\times 10^3$	1.30 ± 0.04 $\times 10^4$	1.03 ± 0.03 $\times 10^4$
		25	80.0 ± 0.6	9.2 ± 0.2 $\times 10^3$	7.2 ± 0.2 $\times 10^4$	5.4 ± 0.1 $\times 10^4$
	VTBMSR-II	11	0.85 ± 0.02	2.14 ± 0.05	18.3 ± 0.5	11.0 ± 0.3
		12	43 ± 1	3.8 ± 0.1 $\times 10^3$	3.9 ± 0.1 $\times 10^4$	3.0 ± 0.1 $\times 10^4$
		13	0.87 ± 0.02	2.49 ± 0.06	22.3 ± 0.7	13.3 ± 0.4
		14	45.0 ± 0.9	1.56 ± 0.05 $\times 10^3$	1.30 ± 0.04 $\times 10^4$	1.03 ± 0.03 $\times 10^4$
		15	81.5 ± 0.6	9.3 ± 0.2 $\times 10^3$	7.2 ± 0.2 $\times 10^4$	5.4 ± 0.1 $\times 10^4$
		21	1.11 ± 0.02	1.99 ± 0.05	18.3 ± 0.5	11.0 ± 0.3
		22	42.3 ± 0.9	3.8 ± 0.1 $\times 10^3$	3.9 ± 0.1 $\times 10^4$	3.0 ± 0.1 $\times 10^4$
		23	1.57 ± 0.02	2.41 ± 0.07	22.3 ± 0.7	13.3 ± 0.4
		24	44.9 ± 0.9	1.56 ± 0.05 $\times 10^3$	1.30 ± 0.04 $\times 10^4$	1.03 ± 0.03 $\times 10^4$
		25	80.0 ± 0.6	9.2 ± 0.2 $\times 10^3$	7.2 ± 0.2 $\times 10^4$	5.4 ± 0.1 $\times 10^4$
	VTBMSR-III	11	1.15 ± 0.02	0.37 ± 0.01	1.69 ± 0.02	0.83 ± 0.02
		12	0.463 ± 0.008	0.584 ± 0.008	2.78 ± 0.05	1.10 ± 0.03
		13	0.78 ± 0.01	0.353 ± 0.009	2.05 ± 0.03	0.89 ± 0.02
		14	0.543 ± 0.009	0.668 ± 0.009	2.92 ± 0.05	1.16 ± 0.03
		15	0.487 ± 0.009	0.611 ± 0.008	2.83 ± 0.05	1.12 ± 0.03
		21	0.418 ± 0.008	0.376 ± 0.006	1.69 ± 0.02	0.83 ± 0.02
		22	1.50 ± 0.03	0.99 ± 0.01	2.78 ± 0.05	1.10 ± 0.03
		23	0.78 ± 0.01	0.517 ± 0.007	2.05 ± 0.03	0.89 ± 0.02
		24	1.64 ± 0.03	1.10 ± 0.02	2.92 ± 0.05	1.16 ± 0.03
		25	1.55 ± 0.03	1.03 ± 0.01	2.83 ± 0.05	1.12 ± 0.03
N_2	VTBMSR-I	1	0.80 ± 0.01	0.338 ± 0.005	0.69 ± 0.02	0.53 ± 0.01
		2	3.4 ± 0.1	10.2 ± 0.3	109 ± 4	75 ± 3
	VTBMSR-II	1	0.80 ± 0.01	0.338 ± 0.005	0.69 ± 0.02	0.53 ± 0.01
		2	3.4 ± 0.1	10.2 ± 0.3	109 ± 4	75 ± 3
	VTBMSR-III	1	0.326 ± 0.005	0.446 ± 0.006	1.97 ± 0.04	0.84 ± 0.02
		2	1.95 ± 0.03	0.97 ± 0.02	0.458 ± 0.008	0.552 ± 0.007
CO_2	VTBMSR-I	1	74.8 ± 0.8	3.2 ± 0.1 $\times 10^5$	3.8 ± 0.1 $\times 10^6$	3 ± 1 $\times 10^6$
		2	8.7 ± 0.4	40 ± 3	670 ± 50	990 ± 80
	VTBMSR-II	1	74.8 ± 0.8	3.2 ± 0.1 $\times 10^5$	3.8 ± 0.1 $\times 10^6$	3 ± 1 $\times 10^6$
		2	8.7 ± 0.4	40 ± 3	670 ± 50	990 ± 80
	VTBMSR-III	1	3.07 ± 0.05	0.69 ± 0.01	4.9 ± 0.2	2.60 ± 0.08
		2	1.68 ± 0.03	0.483 ± 0.008	3.48 ± 0.07	2.11 ± 0.04

PS: Parameter set

S3.6.2. Relative Error in the Prediction of the Molar Volume of Liquid Water

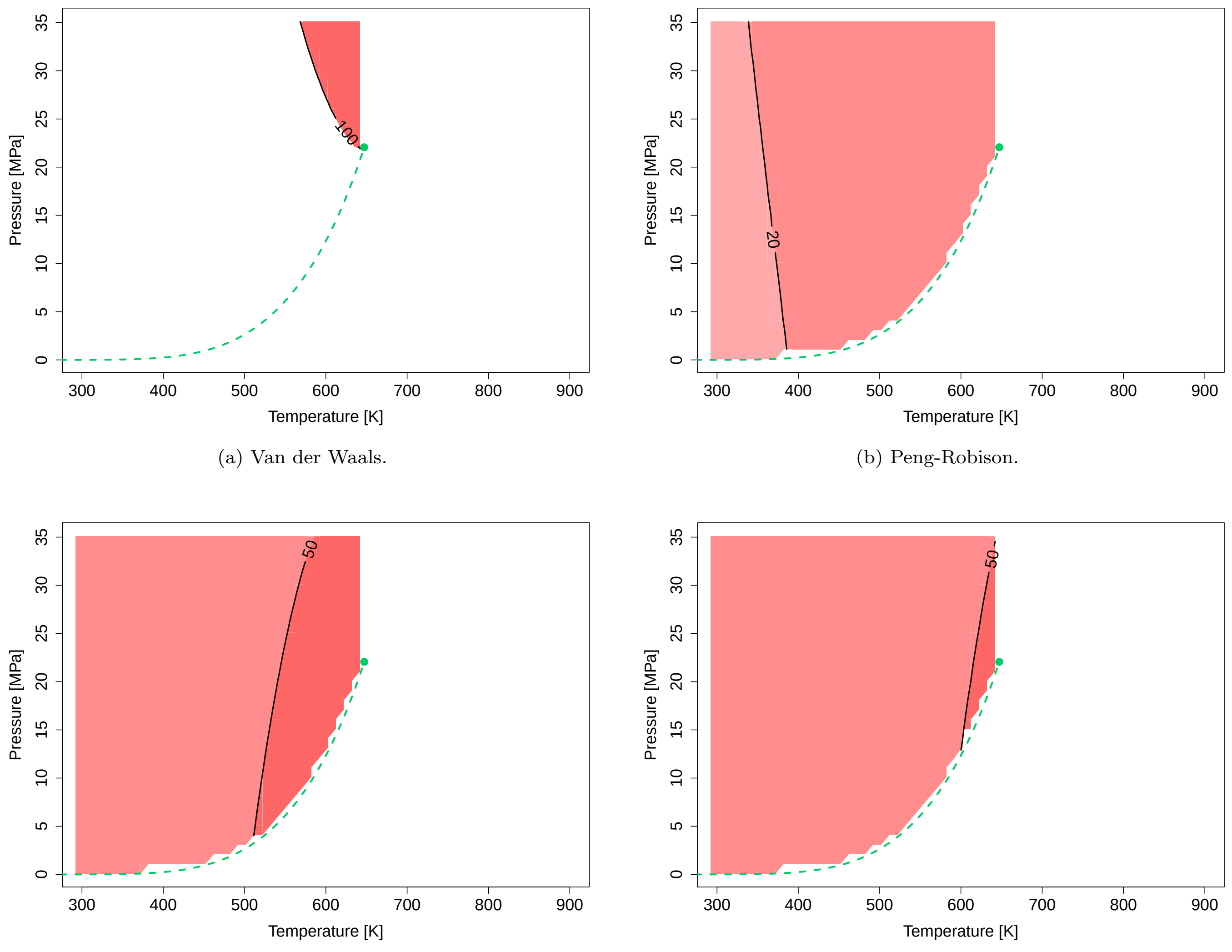

(a) Van der Waals.

(b) Peng-Robison.

(c) Redlich-Kwong.

(d) Redlich-Kwong-Soave.

Figure S3.8: Isocontour plots of constant relative error [%] for the molar volume [m^3/mol] of liquid water calculated by different equations of state based on Refprop reference data [27]. The dashed green line and the green dot denote the vapor-liquid coexistence curve and the critical point of water, respectively.

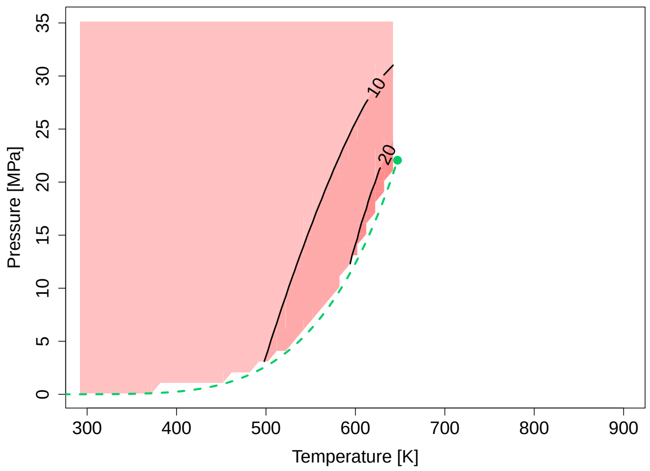

(e) VTBMSR-I.

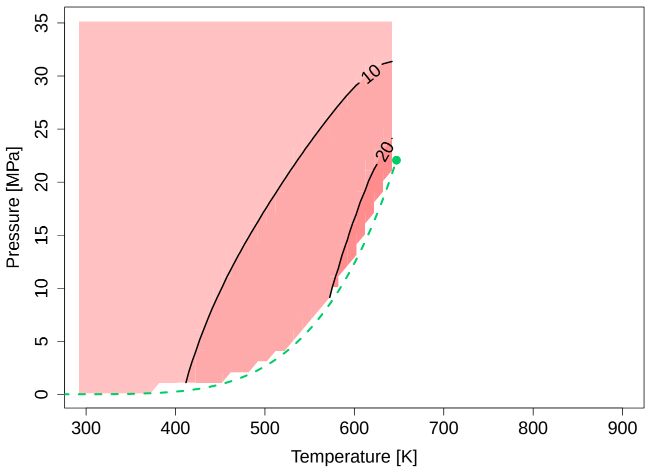

(f) VTBMSR-II.

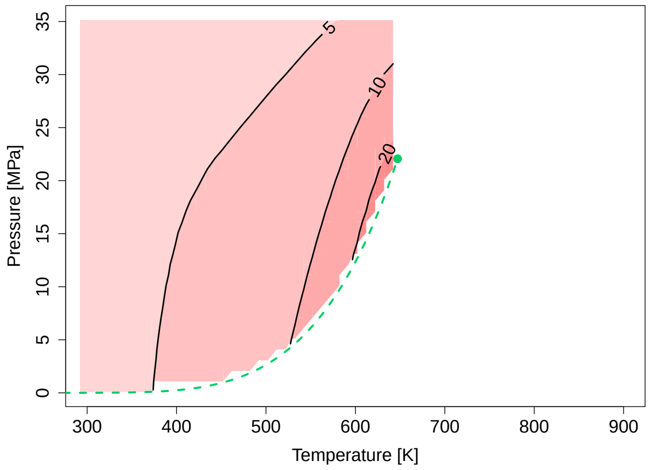

(g) VTBMSR-III.

Figure S3.8: Isocontour plots of constant relative error [%] for the molar volume [m^3/mol] of liquid water calculated by different equations of state based on Refprop reference data [27]. The dashed green line and the green dot denote the vapor-liquid coexistence curve and the critical point of water, respectively.

S3.6.3. Relative Error in the Prediction of the Molar Volume of Water Vapor

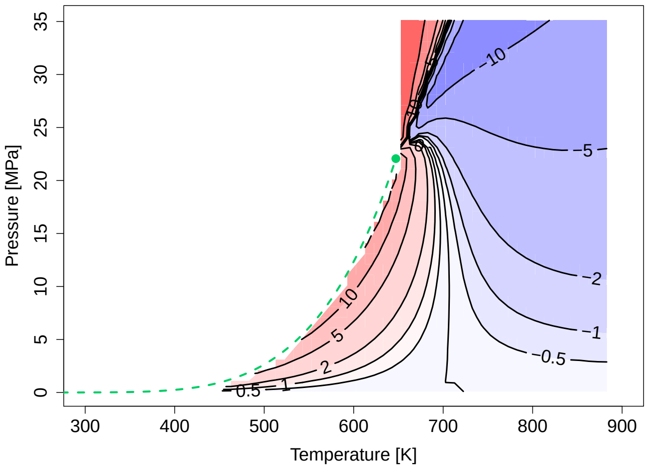

(a) Van der Waals.

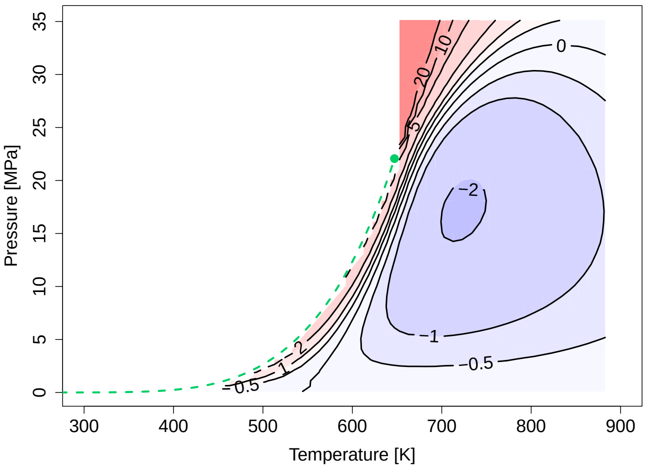

(b) Peng-Robison.

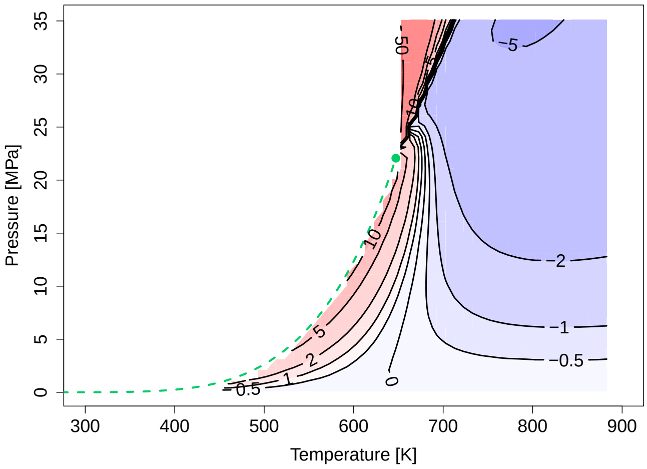

(c) Redlich-Kwong.

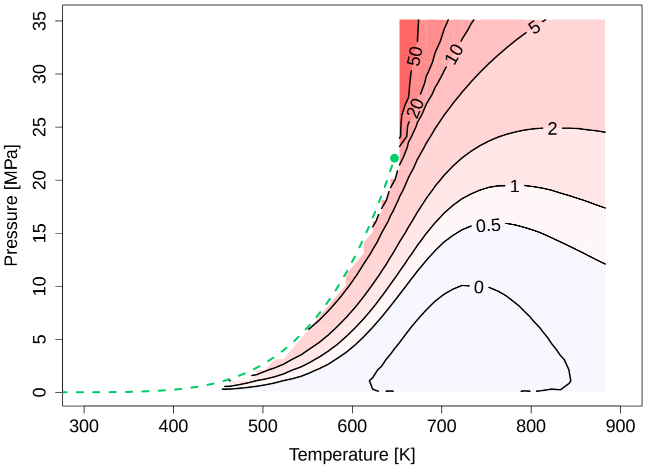

(d) Redlich-Kwong-Soave.

Figure S3.9: Isocontour plots of constant relative error [%] for the molar volume [m^3/mol] of water vapor calculated by different equations of state based on Refprop reference data [27]. The dashed green line and the green dot denote the vapor-liquid coexistence curve and the critical point of water, respectively.

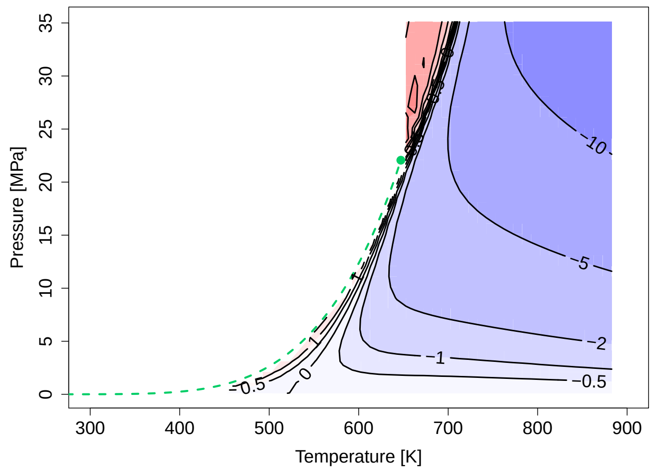

(e) VTBMSR-I.

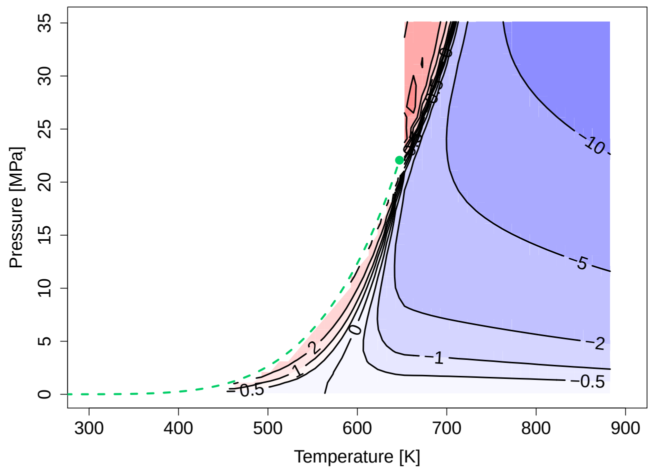

(f) VTBMSR-II.

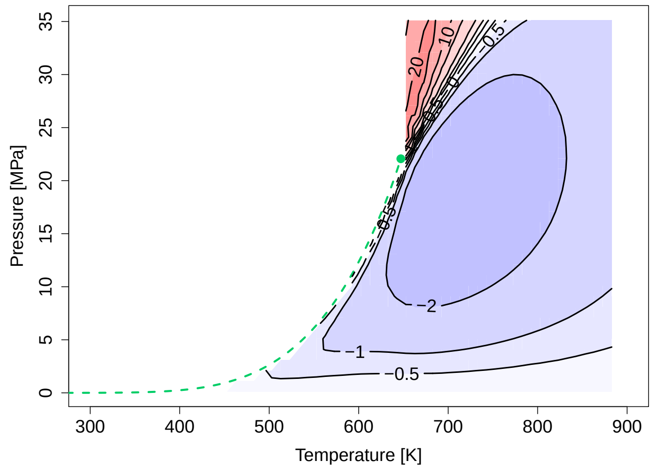

(g) VTBMSR-III.

Figure S3.9: Isocontour plots of constant relative error [%] for the molar volume [m^3/mol] of water vapor calculated by different equations of state based on Refprop reference data [27]. The dashed green line and the green dot denote the vapor-liquid coexistence curve and the critical point of water, respectively.

S3.6.4. Averaged Absolute Relative Errors for Binary Mixtures for Different Equations of State

For the different parameter sets (volume translation, polar, and interaction) the particular best-fitting parameter sets are applied.

Table S3.5: Averaged absolute relative errors and standard deviations of molar volume [%] between calculated and reference data of binary mixtures for the different EOS.

	H_2O/O_2 [30]	H_2O/N_2 [29]	H_2O/N_2 [31]	H_2O/CO_2 [32]	N_2/CO_2 [33]
VdW	22 ± 2	21 ± 2	3.1 ± 0.5	5 ± 1	3.9 ± 0.3
RK	9 ± 1	7 ± 1	2.2 ± 0.5	2.6 ± 0.6	2.6 ± 0.2
RKS	11 ± 1	10 ± 1	3.7 ± 0.5	2.5 ± 0.5	2.1 ± 0.2
PR	7.4 ± 0.7	5.4 ± 0.5	2.5 ± 0.5	1.7 ± 0.4	1.9 ± 0.2
VTBMSR-I	15 ± 1	8 ± 1	2.9 ± 0.5	2.8 ± 0.5	1.9 ± 0.2
VTBMSR-II	15 ± 1	8 ± 1	3.0 ± 0.5	3.2 ± 0.5	1.9 ± 0.2
VTBMSR-III	3.0 ± 0.3	6.6 ± 0.6	1.7 ± 0.4	0.7 ± 0.3	1.1 ± 0.2

S3.6.5. Relative Error in the Prediction of the Molar Volume of H_2O/O_2 *mixture*

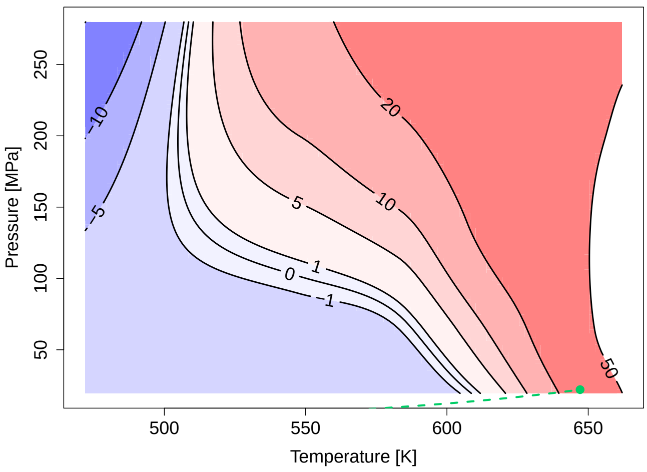

(a) Van der Waals.

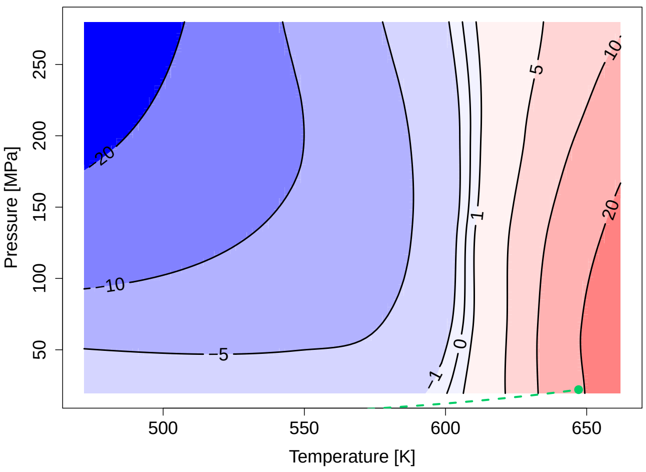

(b) Peng-Robison.

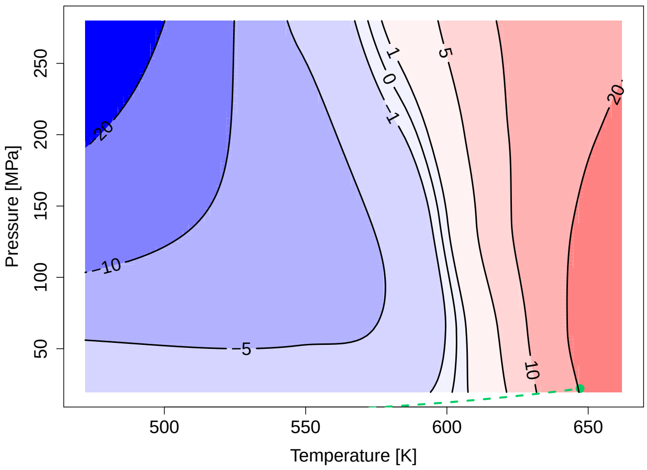

(c) Redlich-Kwong.

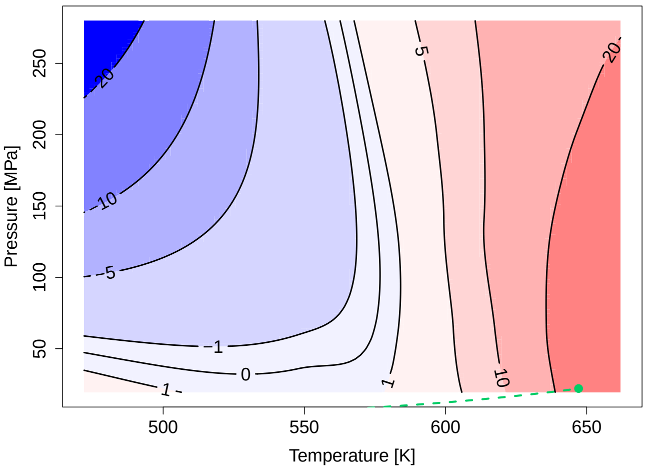

(d) Redlich-Kwong-Soave.

Figure S3.10: Isocontour plots of constant relative error [%] for the molar volume [m^3/mol] of H_2O/O_2 calculated by different equations of state based on Japas and Franck [29]. The dashed green line and the green dot denote the vapor-liquid coexistence curve and the critical point of water, respectively.

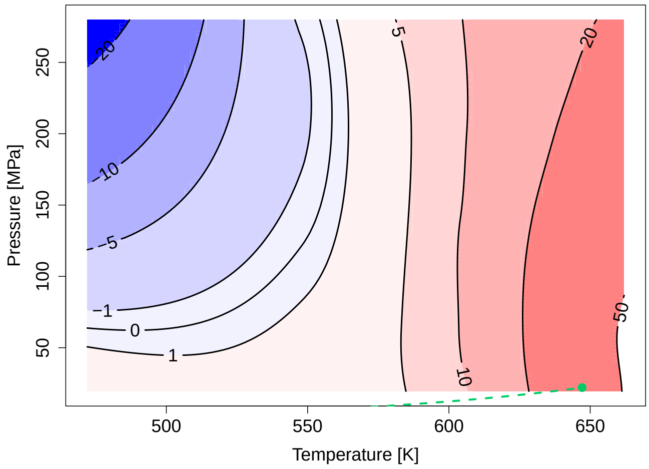

(e) VTBMSR-I.

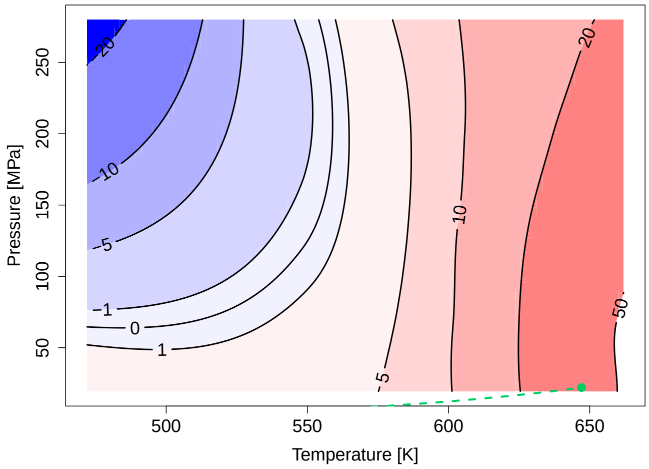

(f) VTBMSR-II.

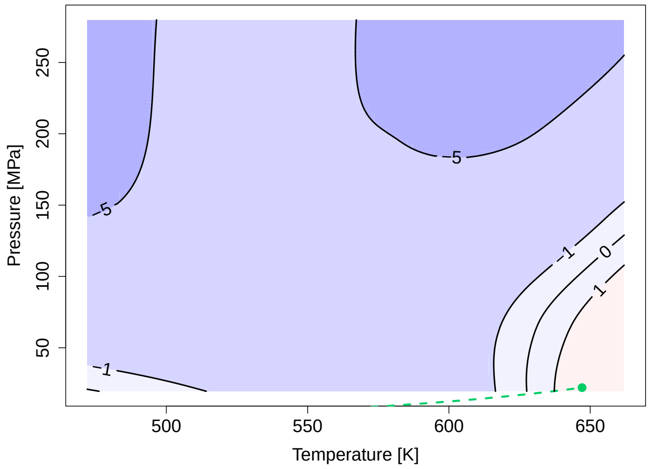

(g) VTBMSR-III.

Figure S3.10: Isocontour plots of constant relative error [%] for the molar volume [m^3/mol] of H_2O/O_2 calculated by different equations of state based on Japas and Franck [29]. The dashed green line and the green dot denote the vapor-liquid coexistence curve and the critical point of water, respectively.

S4. Regression

S4.1. Database for regression

The subsequent reference data is used for the regression of the EOS [11].

S4.1.1. Pure compounds

- Water

  [A] NBS/NRC Steam Table, 1984 [Haar et al. 1984]
  Haar, L.; Gallagher, J.S.; Kell, G.S.: NBS/NRC Steam Tables, Hemisphere Publ. Corp.; Washington, 1984

- Oxygen

  [B] Nat. Std. Ref. Data of the USSR, 1987 [Sychev et al. 1987]
  Sychev, V.V.; Vassermann, A.A.; Kozlov, A.D.; Spiridonov, G.A.; Tsymarny, V.A.: Thermodynamic Properties of Oxygen; Nat. Std. Ref. Data Service of the USSR; Hemisphere Publ. Corp.; Washington, 1987

  [C] IUPAC, 1976 [Wagner & de Rueck 1976]
  Wagner, W.; de Rueck, K.M.: International Tables of the Fluid State - 9: Oxygen; IUPAC; Pergamon Press; London, 1976

- Nitrogen

  [D] IUPAC, 1977 [Angus et al. 1977]
  Angus, S.; de Rueck, K.M.; Armstrong, B.: International Tables of the Fluid State - 6: Nitrogen; IUPAC; Pergamon Press; London, 1977

- Carbon dioxide

  [E] IUPAC, 1973 [Angus et al. 1973]
  Angus, S.; de Rueck, K.M.; Armstrong, B.: International Tables of the Fluid State: Carbon Dioxide; IUPAC; Pergamon Press; London, 1973

S4.1.2. Binary mixtures

- H_2O/O_2
  - Japas and Franck [30]
- H_2O/N_2
  - Japas and Franck [29]
  - Gallagher *et al.* [31]
- H_2O/CO_2
  - Gallagher *et al.* [32]
- N_2/CO_2
  - Johns *et al.* [33]
